# Hypothalamic Menin regulates systemic aging and cognitive decline

**DOI:** 10.1371/journal.pbio.3002033

**Published:** 2023-03-16

**Authors:** Lige Leng, Ziqi Yuan, Xiao Su, Zhenlei Chen, Shangchen Yang, Meiqin Chen, Kai Zhuang, Hui Lin, Hao Sun, Huifang Li, Maoqiang Xue, Jun Xu, Jingqi Yan, Zhenyi Chen, Tifei Yuan, Jie Zhang

**Affiliations:** 1 Fujian Provincial Key Laboratory of Neurodegenerative Disease and Aging Research, Institute of Neuroscience, College of Medicine, Xiamen University, Xiamen, Fujian, China; 2 College of Basic Medicine, Hebei Medical University, Shijiazhuang, Hebei, China; 3 Department of Basic Medical Science, School of Medicine, Xiamen University, Xiamen, Fujian, China; 4 Institute for AI in Medicine, School of Automation, Nanjing University of Information Science and Technology, Nanjing, China; 5 Center for Gene Regulation in Health and Disease, Cleveland State University, Cleveland, Ohio, United States of America; 6 Department of Anesthesiology, First Affiliated Hospital of Xiamen University, Xiamen, Fujian, China; 7 Shanghai Mental Health Center, Shanghai Jiaotong University School of Medicine, Shanghai China; Icahn School of Medicine at Mount Sinai, UNITED STATES

## Abstract

Aging is a systemic process, which is a risk factor for impaired physiological functions, and finally death. The molecular mechanisms driving aging process and the associated cognitive decline are not fully understood. The hypothalamus acts as the arbiter that orchestrates systemic aging through neuroinflammatory signaling. Our recent findings revealed that Menin plays important roles in neuroinflammation and brain development. Here, we found that the hypothalamic Menin signaling diminished in aged mice, which correlates with systemic aging and cognitive deficits. Restoring Menin expression in ventromedial nucleus of hypothalamus (VMH) of aged mice extended lifespan, improved learning and memory, and ameliorated aging biomarkers, while inhibiting Menin in VMH of middle-aged mice induced premature aging and accelerated cognitive decline. We further found that Menin epigenetically regulates neuroinflammatory and metabolic pathways, including D-serine metabolism. Aging-associated Menin reduction led to impaired D-serine release by VMH-hippocampus neural circuit, while D-serine supplement rescued cognitive decline in aged mice. Collectively, VMH Menin serves as a key regulator of systemic aging and aging-related cognitive decline.

## Abstract 2

This study reveals a role for hypothalamic Menin in organismal aging in mice, finding that Menin expression decreases in hypothalamic neurons during aging; mimicking this decrease induces premature aging phenotypes, while restoring Menin expression in the hypothalamus of aged mice ameliorates cognitive and systemic features of aging.

## Introduction

Aging is characterized by the progressive and overall deterioration of physiological functions, leading to the end of an organism’s lifespan [1–4]. Among brain and the sub brain region, hypothalamus has been identified as critical central regulators of aging process [5,6]. Specifically, neuroinflammatory IKKβ/NF-κB signaling in ventromedial hypothalamus (VMH) [5] is defined as the pace-regulator of systemic aging. RelA (p65) phosphorylation, marker of NF-κB activation, increases gradually during aging. Activation of IKKβ/NF-κB signaling tunes down gonadotropin-releasing hormone (GnRH) release in the hypothalamus during aging, while GnRH supplement alleviates aging-impaired neurogenesis and decelerates aging.

Besides aging, VMH is critical in regulating food intake and maintaining whole-body energy metabolic, glucose, and lipid balance [7]. VMH microinflammation has been tightly linked to the metabolic mode of macronutrients, especially the fatty acid-mediated oxidative metabolism [8,9]. Saturated fatty acids (SFAs) activate Toll-like receptor 4 (TLR4) and its adaptor myeloid differentiation factor 88 (MyD88) in VMH, leading to activation of IKKβ/NF-κB [10,11], which subsequently drove the expression of inflammatory genes. However, the upstream molecules regulating neuroinflammation in VMH remain unclear.

Hypothalamic tumor necrosis factor (TNF) receptor mediates adaptive immunity in response to lipopolysaccharide (LPS), peptidoglycan and lipoteichoic acids, or damage-associated molecular patterns (DAMPs) [12]. Immune cytokines, including interleukin-6 (IL-6) and IL-1β, are important in inducing local inflammation [13]. Apart from cytokine, TLRs represent a large family of pattern recognition receptors (PRRs), among which TLR4 is found to be related to obesity-related hypothalamic inflammation [11]. Both NF-κB classical or atypical activation is crucial for initiation, maintenance, and progression of hypothalamic inflammation [5,9]. Our recent findings reported that multiple endocrine neoplasia type 1 (*MEN1*; protein: Menin) associates with p65 to inhibit NF-κB transactivation [14], acting as a crucial factor in inhibiting neuroinflammation. Therefore, we hypothesize that Menin may act as upstream arbiter for aging-associated neuroinflammation in VMH.

In this study, we characterized the changes of Menin expression along with aging in C57BL/6 mice. Then, we manipulated Menin levels selectively in steroidogenic factor-1 (SF-1) neuron of VMH to observe its potential effects on systemic aging and cognitive functions. We then investigated the neuroinflammation cascades and cognitive changes underlying these circumstances. The results explored a novel role of Menin in regulating systemic aging and cognitive function.

## Results

### Reduced Menin in the hypothalamus accelerates systemic aging

**Fig 1:**
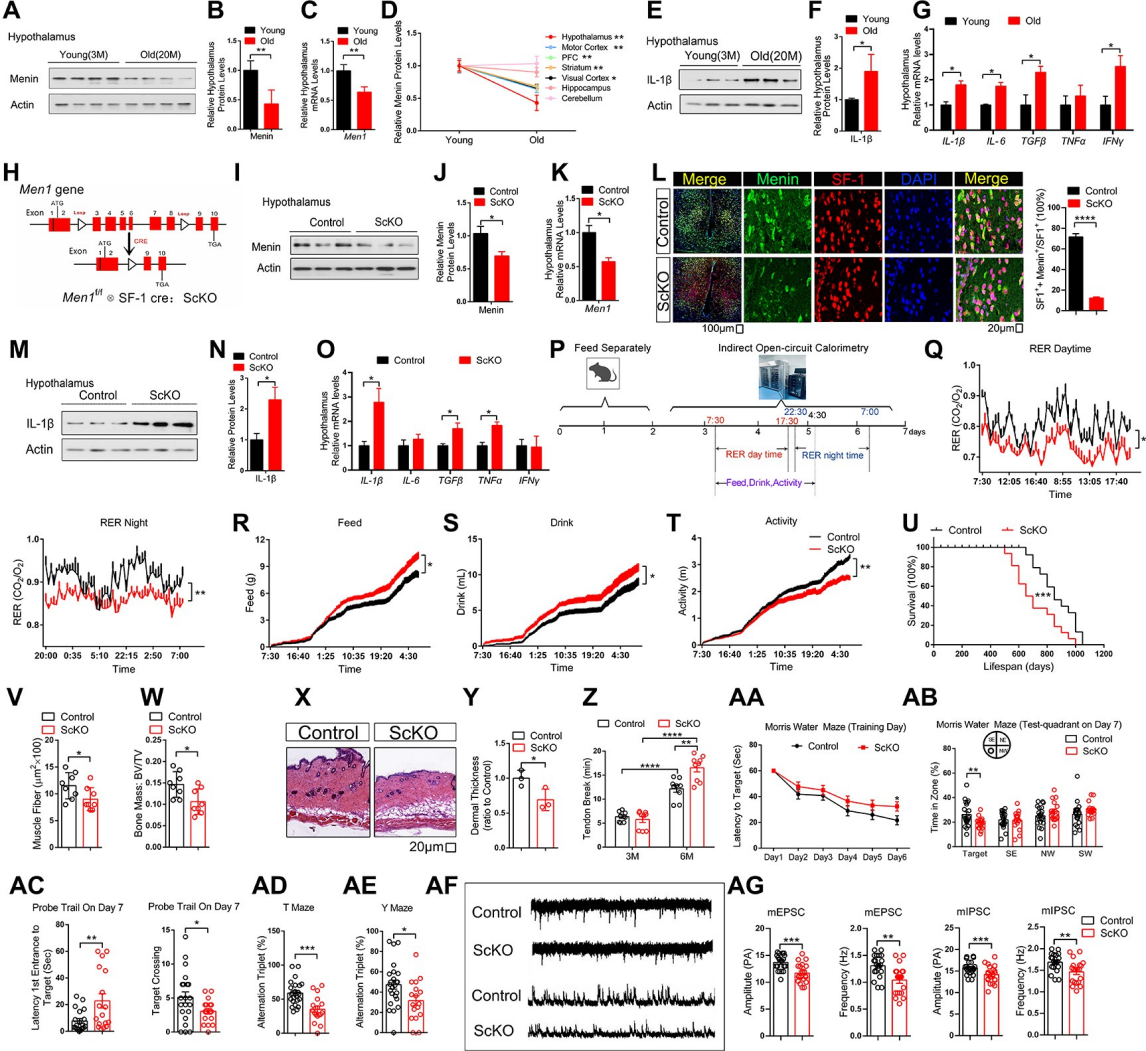
Reduced Menin in the hypothalamus accelerates systemic aging. (A–C) Menin protein expression and mRNA levels in the hypothalamus of young (3M) and old (20M) mice; *n* = 4 mice. (D) Menin protein expression levels in 7 regions of young (3M) and old (20M) mice brain; *n* = 4 mice. Actin serves as a loading control. (E–G) Inflammatory factors protein expression and mRNA levels in the hypothalamus of young (3M) and old (20M) mice; *n* = 3 mice. (H) Generation of conditional *Men1*-knockout mice by crossing *Men1*^f/f^ mice with *SF1*-Cre mice (ScKO). (I–K) The protein and mRNA levels of Menin in the hypothalamus of 6 months ScKO and control mice; *n* = 3 mice. (L) Representative hypothalamus brain sections from 6 months control and ScKO mice stained with Menin (green) and SF1 (red) antibody. Section was counterstained with DAPI (blue). Scale bar, 100 μm, 20 μm; *n* = 5 mice. (M–O) Inflammatory factors protein expression and mRNA levels in the hypothalamus of ScKO (6M) and control (6M) mice; *n* = 3 mice. (P) Schematic diagram of open circuit indirect calorimetry. (Q) Day and night respiratory quotients (RER) of age-matched male ScKO mice (10M) and control mice (10M) were measured. Exact dietary (R), water intake (S), and activity (T) were also measured. (U) Lifespan of these mice (Control mice, *n* = 14; ScKO mice, *n* = 16). (V–Z) Male mice were sacrificed at 10 months for measuring muscle (quadriceps) fiber size (V), bone mass (2), dermal thickness (X, Y), and tail tendon breaking time (Z); *n* = 3~9 mice. Scale bar, 20 μm. (AA–AC) Ten months male ScKO and age-matched control mice behavior in Morris water maze tests. (AD) Ten months male ScKO and control mice behavior in T maze. (AE) Ten months male ScKO and control mice behavior in Y maze. (AF, AG) Electrophysiological recording from ScKO and control mice. Representative whole-cell recordings on SF-1 neurons in the hypothalamus of ScKO and control mice are shown on panel AF. Quantitation of their mEPSC and mIPSC frequency and amplitude are showed in panel AG (*n* ≥ 20 cells from 3 mice). Mouse number used in measuring energy expenditure by open circuit indirect calorimetry: ScKO mice: *n* = 16 mice; Control mice: *n* = 16 mice. Mouse number used in behavior tests: Control: *n* = 24 mice, ScKO mice: *n* = 17 mice. Data represent mean ± SEM, n.s.: not significant, *p < 0.05, **p < 0.01, ***p < 0.001, Kaplan–Meier survival estimate for survival curve. Unpaired *t* test for behavioral statistics. Statistical applications between groups across multiple time points were analyzed by repeated-measures ANOVA. Other statistical applications were analyzed by one-way ANOVA with Tukey’s post hoc analysis. The underlying data of Fig 1 can be found in S1 Information. mEPSC, miniature excitatory postsynaptic current; mIPSC, miniature inhibitory postsynaptic current; SF-1, steroidogenic factor-1.

Inflammatory activation mediated by IKKβ/NF-κB in the hypothalamus was much studied in the context of aging, and further induces the cognitive impairment associated with aging [5]. In previous study, we found that Menin associates with p65 to inhibit nuclear factor kappa-B (NF-κB) transactivation [14]. Therefore, we first examined the trends of Menin in 7 brain regions of young and old mice, and found that the decrease of Menin in the hypothalamus was the most significant with age (Figs 1A–1D and S1A–S1L), which accompanies increased neuroinflammation in the hypothalamus (Fig 1E–1G). Next, by co-immunostaining Menin with SF-1, GFAP, and IBA1, we found that the expression of Menin decreased significantly only in VMH SF-1 neurons but not in astrocytes and microglia in aged mice brain (S1M–S1R Fig). These results suggested that the function of Menin in SF-1 neuron may be strongly implicated in aging. The expression of Menin gradually decreases with aging, and is still relatively high at 10M, when is choose for subsequent experiments (S1S and S1T Fig). Previous studies have implicated SF-1 neurons, which are exclusive to hypothalamic VMH, as important metabolic regulators [7]. VMH has been shown to regulate the maintenance of energy homeostasis in whole body, VMH SF-1 neurons can respond to the nutritional status [15–17]. To understand the potential relevance of reduced Menin on hypothalamic neuroinflammation and systemic aging, we generated steroidogenic factor 1 (SF1) Cre *Men1*^f/f^ conditional knockout mice (ScKO) by crossing SF1-Cre mice [18] with mice carrying floxed *Men1* alleles (*Men1*^f/f^) [19] (Fig 1H). Menin deletion efficiency in SF-1 neurons was confirmed by western blotting, real time-PCR, and immunostaining (Fig 1I–1L). Mice with SF-1 neuronal Menin knockdown show increased neuroinflammation in VMH (Fig 1M–1O). Menin deletion in SF-1 neuron subsequently induced ScKO mice exhibit irregular metabolic circadian rhythm, increased food and water intake accompany with increased body weight (Figs 1P–1T and S2B). ScKO mice are born at an expected mendelian frequency with a nearly 1:1 sex ratio, without significant changes in body weight, brain weight, and neuron number in hypothalamus and hippocampus (S2A–S2E Fig). However, the lifespan in ScKO mice is significantly decreased when compared to control animals in both male and female mice (Figs 1U and S3J). The ScKO mice also exhibit aging-related phenotypes including reduced muscle fiber size, bone mass, skin thickness, tail tendon collagen cross-linking, clock genes expression, increased ventricular muscle thickness, and DNA methylation levels (Figs 1V–1Z and S2F–S2I). β-Gal staining on the liver, heart, muscle, and hypothalamus of above mice indicated that the above tissues from ScKO mice had premature aging (S2J–S2Q Fig). In addition, cognitive decline as the important aging phenotype appeared in 10-month-old male and female ScKO mice as measured with Morris water maze. ScKO mice showed impaired learning with increased escape latency compared with controls during the 6-day training phase. Moreover, during the probe trial test, in which the hidden platform was removed on day 7, ScKO mice spent significantly less time in the target quadrant. ScKO mice also completed fewer entries into the platform location and needed a longer period to travel from the entry point to the target zone compared with controls. Moreover, ScKO mice also showed reduced spontaneous alternations compared to controls in T- and Y-maze tests (Figs 1AA–1AE and S3N–S3U). We did not observe significant difference in locomotion, anxiety- and depression-related behavior tests between control and ScKO mice (S3A–S3I, S3K–S3M, S3S, and S3T Figs). By electrophysiology, we found that VMH SF-1 neurons in ScKO mice showed decreased amplitude and frequency of sEPSC and sIPSC versus control mice (Fig 1AF and 1AG).

**Fig 2:**
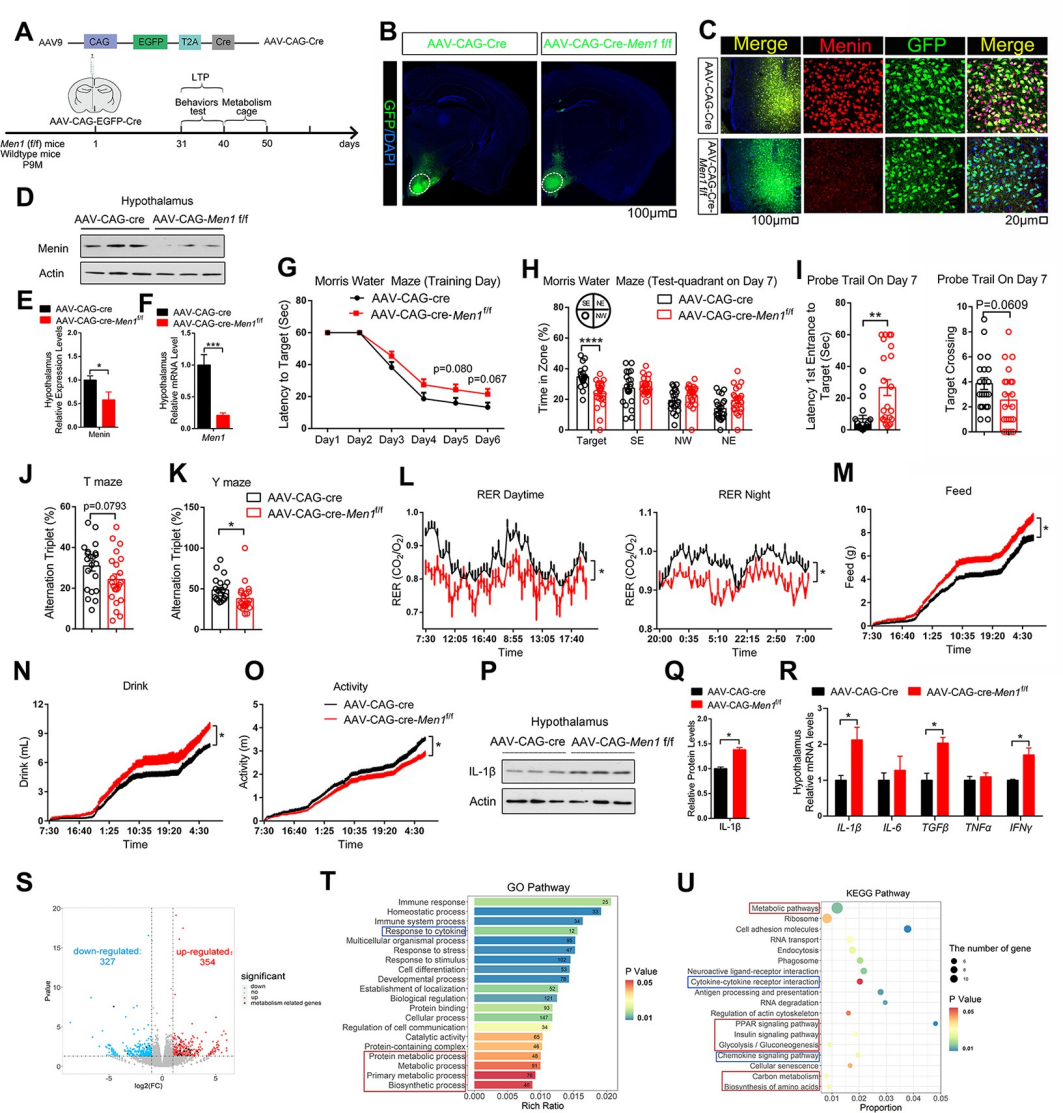
Menin knockdown virus in VMH leads to deficient spatial memory and metabolic disorder. (A) Schematic diagram of knockdown of *Men1* by injection of Cre-dependent CAG-GFP-AAV in VMH of *Men1*^f/f^ mice and control mice (injection at 9M) that are defined as AAV-CAG-Cre and AV-CAG-Cre-*Men1*^f/f^. (B) AAV-GFP immunofluorescence staining in mouse hypothalamus following AAV injection. Scale bar, 100 μm. (C) Representative hypothalamus brain sections from 10 months male AAV-CAG-Cre–injected WT mice and AAV-CAG-Cre–injected *Men1*^f/f^ mice stained with Menin (red) and GFP (green) antibody. Section was counterstained with DAPI (blue). Scale bar, 100 μm, 20 μm. (D–F) The protein and mRNA levels of Menin in the hypothalamus from 10 months old AAV-CAG-Cre–injected WT and *Men1*^f/f^ mice; *n* = 3 mice. (G–K) Morris water maze (G–I), T maze (J) and Y maze (K) tests were performed in 10 months male AAV-CAG-Cre–injected WT and *Men1*^f/f^ mice (AAV-CAG-Cre/AAV-CAG-Cre-*Men1*^f/f^). During Morris water maze tests, 10 months male AAV-CAG-Cre–injected WT and *Men1*^f/f^ mice were analyzed for escape latency during a 6-day training period (G). On the next day, mice were analyzed for time spent in the target zone and other quadrants (northeast, southeast, and northwest) (H), number of target crossings and time required from entrance to the target platform (I). (L) Day and night respiratory quotients (RER) of 10 months male AAV-CAG-Cre mice and age-matched AAV-CAG-Cre-*Men1*^f/f^ mice were measured. Exact dietary (M), water intake (N), and activity (O) were also measured. (P–R) Inflammatory factors protein expression and mRNA levels in the hypothalamus of 10 months male AAV-CAG-Cre mice and AAV-CAG-Cre-*Men1*^f/f^ mice; *n* = 3 mice. (S–U) DEGs were identified from the hypothalamus of 13 months male ScKO mice and control mice and were shown in panel S. DEGs then were analyzed by GO pathway (T) and KEGG pathway (U). The red box represents the enriched metabolic pathway. The blue box represents the enriched cytokine pathway; *n* = 3 mice. Mouse number used in measuring energy expenditure by open circuit indirect calorimetry: AAV-CAG-Cre mice: *n* = 8 mice; AAV-CAG-Cre-*Men1*^f/f^ mice: *n* = 8 mice. Mouse number used in behavior tests: AAV-CAG-Cre mice: *n* = 21 mice; AAV-CAG-Cre-*Men1*^f/f^ mice: *n* = 22 mice. Data represent mean ± SEM, n.s.: not significant, *p < 0.05, **p < 0.01, ***p < 0.001. Unpaired *t* test for behavioral statistics. Statistical applications between groups across multiple time points were analyzed by repeated-measures ANOVA. Other statistical applications were analyzed by one-way ANOVA with Tukey’s post hoc analysis. The underlying data of Fig 2 can be found in S2 Information. DEG, differentially expressed gene; VMH, ventromedial hypothalamus.

SF1 also expresses in the adrenal glands, pituitary, sexual gland, and spleen [20,21], cre-mediated recombination and deletion of Menin may be “eyombi in these tissue in SF1 Cre *Men1*^f/f^ mice. We indeed found that the Menin levels were decreased in these glands (S4 Fig). To exclude the effects of Menin deletion in other glands, we injected cre recombinase-dependent virus (AAV-CAG-Cre) into the VMH of *Men1*^f/f^ and WT mice to specifically delete Menin in VMH region (Fig 2A and 2B). Menin knockdown in neurons of VMH of AAV-CAG-Cre-*Men1*^f/f^ mice without significant toxicity that results neuronal death were confirmed by western blotting, real time-PCR, and immunostaining (Figs 2C–2F, S5E, S5F, and S5H–S5K). AAV-CAG-Cre-*Men1*^f/f^ mice also have a decreased lifespan without significant changes in body weight, brain weight, and neuron number in hypothalamus and hippocampus compared to AAV-CAG-Cre–injected control mice (S5A–S5D and S5G Fig). We also conducted open circuit indirect calorimetry, the hypothalamic inflammation level tests, cognitive behavioral experiments, and found that AAV-CAG-Cre-*Men1*^f/f^ mice showed similar results to ScKO mice (Fig 2G–2R). These results suggest that Menin deficiency in the hypothalamus accelerate aging, potentially through the enhanced hypothalamic inflammation.

### Menin deficiency results in disrupted metabolism in the hypothalamus

To gain a deeper insight into how *Men1* deletion in VMH regulates systematic aging, we then performed next-generation sequencing to identify differentially expressed genes (DEGs) in VMH of 13-month-old control and ScKO mice (S2 Fig and S1 Dataset). The analysis revealed a total of 681 genes with differential expression, including *Acox2*, *B3gnt5*, *Ugtla6a*, and *Pklr* that are involved in metabolic pathway (Fig 2T and 2U).

**Fig 3:**
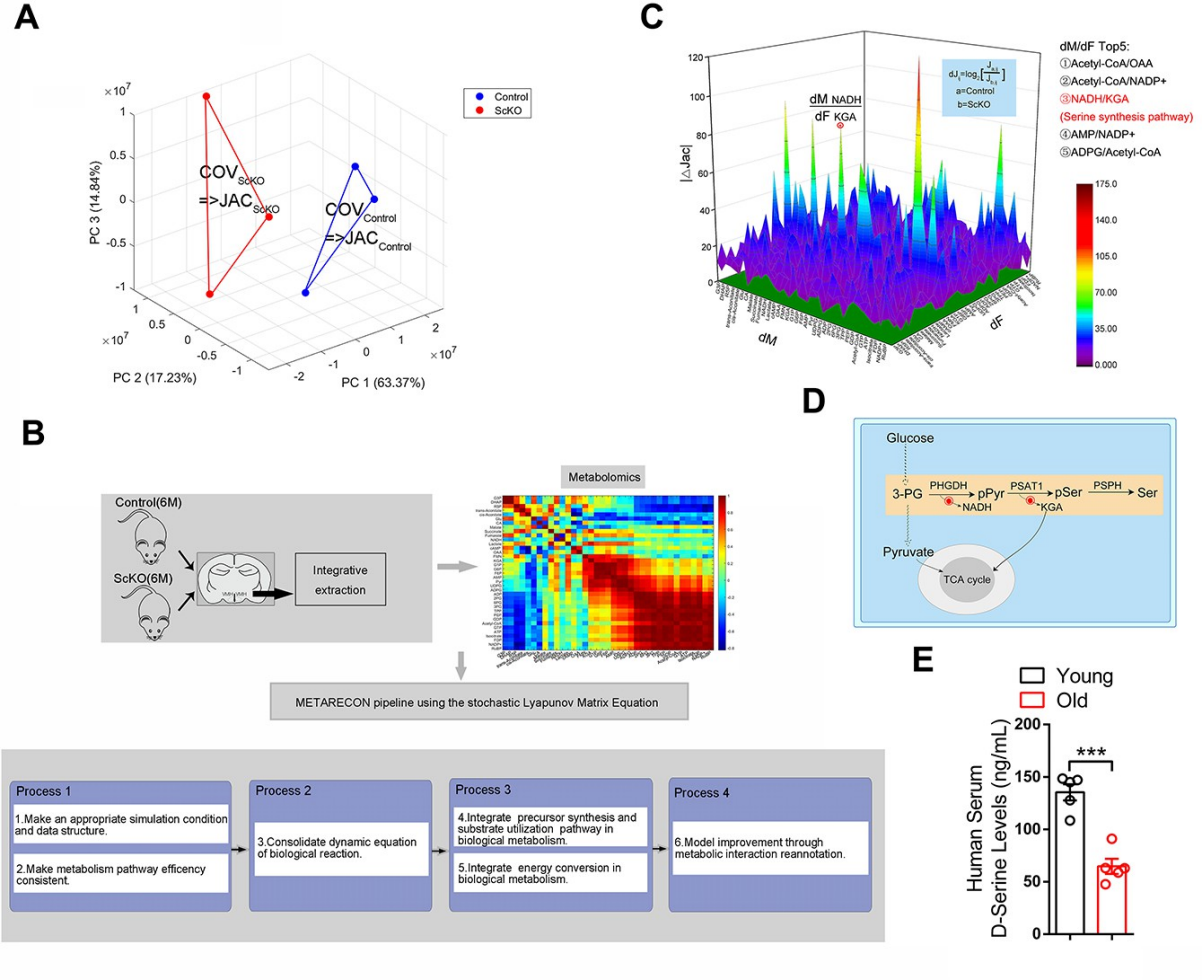
Menin deficiency results in disrupted metabolism in the hypothalamus. (A) PCA of the metabolomics data revealing an obvious separation between the hypothalamus of 13-month-old ScKO mice and control mice. (B) For inverse modeling of biochemical regulation from metabolomics covariance data, a metabolic reconstruction and pathway reduction from metabonomics is performed (RECON). The metabolite variance of 2 groups is visible, which is further exploited for the calculation of the Covariance matrix (COV) and subsequently for the Jacobian matrix (JAC) using the stochastic Lyapunov matrix Eq 1 (see the METARECON strategy in Materials and methods). (C) Differential Jacobian matrix of the hypothalamus of 13-month-old ScKO mice and control mice derived from covariance data from the metabolomics datasets. All entries represent median values of 10^3^ calculations normalized to the square of interquartile distance. *dF* and *dM* characterize the entries of the Jacobian matrix and refer to Eq 2 (see the METARECON strategy in Materials and methods). The greatest perturbation was identified as the Jacobian entry *ðf*_*NADH*_ *= ðf*_*KGA*_, pointing to D-serine synthesis pathway in the underlying biochemical network in panel D. (D) Simplified biochemical interaction network of D-serine synthesis pathway adjusted to the measured metabolites. (E) D-serine levels in serum from young (22-26Y) and old individuals (83-94Y) were determined by ELISA; *n* = 5. Data represent mean ± SEM, n.s.: not significant, *p < 0.05, **p < 0.01, ***p < 0.001. Statistical applications were analyzed by one-way ANOVA with Tukey’s post hoc analysis. The underlying data of Fig 3 can be found in S3 Information. PCA, principal component analysis.

To elucidate detailed changes in metabolomics, we screened 40 major metabolites (S6 Fig) and performed METARECON [22,23] analysis. The METARECON strategy, which incorporates the covariance data matrix of the measured metabolite profiles from VMH of ScKO and control mice in conjunction with Eq 1, was used to identify metabolic perturbation points. The largest perturbation in the differential Jacobian, when comparing VMH of ScKO versus controls, combined with the targeted metabolomics, was detected for the reaction rate elasticity of NADH to KEG (*ðf NADH/ðf KEG*) (Fig 3C), which the changes in D-serine synthesis pathway (Fig 3A–3D) was highlighted in ScKO mice. Moreover, reduction in D-serine levels were found in serum of old individuals (Fig 3E).

**Fig 4:**
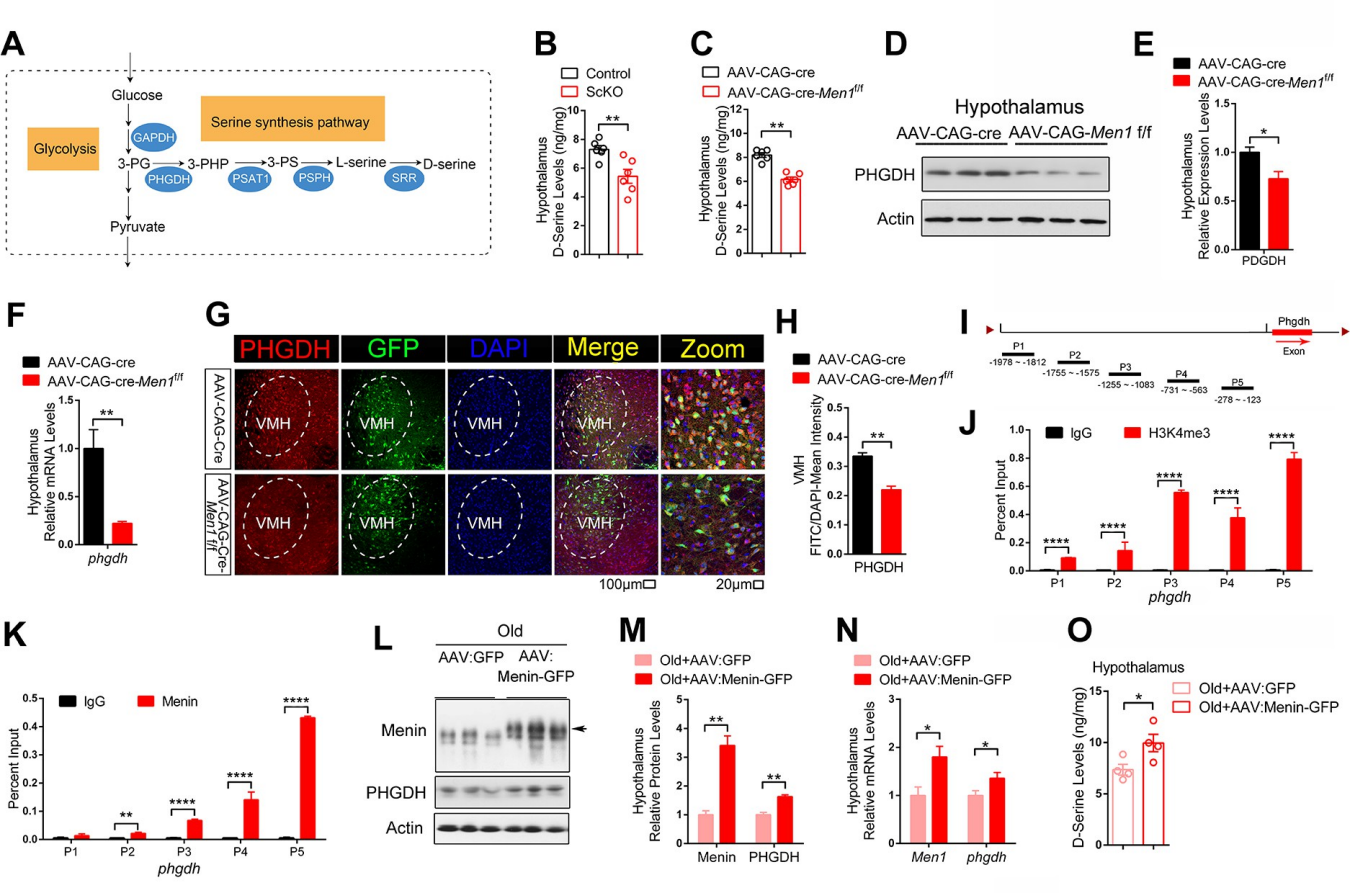
Menin deficiency results in disrupted D-serine synthesis in the hypothalamus. (A) Schematic diagram of D-serine synthesis pathway. (B, C) D-serine levels in lysates of the hypothalamus from 10 months male control and ScKO mice (B), and 10 months male AAV-CAG-Cre and AAV-CAG-Cre-*Men1*^f/f^ mice (C) were determined by ELISA; *n* = 6 mice. (D–F) The protein and mRNA levels of PHGDH in the hypothalamus from 10 months male AAV-CAG-Cre and AAV-CAG-Cre-*Men1*^f/f^ mice were measured; *n* = 3 mice or *n* = 6 mice, respectively. (G, H) Immunofluorescent staining of PHGDH (red) and GFP (green) in the hypothalamus region from 10 months male AAV-CAG-Cre and AAV-CAG-Cre-*Men1*^f/f^ mice. Representative confocal images are shown on panel G. Scale bar, 100 μm, 20 μm. Quantitation of fluorescence intensity of PHGDH are showed in panel H; *n* = 3 mice. (I) Schematic diagram of *phgdh* promoter region. (J, K) ChIP assays using antibodies against H3K4me3 or Menin were performed in cultured wild-type neurons on DIV 12; *n* = 3 independent experiments. (L–N) AAV-Menin-GFP virous or control virous AAV-GFP were injected into the hypothalamus region of 20 months male mice (Old+AAV:GFP; Old+AAV:Menin-GFP). The protein and mRNA levels of Menin and PHGDH were measured in the hypothalamus of above mice; *n* = 3 mice. (O) D-Serine levels in the hypothalamus from 20 months male Old+AAV:GFP and Old+AAV:Menin-GFP mice were determined by ELISA; *n* = 4 mice. Data represent mean ± SEM, n.s.: not significant, *p < 0.05, **p < 0.01, ***p < 0.001, one-way ANOVA with Tukey’s post hoc analysis. The underlying data of Fig 4 can be found in S4 Information. ChIP, chromatin immunoprecipitation; PHGDH, phosphoglycerate dehydrogenase.

De novo D-serine synthesis is catalyzed by phosphoglycerate dehydrogenase (PHGDH), phosphoserine aminotransferase (PSAT1), phosphoserine phosphatase (PSPH) [24], and serine racemase (SRR) (Fig 4A). It starts from conversion of 3-phosphoglycerate into 3-phosphohydroxypyruvate, catalyzed by PHGDH, the first rate-limiting enzyme of serine synthesis. Reduction in D-serine levels were further confirmed by measuring D-serine levels in the hypothalamus of ScKO mice and controls (Fig 4B), as well as in the hypothalamus of AAV-CAG-Cre-*Men1*^f/f^ and controls (Fig 4C). We then found that the expression of PHGDH, the first rate-limiting enzyme of serine synthesis, decreased significantly in VMH of AAV-CAG-Cre-*Men1*^f/f^ (Fig 4D–4H) and ScKO mice (S7 Fig) compared to controls. These data suggested that the decreased D-serine in Menin deletion condition is induced by the decline of PHGDH.

We next explored the mechanism underlying how Menin regulates expression of PHGDH. Menin contributes to epigenomic modulation of gene expression, partly through its association with H3K4me3 modification [25]. To test whether Menin regulates PHGDH through H3K4me3, we performed H3K4me3 chromatin immunoprecipitation (ChIP) assays by 5 distinct primer pairs targeting the *phgdh* promoter locus (Fig 4I) and found robust H3K4me3 binding in the *phgdh* promoter region from −1978 to −123 in primary neurons (Fig 4J). Furthermore, Menin-ChIP assays demonstrated Menin occupancy of the *phgdh* promoter region (Fig 4K). The ChIP PCR bands from above tests can be found in S8 Fig. We then overexpressed Menin in VMH by injecting Menin-AAV in 20-month-old mice (Fig 4L) and found the expression of PHGDH were significantly increased (Fig 4M and 4N). More importantly, the levels of D-serine in the hypothalamus of aged mice were also increased after overexpression of Menin (Fig 4O). These results suggest that Menin binds to *phgdh* promoter region and facilitates chromatin remodeling for *phgdh* transcription, which regulates D-serine synthesis.

### Restoring Menin in the hypothalamus ameliorates aging

**Fig 5:**
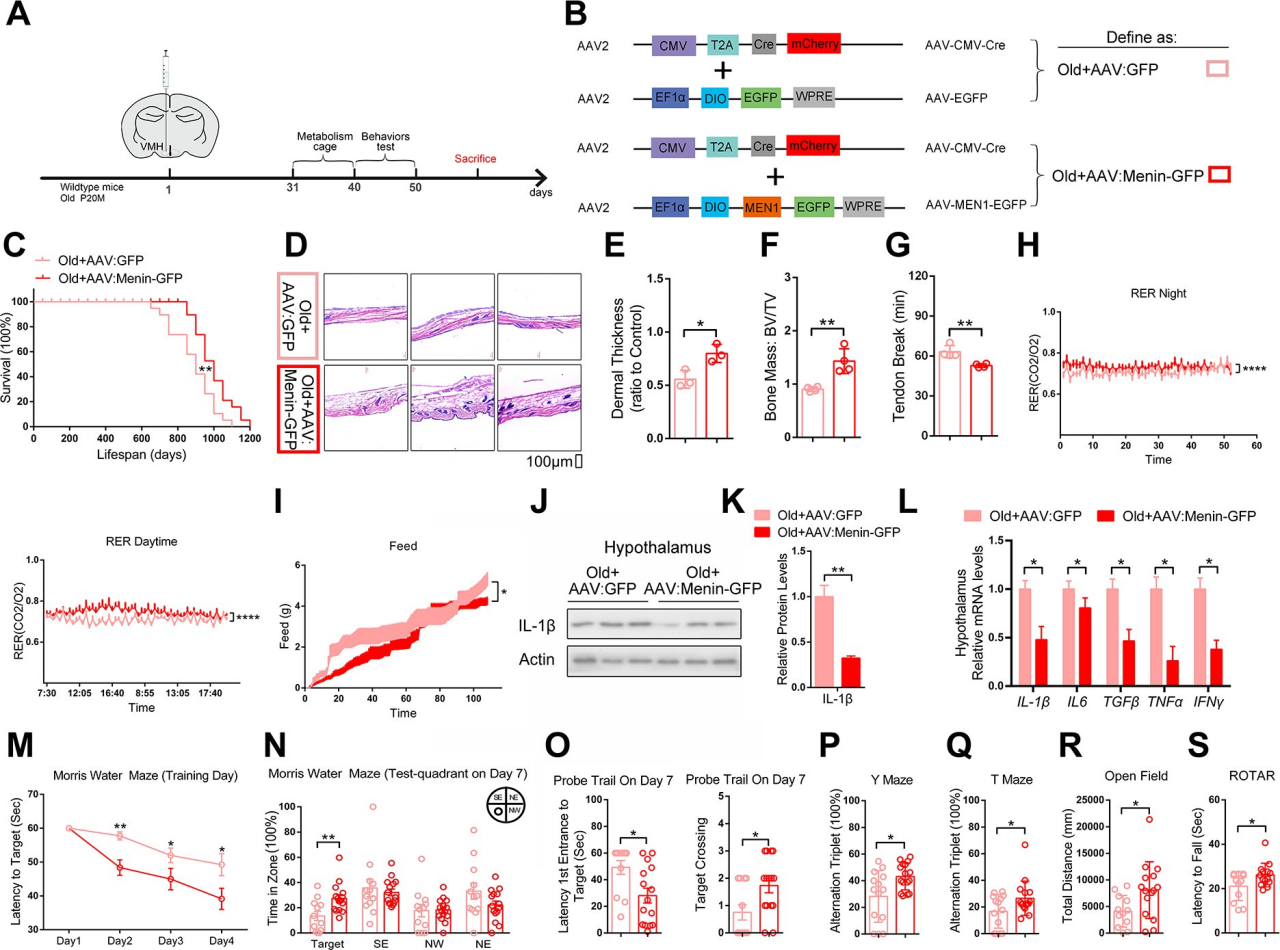
Enhanced Menin expression in the hypothalamus mice extends lifespan and ameliorates aging-related phenotype. (A, B) Detailed schematic diagram of overexpression of Menin by AAV in VMH of old male mice (20M). (C) Lifespan of these mice (*n* = 19 mice per group). (D–G) These mice were sacrificed for measuring dermal thickness (D, E), bone mass (F), and tail tendon breaking time (G); *n* = 4 mice. Scale bar, 100 μm. (H, I) The day and night respiratory quotients (RER) (H) and feed intakes (I) of 20 months male Old+AAV:GFP and Old+AAV:Menin-GFP mice were measured. (J–L) Inflammatory factors protein expression and mRNA levels in the hypothalamus of 20 months male Old+AAV:GFP and Old+AAV:Menin-GFP mice; *n* = 3 mice. (M–S) Behavioral analysis of 20 months male Old+AAV:GFP and Old+AAV:Menin-GFP mice by Morris water maze tests (M–O), Y maze (P), T maze (Q), open field (R), and rotarod test (S). Mouse number used in measuring energy expenditure by open circuit indirect calorimetry: Old+ AAV:GFP: *n* = 8 mice, Old+AAV:Menin-GFP: *n* = 8 mice. Mouse number used in behavior tests: Old+AAV:GFP: *n* = 14 mice, Old+AAV:Menin-GFP: *n* = 16 mice. Data represent mean ± SEM, n.s.: not significant, *p < 0.05, **p < 0.01, ***p < 0.001, Kaplan–Meier survival estimate for survival curve. Unpaired *t* test for behavioral statistics. Statistical applications between groups across multiple time points were analyzed by repeated-measures ANOVA. Other statistical applications were analyzed by one-way ANOVA with Tukey’s post hoc analysis. The underlying data of Fig 5 can be found in S5 Information. VMH, ventromedial hypothalamus.

We constructed a Cre recombinase-dependent Menin-AAV with EGFP expression and a CMV-cre AAV with mCherry expression. These 2 AAVs were combined and bilaterally injected into VMH of 20-month-old mice to restore Menin expression in VMH (Fig 5A and 5B). Overall, up-regulating Menin in VMH of 20-month-old mice could successfully increase mouse lifespan (Fig 5C) without significant changes in body weight, brain weight, and neuron number in hypothalamus and hippocampus (S9A–S9D Fig). Thirty days after AAV injection, Menin overexpression significantly reversed systemic aging phenotypes, including skin thickness, bone mass, and tail tendon collagen cross-linking (Fig 5D–5G). Notably, other aging-related phenotypes, such as inflammation levels, irregular metabolic circadian rhythm, and food intake were also significantly ameliorated by overexpression of Menin in VMH (Fig 5H–5L). Behavioral tests were subsequently performed 30 days after AAV injection. Notably, restoring Menin expression significantly improved the overall behavioral performance, including the impaired learning, cognition, and balance activity of old mice (Fig 5M–5S). Some of the old mice had a 0 score on T maze or Y maze test, suggesting that the low score might not only reflect aging-related cognitive decline but also aging-related decline of physical activity. To examine this possibility, we measured the free exploration distance in open field tests and found that the travel distance was significantly improved in old mice injected with AAV:Menin-GFP (VMH Menin overexpression) than old mice with AAV:GFP (control mice), but there was no significant difference in the swimming speed in the water maze between the 2 groups (S9E–S9G Fig).

### D-Serine supplement reduces cognitive decline in ScKO mice

**Fig 6:**
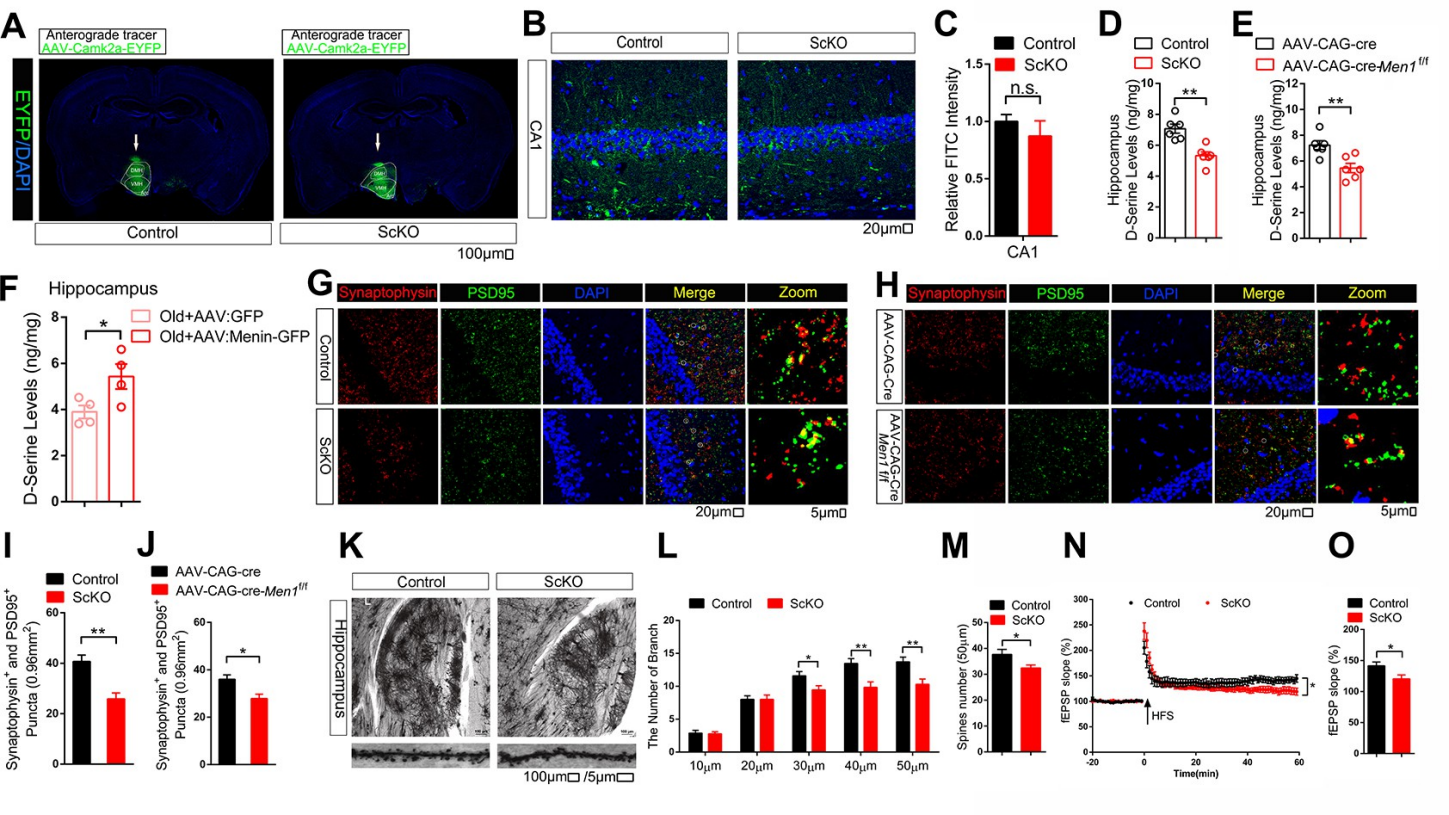
VMH Menin knockdown induced decreased hippocampal D-serine levels and impaired synaptic functions. (A) The schematic diagram of virus injection. AAV-CamkIIα-EYFP (200 nL) was injected into VMH of ScKO mice and control mice. Scale bar, 100 μm. (B, C) A representative EYFP-labeled fiber labeled in CA1 neuron. Representative images are shown in panel C. Scale bar, 20 μm. Quantitation of fluorescence intensity is shown in (C); *n* = 5 mice. (D–F) D-serine levels in hippocampus from 10 months male control and ScKO mice lysates (D), 10 months male AAV-CAG-Cre and AAV-CAG-Cre-*Men1*^f/f^ mice lysates (E), and Menin overexpressed in the hypothalamus region of old mice (F) were determined by ELISA; *n* = 4 or 6 mice. (G–J) Immunofluorescent staining of synaptophysin (red) and PSD95 (green) in hippocampus from 10 months male control and ScKO mice (G), and 10 months male AAV-CAG-Cre and AAV-CAG-Cre-*Men1*^f/f^ mice (H). Representative confocal images are shown on panel G and H, respectively. Scale bar, 20 μm, 5 μm. Quantitation of number of puncta of synaptophysin and PSD95 are showed in panel I and J, respectively, *n* = 6 slices from 3 mice. (K–M) Six months male ScKO mice and control mice were subjected to Golgi staining. Representative Golgi staining from cortex and hippocampal CA1 regions is shown in panel K. Scale bar, 100 μm, 5 μm; *n* = 3 mice. Quantitation of dendritic complexity in neurons from above mice was shown in panel L and M; *n* = 20 neurons. (N, O) LTP recordings from 6 months male ScKO and control mouse brain (*n* = 9 slices from 3 control mice; 8 slices from 4 ScKO mice). Data represent mean ± SEM, n.s.: not significant, *p < 0.05, **p < 0.01, ***p < 0.001, one-way ANOVA with Tukey’s post hoc analysis. The underlying data of Fig 6 can be found in S6 Information. LTP, long-term potentiation; VMH, ventromedial hypothalamus.

ScKO mice exhibited impaired cognition suggests that the neuronal function in hippocampus might has been dysregulated. We first examined the VMH-hippocampus neural projections and found there is no projection difference between control and ScKO mice (Fig 6A–6C). But the D-serine levels were significantly decreased in hippocampus of ScKO mice versus controls (Fig 6D), as well as in hippocampus of AAV-CAG-Cre-*Men1*^f/f^ versus controls (Fig 6E). Meanwhile overexpression of Menin in VMH significantly increased hippocampal D-serine levels in 20 months old mice (Fig 6F). Knockdown of Menin in VMH also reduced PSD95 and Synaptophysin expression in hippocampus (Fig 6G–6J). Golgi staining further indicates that the dendritic density of hippocampal neurons was also significantly reduced (Fig 6K–6M). Next, we performed an electrophysiological characterization of synaptic function in hippocampal CA1 region in control and ScKO mice. We observed a substantial reduction in high-frequency stimulation (HFS)-induced long-term potentiation (LTP) in ScKO mice (Fig 6N and 6O).

**Fig 7:**
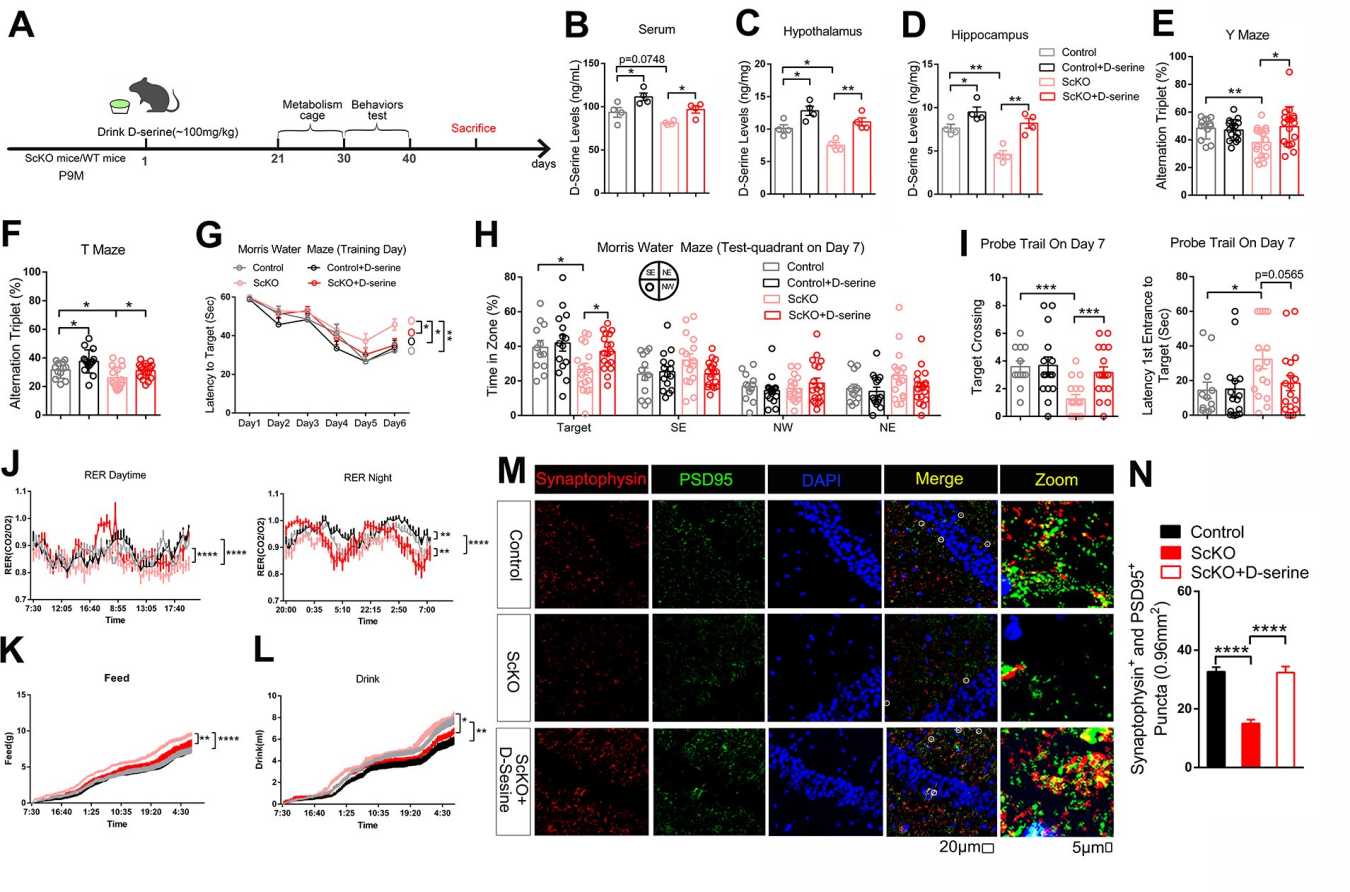
D-Serine supplement reduces cognitive decline in ScKO mice. (A) Schematic diagram of chronic oral D-serine supplementation (drinking water for 3 weeks). (B–D) D-serine levels in serum and lysates of hypothalamus and hippocampus from 10 months male control mice, control +D-serine mice, ScKO mice and ScKO+D-serine mice were determined by ELISA; *n* = 4 mice. (E–I) Behavioral tests of the above mice by Y maze (E), T maze (F), and Morris water maze tests (G–I). (J–L) The above mice were subjected to measure energy expenditure by open circuit indirect calorimetry. Day and night respiratory quotients (J), Exact dietary (K), and feed/water intake (L) were measured. (M, N) Immunofluorescent staining of synaptophysin (red) and PSD95 (green) in hippocampus from 10 months male control mice, ScKO mice, and ScKO+D-serine mice. Representative confocal images are shown on panel N. Scale bar, 20 μm, 5 μm. Quantitation of number of puncta of synaptophysin and PSD95 are showed in panel O, *n* = 6 slices from 3 mice. Mouse number used in behavior tests: Control: *n* = 12 mice, Control+ D-serine supplementation: *n* = 16 mice, ScKO: *n* = 15 mice, ScKO+ D-serine supplementation: *n* = 18 mice. Mouse number used in measuring energy expenditure by open circuit indirect calorimetry: Control: *n* = 8 mice, Control+ D-serine supplementation: *n* = 8 mice, ScKO: *n* = 8 mice, ScKO+ D-serine supplementation: *n* = 8 mice. Data represent mean ± SEM, n.s.: not significant, *p < 0.05, **p < 0.01, ***p < 0.001, ****p < 0.0001. Unpaired *t* test for behavioral statistics. Statistical applications between groups across multiple time points were analyzed by repeated-measures ANOVA. Other statistical applications were analyzed by one-way ANOVA with Tukey’s post hoc analysis. The underlying data of Fig 7 can be found in S7 Information.

We then wondered whether D-serine complement can attenuate the cognition decline in ScKO mice and old mice. Nine months old ScKO and control mice, 22 months old mice were given D-serine in water for 3 weeks (approximately 100 mg/kg body weight) [26] and then subjected to following tests, respectively (Figs 7A and S10A). The D-serine levels measured by ELISA indeed increased in serum, the hypothalamus and hippocampus of mice (Figs 7B–7D and S10B). Behavioral tests revealed that D-serine supplement rescued the cognitive deficits of 10-month-old ScKO mice or 22-month-old mice (Figs 7E–7I and S10E–S10I). The D-serine supplement also reversed metabolic disorders in ScKO mice (Fig 7J–7L) and hippocampus PSD95 and Synaptophysin expression levels in ScKO mice and old mice (Figs 7M and 7N, S10J, and S10K). These results suggest that D-serine reduction play a vital role in the cognition decline, and its complement can attenuate the cognition decline in ScKO mice and old mice.

## Discussion

The present study reported the important function of VMH Menin in regulating systemic aging. Manipulating Menin levels selectively in SF-1 neuron of VMH altered a variety of aging biomarkers in multiple systems and the aging process of whole body, through regulation of hypothalamic microinflammation and metabolic states (e.g., serine signaling). These results indicate Menin as a novel arbiter for systemic aging and the therapeutic potential for D-serine in treatment against cognitive decline.

The microinflammation of the hypothalamus leads to aging, which can be reversed by GnRH secreted by the pituitary gland [5]. The metabolic abnormalities of the body can also lead to microinflammation of the hypothalamus [8,9]. Menin is a powerful epigenetic regulation of gene transcription and cell signaling [27], affecting the secretion of multiple glands on the HPA axis and the metabolism of various organs in the body. At the same time, according to our previous findings [14], Menin could bind to the promoter region of p65 and inhibited its transcription, and the lost function variant of MEN1 (SNP rs375804228) failed to inhibit p65 transcription [14]. It is plausible that decreased Menin signaling contribute to the activated neuroinflammation in the hypothalamus.

In this article, we found that the expression of hypothalamic Menin decreases with age. The hypothalamic deficiency of Menin leads to an accelerated aging process, while the overexpression of Menin reverses systematic aging phenotype. Therefore, we speculate that the decline of Menin expression in the hypothalamus with age may be one of the driving factors of aging and Menin may be the key protein connecting the genetic, inflammatory, and metabolic factors of aging.

Metabonomics analysis and METARECON strategy indicated that D-serine biosynthesis pathway was significantly altered with Menin deficiency, suggesting that de novo D-serine biosynthesis in SF-1 neuron in VMH depends on Menin signaling. Previous research found that Menin is closely associated with glycometabolism and Menin inhibitors induced increase in glycolysis occurs in an mTOR-independent manner, which enhances the sensitivity of colorectal cancer cells to EGFR inhibitors [28]. Another study found that Menin regulates the D-serine biosynthetic pathway in Ewing sarcoma by inhibiting *phgdh* transcription [29], which is consistent with the findings in present study.

Previous studies identified that VMH SF-1 neurons act as important metabolic regulators [15–17] and play an important role in maintaining whole-body energy homeostasis. L-Serine is synthesized from 3-phosphohydroxypyruvate by a serial of reactions mediated by PSAT1 and PSPH [24,30]. L-serine is further converted to D-serine by D-serine synthase serine racemase (SRR). The main target of D-serine is N-methyl-D-aspartate receptor (NMDAR), which is essential for neurotransmission, learning, and memory formation, especially in CA1 and dentate gyrus of hippocampus [31–35]. Although several studies report that this L-serine/D-serine transformation also exit in astrocyte, recent new findings demonstrate that serine racemase is predominantly expressed by neuronal structures, suggesting that a significant fraction of D-serine comes from neurons [36–39]. The D-serine level is significantly decreased in conditional neuronal SRR-KO mice, suggesting the neuronal D-serine source. Interestingly, these mice also exhibit hippocampal LTP deficits and reduced NMDAR synaptic potentials [37,38]. On the other hand, conditional astrocytic SRR-KO mice display only a marginal decrease in brain serine racemase expression, with no changes in brain D-serine or NMDAR synaptic potentials [37,38]. These data suggested that serine racemase predominantly express in neurons [36,37,39]. Our data also suggested that D-serine may be a neuronal transmitter that is secreted by SF1 neurons.

Our data indicates that the remission of aging phenotype by D-serine is limited to cognitive improvement, leaving peripheral systems aging phenotypes unchanged. This may be due to the limited time of D-serine supplement, or there are other downstream pathways regulated by Menin that contributes to peripheral aging regulation.

By co-immunostaining Menin with SF-1, GFAP, and IBA1, we found that, with the increase of age, the expression of Menin decreased significantly only in SF-1 neurons, but not in astrocytes and microglia (S1M–S1R Fig). Therefore, the anti-aging effect of Menin in VMH is mainly mediated neuronal Menin. Hypothalamic Menin signaling diminished in neuron leads to increased levels of inflammation. This probably because the deficiency of Menin in neurons affects the interaction between neurons and glial cells in a certain way, and then leads to the release of cytokines from glial cells. We previously found that Menin can associate with p65 to inhibit NF-κB transactivation [14], inhibiting the neuroinflammation in astrocyte. Glial Menin may also play a role in inhibiting inflammation in VMH, which needs take the study forward in the further. Whether the up-regulation of cytokines is connected to D-serine biosynthesis also needs further studies.

By SF1-Cre mice, we deleted Menin at early stage of mice. The ScKO mice exhibited a premature aging phenotype in the middle of the age. To avoid the developmental adaptations of ScKO mice, we used viral-Cre approach to knockdown Menin in adult mice that also showed aging phenotype. Since the expression of Menin gradually decreases with age, earlier and longer knockout of Menin will lead aging appear earlier and severer.

For visualization the subcellular localization of Menin, we attached GFP with Menin when constructed AAV virus. The GFP fused Menin can up-regulate the transcription of *phgdh* and improve the synthesis of D-serine compared with GFP control. This data indicates that GFP-Menin functions as Menin alone. We are aware of the potential side effect of GFP fused protein expression, and the inclusion GFP may have potential effect on the normal expression, trafficking, and overall biological function in other ways. However, our data about GFP-Menin seems have no side effects.

Currently, the upstream regulators for Menin are unclear. Previous study showed that GLP-1 signaling-activated protein kinase A (PKA) directly phosphorylates Menin at the serine 487 residue, relieving Menin-mediated suppression of insulin expression and cell proliferation, while somatostatin stimulates Menin by suppression PKA [40,41].

In summary, we have identified the importance of VMH Menin in orchestration of aging pace. Menin levels may indicate the aging status and serve as anti-aging target.

## Materials and methods

### Human samples

Serum of different age groups of human subjects were acquired from the Department of Health Examination, the first affiliated hospital of Xiamen University. Study protocols received prior approval from the Ethics Committee of the first affiliated hospital of Xiamen University. The approval number of the protocol is XDYX202101K06. Informed content was written and obtained from all subjects. The samples were collected in adherence with the Declaration of Helsinki. Characteristic information can be found in Table 1.

**Table 1:** Characteristic information of different age groups of subjects.

Groups	Age (years)	Gender
Young individuals		
Subject 1	22	Male
Subject 2	26	Male
Subject 3	22	Male
Subject 4	25	Female
Subject 5	26	Female
Old individuals		
Subject 6	83	Female
Subject 7	83	Male
Subject 8	87	Female
Subject 9	87	Male
Subject 10	94	Male

### Animals

All mice were maintained within the laboratory animal center at Xiamen University, and all experimental procedures involved were performed according to protocols approved by the Institutional Animal Care and Use Committee at Xiamen University. The approval number of the protocol is XMULAC20200054. We also abide by the provisions of the Biosafety Law of the People’s Republic of China, the Regulations on the Administration of Experimental Animals, the National Standards for Experimental Animals (GB14925-2010), the Guidelines for Ethical Review of the Welfare of Experimental Animals (GBT 35892–2018), and the relevant rules and regulations formulated by Xiamen University. Mice were housed under a 12 h light/dark cycle with free access to standard rodent chow and water. Each cage housed a maximum of 4 mice. Mice were maintained under specific-pathogen-free (SPF) conditions and were not subject to immune suppression. Health of the animals used was regularly controlled by animal caretakers. All mice used were drug/test naïve. Six months male and female ScKO mice and control mice were used to test body weight, brain weight, Menin levels in CNS and peripheral tissues, and the hypothalamic inflammation levels. Six months male ScKO mice and control mice were used for Golgi staining and LTP recording. Male ScKO mice and control mice, male Old+AAV:GFP mice and Old+AAV:Menin-GFP mice were used to record lifespan. Ten months male and female ScKO mice and control mice were employed in behaviors tests. Ten months male ScKO mice and control mice were employed in open circuit indirect calorimetry, muscle fiber size, bone mass, skin thickness, myocardial thickness, β-Gal stain, and tail tendon collagen cross-linking tests. The hypothalamus of 13 months male ScKO and control mice were used for the RNA-seq tests and targeted metabolomics. Ten months male AAV-CAG-Cre mice and AAV-CAG Cre-*Men1*^f/f^ mice were employed in behaviors tests and open circuit indirect calorimetry. Twenty months male Old+AAV:GFP mice and Old+AAV:Menin-GFP mice were employed in behaviors tests, open circuit indirect calorimetry, bone mass, skin thickness, and tail tendon collagen cross-linking. Animals were used according to “3Rs” principles (Replacement, Reduction, and Refinement) in all experimental procedures.

The floxed *Men1* mouse strain (*Men1*^f/f^) was obtained from Dr. Guanghui Jin and Dr. Xianxin Hua [42]. SF1-Cre transgenic mice were bought from Jackson Laboratory. *Men1*-ScKO mice were obtained by crossing *Men1* floxed mice with respective SF1-Cre mouse lines. *Men1* floxed mice were used as controls.

### Experimental design

All experiments described in this study were performed a minimum of 3 biological reduplication. We did not use a statistical method to predetermine the proper sample size. The sample size per experiment was determined according to previous publications.

### Open circuit indirect calorimetry

The optimum indirect calorimetry system (Sable Promethion) was used for simultaneous measurements of mice undergoing dietary studies at same time. Systems of up to 16 cages were available for such simultaneous measurements. The mice were reared in single cage for 2 days before recording. After that, the mice were put into the calorimetry system to record the energy metabolism of a week. The day-night shift occur in the animal house were 08:00 and 20:00. VO_2_, VCO_2_, energy expenditure, respiratory quotient, mass of food consumed, mass of water consumed, body weight, and distance in locomotion were recorded.

### ChIP

ChIP procedures were performed according to the manufacturer’s instructions (Millipore, 17–295), which have been detailed descripted in the method of our previous study [25].

### Generation of AAVs and stereotaxic injection

pAAV-CAG-cre-GFP (virus titer: 5.23 × 10^12^/mL) were purchased from BrainVTA (Wuhan, China). AAV-CMV-Cre-mCherry (virus titer: 5.02 × 10^12^/mL) were purchased from OBiO Technology Corperation (Shanghai, China). AAV-EF1α-DIO-MEN1-EGFP-WPRE (virus titer: 5.05 × 10^12^/mL) and AAV-EF1α-DIO-EGFP-WPRE (virus titer: 5.62 × 10^12^/mL) were purchased from BrainVTA (Wuhan, China). We attached GFP with Menin in a classical fusion to visualize the subcellular overexpression of Menin when constructed AAV virus. Packaged viruses were stereotactically injected into the VMH of control mice or *men1*^f/f^ mice respectively as described previously. All mice were anesthetized and placed in a stereotaxic frame, a skin incision was made and holes were drilled at VMH (x(±0.4 mm from bregma) and y(−1.58 mm from bregma)). A total of 0.2 μl viruses were delivered at 0.20 μl/min at z-depths of 5.7 mm. The syringe was left in place for 10 min after each injection, before being withdrawn slowly. rAAV-CaMKIIα-EYFP-WPRE-pA (virus titer: 3.26 × 10^12^/mL) were purchased from Brain VTA (Wuhan, China). To investigate the projection from the VMH toward to the hippocampus, anterograde virus tracer AAV CaMKIIα-EYFP was stereotactically injected into the VMH in ScKO and control mice, respectively. The mice were allowed to survive for 3 weeks and then to observe EYFP-labeled fibers within the hippocampus.

### Electrophysiology

For hippocampal slice LTP recordings procedure, please refer to the method of our previous study [14].

Whole-cell patch clamp recordings were obtained from SF-1 neurons in the hypothalamus of ScKO mice and control mice using 4 to 8 MΩ borosilicate glass pipettes (Harvard Apparatus). The pipette recording solution contained (in mmol 1−1): 8.0 NaCl, nominally 0.0001 CaCl_2_, 0.3 Na-GTP, 130 potassium gluconate, 10.0 Na-Hepes, 1.0 MgCl_2_, and 2.0 Na-ATP (pH adjusted to 7.4 with methanesulfonic acid; 295 to 300 mosmol 1^−1^). The pipettes with an Ag–AgCl electrode were connected to a CV-4 head stage and an Axopatch-1D amplifier with a Digi data 1200 interface (Axon Instruments). The pipettes are positioned within the tissue using a motorized patch-clamp micromanipulator. Seal resistance was typically 4 to 10 GΩ. Typical whole-cell access resistance (R_a_) was 5 to 30 MΩ and whole-cell leak was below 20 pA [43]. Miniature excitatory postsynaptic currents (mEPSCs) and miniature inhibitory postsynaptic currents (mIPSCs) were recorded in the presence of tetrodotoxin (500 nmol/L). We recorded mEPSCs and mIPSCs at holding potentials of –70 and 0 mV, respectively, in the same cell (3 min each; *n* > 20 cells/group).

### Supplementation of D-serine

ScKO and control mice were given D-serine (600 mg/L, Sigma-Aldrich, St. Louis, Missouri, United States of America) in the drinking water for 3 weeks. Each mouse took about 5 mL per day.

### RNA-sequencing analysis

The hypothalamus of ScKO and control mice were harvested. Isolated RNA was obtained for RNA-sequencing analysis [14]. cDNA library construction and sequencing were performed on Illumina platform by Beijing Novogene Corporation. High-quality reads were aligned to the mouse reference genome using Bowtie 2. Expression levels for each of the genes were normalized to fragments per kilobase of exon model per million mapped reads (FPKM) using RNA-seq by Expectation Maximization (RSEM). Genes with ≥2-fold change and *p* < 0.05 were considered to be statistically significant. DEGs between samples were identified and clustering analysis. Then, functional annotations were performed.

### ELISA

D-Serine levels in mouse serum, hippocampus, and hypothalamus were measured using ELISA Assay Kits (D-serine Elisa Kit, CAT CZY-041905M, Nanjing Caobenyuan Biotech Co.) according to the manufacturer’s instructions.

### Primary neuron culture procedures

Primary neurons were dissected from timed-pregnant females at E16.5. Briefly, brain cortices were dissected from pups of varying genotypes. The Meninges were removed, and cortical tissue was dissociated by enzymatic digestion. Isolated primary neurons were plated on poly-D-lysine–coated dishes and cultured in Neurobasal medium supplemented with B27 (Gibco)/1% penicillin/streptomycin (Invitrogen) and maintained in a 5% CO_2_ incubator at 37°C.

### Global DNA methylation assay

DNA methylation levels in mouse hypothalamus were measured using Global DNA Methylation Assay Kit (Global DNA Methylation Assay Kit, ab233486, Abcam) according to the manufacturer’s instructions.

### Senescence-associated β-galactosidase assay with immunostaining

Senescence β-galactosidase staining was performed using a colorimetric kit (Abcam) according to the manufacturer’s protocol.

### Behavioral studies

For all the behavioral tests procedures (T and Y maze, forced swimming test, tail suspension test, sucrose preference test (SPT), sucrose consumption test (SCT), rotarod test, open field test, and Morris water maze), please refer to the methods in our previous study [14].

### Western blotting

Cultured cells and mouse brain tissues (include hypothalamus and hippocampus) were homogenized in lysis buffer (RIPA) on ice for 40 min and subsequently centrifuged at 12,000 rpm for 10 min at 4°C. Supernatants were transferred to a clean 1.5 mL tube, and protein samples were resolved by SDS-PAGE (sodium dodecyl sulfate–polyacrylamide gel electrophoresis) and subsequent immunoblotted onto polyvinylidene difluoride (PVDF) membranes. Sample containing 30 μg of protein was separated using 10% SDS-PAGE gels. Proteins were transferred onto PVDF membranes in an ice-cold buffer (25 mM Tris-HCl, 192 mM glycine, and 20% methanol) by electro transfer for 1.5 h. Immunoblots were probed with indicated antibodies. Goat-anti-mouse secondary antibodies and goat-anti-rabbit secondary antibody were purchased from Millipore (#AP132P, #AP124P). Quantification of band intensities were normalized to β-actin, and averaged from at least 3 independent experiments.

### Immunofluorescence

Mouse brain sections or cultured cells were washed 3 times with PBS and antigen retrieval was performed using citrate buffer (pH 7.0); samples were then permeabilized and blocked in PBS containing 0.5% Triton X-100 and 10% normal goat serum at room temperature for 1 h. Sections were incubated with primary antibodies in blocking buffer overnight at 4°C. After washing, secondary antibodies were added to the blocking buffer and incubated for 1 h. Samples were then washed and counterstained with DAPI. Images were acquired using a Nikon confocal microscope.

Primary antibodies used for immunostaining include: Menin (rabbit, 1:1,000; Abcam, Ab4452; mouse, 1:1,000; Thermo Fisher, A500-0003A), PHGDH (rabbit, 1:300; Life Span, #LS-C81937-50), PSAT1 (rabbit, 1:100; Life Span, #LS-C80875-50), PSPH (rabbit, 1:100; lifespan, #LS-C38237-50), SF1(rabbit,1:500; Abcam, Ab168380), GFAP (mouse,1:500; Cell Signaling Technology, #3670), NeuN (mouse, 1:1,000; Millipore, #MAB377), Iba1 (goat, 1:200; Abcam, Ab48004), 488/594 donkey anti-mouse/rabbit secondary antibodies (1:500), and Mounting Medium with DAPI was purchased from Invitrogen.

### Quantitative RT-PCR

Total RNA from animal tissues and cells were isolated using Trizol reagent according to the manufacturer’s instructions (Invitrogen). Reverse transcription was performed using ReverTra Ace qPCR RT Master Mix (Toyobo, FSQ-201). RNA concentrations were adjusted to 2 g/L in nuclease-free water, and total RNA was reverse-transcribed in a 20 μL reaction volume. cDNA was amplified by real-time quantitative RT-PCR using SYBR Green (Roche) reagent. Samples were assayed in triplicate and actin was used as an internal control. Primer sequences used in this study can be found in Table 2.

**Table 2:** Primer sequences used in this study.

Gene	Primer sequences
Menin	Forward 5′-TGCCATGCGGCTCTCTGATG-3′
	Reverse 5′-CTTGACGGAGGCCGTTGGGTT-3′
PHGDH	Forward 5′- ATGGCCTTCGCAAATCTGC-3′
	Reverse 5′- AGTTCAGCTATCAGCTCCTCC-3′
PSAT	Forward 5′- CAGTGGAGCGCCAGAATAGAA-3′
	Reverse 5′- CCTGTGCCCCTTCAAGGAG -3′
PSPH	Forward 5′- AGGAAGCTCTTCTGTTCAGCG-3′
	Reverse 5′- GAGCCTCTGGACTTGATCCC-3′
SSR	Forward 5′-ACCCCAGTGCTAACAAGCTC-3′
	Reverse 5′-CACCTCGAATCTTAAAAGACCCA-3′
β-actin	Forward 5′-AGTGTGACGTTGACATCCGTA-3′
	Reverse 5′-GCCAGAGCAGTAATCTCCTTC-3′

### Targeted metabolomics

#### Sample preparation

The hypothalamus of ScKO and control mice were weighed before the extraction of metabolites and dried lyophilized were ground in a 2 mL Eppendorf tube containing a 5-mm tungsten bead for 1 min at 65 Hz in a Grinding Mill. Metabolites were extracted using 1 mL precooled mixtures of methanol, acetonitrile, and water (v/v/v, 2:2:1) and then placed for 1 h ultrasonic shaking in ice baths. Subsequently, the mixture was placed at −20°C for 1 h and centrifuged at 14,000 g for 20 min at 4°C. The supernatants were recovered and concentrated to dryness in vacuum.

#### UHPLC-MS analysis

The LC/MS portion of the platform was based on a Thermo Fisher Vanquish UHPLC equipped with an ACQUITY UPLC BEH Amide column (1.7 μm, 2.1 mm × 100 mm, Wasters) and a Thermo-TSQ Vantage mass spectrometer. Energy metabolites were monitored in electrospray negative-ionization and positive-ionization mode. The 2 μL samples were injected sequentially into a Thermo-TSQ Vantage mass spectrometer equipped with a Vanquish UHPLC system with autosampler (Thermo Fisher). The ACQUITY UPLC BEH Amide column (1.7 μm, 2.1 mm × 100 mm, Wasters) was heated to 45°C under a flow rate of 300 μL/min. A gradient was used to separate the compounds consisted of 20 mM ammonium acetate (solvent A) and 5% acetonitrile (solvent B). The gradient started at 5% solvent A for 1 min and increasing linearly to 35% solvent A over 13 min, and then increasing linearly to 60% solvent A over 2 min with a 2-min hold before returning the starting mixture during 0.1 min and re-equilibrating for 4 min. QC samples were injected every 6 or 8 samples during acquisition.

The MS conditions were as follows: Collision Gas Pressure (mTorr): 1.0; Q1 Peak Width (FWHM): 0.70; Q3 Peak Width (FWHM): 0.70; Cycle Time (s): 1.500; Capillary Temperature: 350.0°C; Vaporizer Temperature: 350.0°C; Sheath Gas Pressure: 35.0; Aux Valve Flow: 10.0; Spray Voltage: Positive polarity −3,500.0V; Negative polarity −3,000.0V; scan type: selected reaction monitoring/multiple reaction monitoring (SRM/MRM).

#### Data preprocessing and filtering

Raw MRM data files were processed by peak finding, alignment, and filtering using Xcalibur Qual browser software.

#### Multivariate statistical analysis

Simcap 14 software (Umetrics, Umeå, Sweden) was used for all multivariate data analyses and modeling. Data were mean-centered using Pareto scaling. Models were built on principal component analysis (PCA), orthogonal partial least-square discriminant analysis (OPLS-DA), and partial least-square discriminant analysis (PLS-DA). All the models evaluated were tested for over fitting with methods of permutation tests. The descriptive performance of the models was determined by R2X (cumulative) (perfect model: R2X (cum) = 1) and R2Y (cumulative) (perfect model: R2Y (cum) = 1) values while their prediction performance was measured by Q2 (cumulative) (perfect model: Q2 (cum) = 1) and a permutation test (*n* = 200). The permuted model should not be able to predict classes: R2 and Q2 values at the Y-axis intercept must be lower than those of Q2 and the R2 of the non-permuted model. OPLS-DA allowed the determination of discriminating metabolites using the variable importance on projection (VIP). The VIP score value indicates the contribution of a variable to the discrimination between all the classes of samples. Mathematically, these scores are calculated for each variable as a weighted sum of squares of PLS weights. The mean VIP value is one, and usually VIP values over one are considered as significant. A high score is in agreement with a strong discriminatory ability and thus constitutes a criterion for the selection of biomarkers.

The discriminating metabolites were obtained using a statistically significant threshold of variable influence on projection (VIP) values obtained from the OPLS-DA model and two-tailed Student’s *t* test (*p* value) on the normalized raw data at univariate analysis level. The *p* value was calculated by one-way analysis of variance (ANOVA) for multi-group analysis. Metabolites with VIP values greater than 1.0 and *p* value less than 0.05 were deemed to be statistically significant metabolites. Fold change was calculated as the logarithm of the average mass response (area) ratio between 2 arbitrary classes. On the other side, the identified differential metabolites were used to perform heatmap analyses with R package.

### METARECON strategy

To identify a specific metabolic pathway which affected by Menin deletion in VMH, we apply a new method termed METARECON. The functional integration of GC-MS metabolomics data into a biochemical metabolic network structure was performed by the inverse approximation of the biochemical Jacobian matrix. This approximation directly connects the covariance matrix (COV), which was built from the experimental metabolomics data, to the metabolic network structure of the primary metabolism. The metabolic network model is provided in S6A Fig.

Firstly, an appropriate simulation condition and data structure are made. The generic type of 1 is known as the “Lyapunov Equation,” which is widely applied to control systems.

$$JAC*COV+COV*{JAC}^{T}=-2FLU$$ (1)

where JAC represents the Jacobian matrix, the COV matrix is derived from the biological variance of independent replication analysis between a set of samples, and FLU is a fluctuation matrix that integrates a Gaussian noise function simulating metabolic fluctuations under steady-state conditions. Jacobian matrix JAC is calculated from diffusion matrix FLU and covariance matrix COV constructed from metabonomic data; 1 not only considers the noise in the data captured by the fluctuation matrix, but also combines the statistical characteristics of the data with the dynamic characteristics of a stable system.

Then, the metabolism pathway efficiency consistent is made. In a biochemical environment, *r* represents the rates for each reaction and *C* represents metabolite concentration changes, each element of JAC means the elasticity of reaction rates to any change of metabolite concentrations. The corresponding Jacobian is a matrix of all first-order partial derivatives of all functions *r*_*i*_ on all metabolites *C*_*j*_, as shown in Eq 2. The Jacobian matrix JAC in 1 is defined as

$$JAC={\left[\begin{array}{ccc} \frac{\partial r_{1}}{\partial C_{1}} & \frac{\partial r_{1}}{\partial C_{2}}\;\cdots & \frac{\partial r_{1}}{\partial C_{n}}\\ \frac{\partial r_{2}}{\partial C_{1}} & \frac{\partial r_{2}}{\partial C_{2}}\;\cdots & \frac{\partial r_{2}}{\partial C_{n}}\\ \vdots & \ddots & \vdots \\ \frac{\partial r_{n}}{\partial C_{1}} & \frac{\partial r_{n}}{\partial C_{2}}\cdots & \frac{\partial r_{n}}{\partial C_{n}} \end{array}\right]}_{n\times n}$$ (2)

The next step is to consolidate dynamic equation of biological reaction. In this algorithm, Jacobian matrix is used to describe the local dynamic characteristics near the steady state of the system.

A set of differential equations represent the dynamics of metabolic pathways composed of multiple metabolites. As shown in below, where reaction rates *r*(*r*_1_, *r*_2_,⋯*r*_*n*_) are the functions of metabolite concentrations *C*(*C*_1_, *C*_2_,⋯*C*_*n*_) over time.


$$r_{i}(C_{1},C_{2},{\cdots C}_{n})=\frac{dC_{i}}{dt}$$ (3)

By this means, as described in Eq 4, the matrix describes the change of each metabolite and its impact on the change of other metabolites.


$${\left[\begin{array}{ccc} COV(C_{1},C_{1}) & COV(C_{1},C_{2})\cdots & COV(C_{1},C_{n})\\ COV(C_{2},C_{1}) & COV(C_{2},C_{2})\cdots & COV(C_{2},C_{n})\\ \vdots & \ddots & \vdots \\ COV(C_{n},C_{1}) & COV(C_{n},C_{2})\cdots & COV(C_{n},C_{n}) \end{array}\right]}_{n\times n}$$ (4)

The METARECON approach links the kinetic equation of the metabolic pathway represented by above to the covariance of the relevant metabolite concentration data represented by Eq 4. The above methods are combined to further substitute the data into Eq 2. This includes the integration of precursor synthesis, substrate utilization, and energy conversion pathways in biological metabolism.

The last step is model improvement through metabolic interaction reannotation. JAC matrix has more independent variables than symmetric covariance matrix, so parametric solution is needed to eliminate the uncertainty. By quoting RENEW to determine the linear system, the solution of Jacobian matrix is obtained. RENEW is a metabolic interaction matrix, also known as a stoichiometric matrix, which represents the interdependence of metabolic flux and metabolites. In fact, there exists regulation between metabolites without substances consumption. Then, we can determine the non-zero in the Jacobian matrix by importing the stoichiometric matrix of the metabolic network, and point out potential reaction in the basic biochemical network for the final analysis. Based on the above theorem, we give a strict transformation from Eqs 2–5.


$$JAC=RENEW\frac{\partial r}{\partial C}$$ (5)

### Statistical analysis

All data presented are expressed as arithmetic mean ± SEM. All statistical analyses were performed using GraphPad Prism version 8.0. Null hypotheses were rejected at *p* values equal to or higher than 0.05. Kaplan–Meier survival estimate was used for survival curve. We conducted a paired *t* test on open circuit calorimetry data of 2 groups. Unpaired *t* test was applied to 2 groups of behavioral statistics. For statistical comparisons between 2 groups, we first performed a Shapiro–Wilk normality test (Prism) to determine whether the data was likely normally distributed. Statistically significant differences between groups were determined using one-way ANOVA. In evaluating multiple comparisons, Bonferroni methods were used to adjust *p* values accordingly to lower the probability of type I errors.

All electrophysiological results were analyzed using Sigma Stat 4 statistical software. Statistical significance was evaluated by one-way ANOVA with Holm–Sidak pair-wise tests. Values of *p* < 0.05 were considered statistically significant. DNASTAR Laser gene software (version 7.1) was used to analyze Sanger sequencing data.

## Supporting information

S1 Fig The expression of Menin in different brain regions and different cell types of young and old mice, related to Fig 1. (TIF)

S2 Fig Physiological characteristics of ScKO mice, related to Fig 1. (TIF)

S3 Fig 10M male ScKO mice showed no other phenotypes besides cognition impairments, and 10M female ScKO mice also showed cognition impairments phenotypes, related to Fig 1. (TIF)

S4 Fig Menin is also knocked-down in other SF1-expressing glands of ScKO mice, related to Fig 2. (TIF)

S5 Fig Physiological characteristics of AAV-CAG-cre and AAV-CAG-cre-Men1^f/f^ mice, related to Fig 3. (TIF)

S6 Fig Targeted metabolomics analysis of the hypothalamus of 13M ScKO and control mice, related to Fig 3. (TIF)

S7 Fig The expression of PHGDH is inhibited in ScKO mice, related to Fig 4. (TIF)

S8 Fig Representative gel band images of ChIP assays, related to Fig 4. (TIF)

S9 Fig Physiological characteristics of Old+AAV:GFP and Old+AAV:Menin-GFP mice, related to Fig 5. (TIF)

S10 Fig D-Serine supplement reduces cognitive decline in old mice, related to Fig 6. (TIF)

S1 Dataset The differential expressed genes (DEGs) in the hypothalamus of 13 months male control and ScKO mice. (XLSX)

S1 Raw images All the original, uncropped images supporting the blots reported in the study with the loading order, identity of experimental samples, method used to capture the image. (PDF)

S1 Information The underlying data of Fig 1 can be found in S1 Information. (XLSX)

S2 Information The underlying data of Fig 2 can be found in S2 Information. (XLSX)

S3 Information The underlying data of Fig 3 can be found in S3 Information. (XLSX)

S4 Information The underlying data of Fig 4 can be found in S4 Information. (XLSX)

S5 Information The underlying data of Fig 5 can be found in S5 Information. (XLSX)

S6 Information The underlying data of Fig 6 can be found in S6 Information. (XLSX)

S7 Information The underlying data of Fig 7 can be found in S7 Information. (XLSX)

S1 Data The underlying data of S1 Fig can be found in S1 Data. (XLSX)

S2 Data The underlying data of S2 Fig can be found in S2 Data. (XLSX)

S3 Data The underlying data of S3 Fig can be found in S3 Data. (XLSX)

S4 Data The underlying data of S4 Fig can be found in S4 Data. (XLSX)

S5 Data The underlying data of S5 Fig can be found in S5 Data. (XLSX)

S6 Data The underlying data of S6 Fig can be found in S6 Data. (XLSX)

S7 Data The underlying data of S7 Fig can be found in S7 Data. (XLSX)

S8 Data The underlying data of S9 Fig can be found in S8 Data. (XLSX)

S9 Data The underlying data of S10 Fig can be found in S9 Data. (XLSX)

## Abbreviations

ChIP: chromatin immunoprecipitation
DAMP: damage-associated molecular pattern
DEG: differentially expressed gene
GnRH: gonadotropin-releasing hormone
HFS: high-frequency stimulation
LPS: lipopolysaccharide
LTP: long-term potentiation
mEPSC: miniature excitatory postsynaptic current
mIPSC: miniature inhibitory postsynaptic current
MRM: multiple reaction monitoring
OPLS-DA: orthogonal partial least-square discriminant analysis
PCA: principal component analysis
PHGDH: phosphoglycerate dehydrogenase
PKA: protein kinase A
PLS-DA: partial least-square discriminant analysis
PRR: pattern recognition receptor
PSPH: phosphoserine phosphatase
PVDF: polyvinylidene difluoride
SCT: sucrose consumption test
SFA: saturated fatty acid
SF-1: steroidogenic factor-1
SPF: specific-pathogen-free
SPT: sucrose preference test
SRM: selected reaction monitoring
TLR4: Toll-like receptor 4
TNF: tumor necrosis factor
VIP: variable importance on projection
VMH: ventromedial hypothalamus
