# Supplementary figures and images for: Hypothalamic Menin regulates systemic aging and cognitive decline
Source: PLoS Biol. 2023 Mar 16;21(3):e3002033. doi: 10.1371/journal.pbio.3002033 (PMC10019680; doi:10.1371/journal.pbio.3002033)

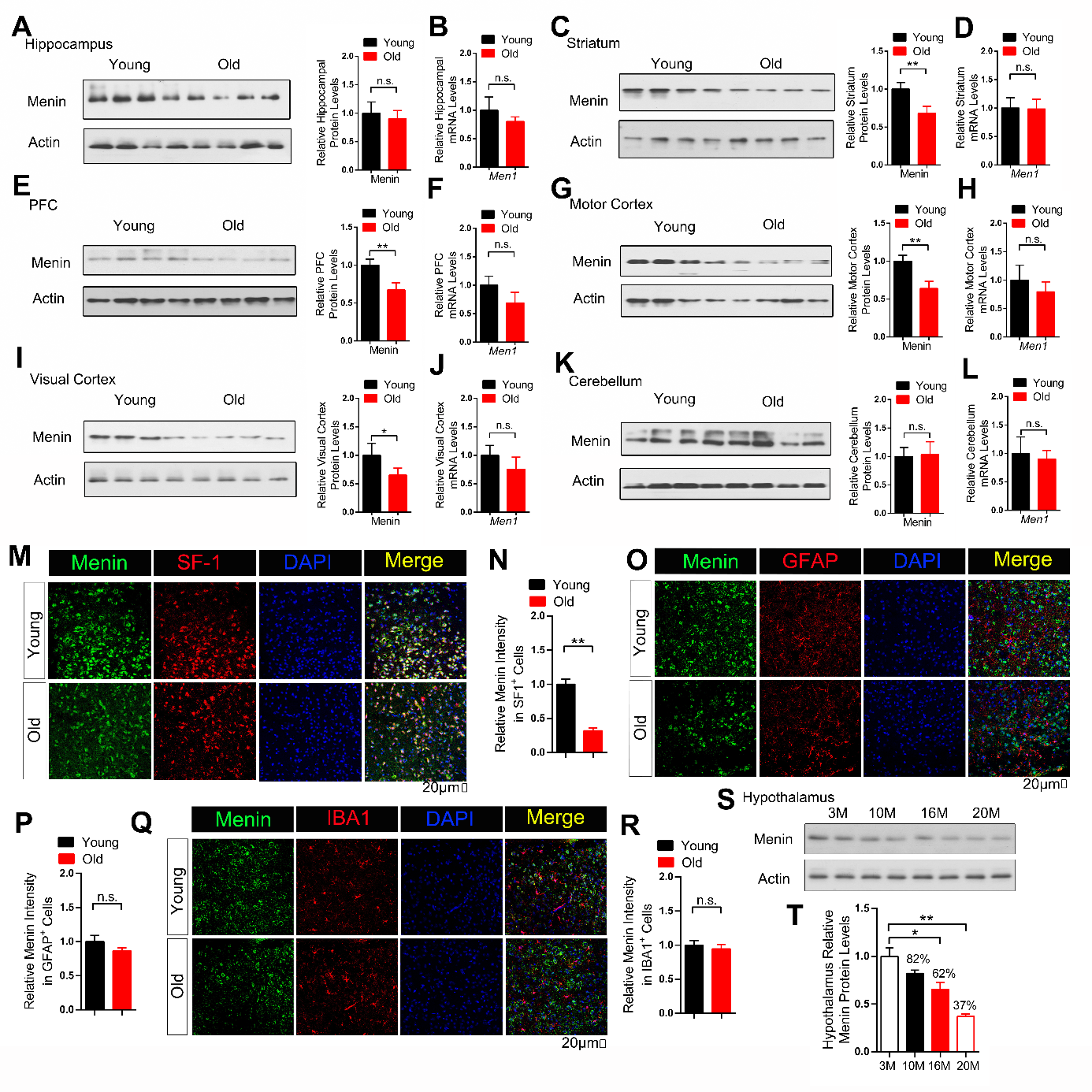

Supplement: S1 Fig — (TIF) [file pbio.3002033.s001.tif]

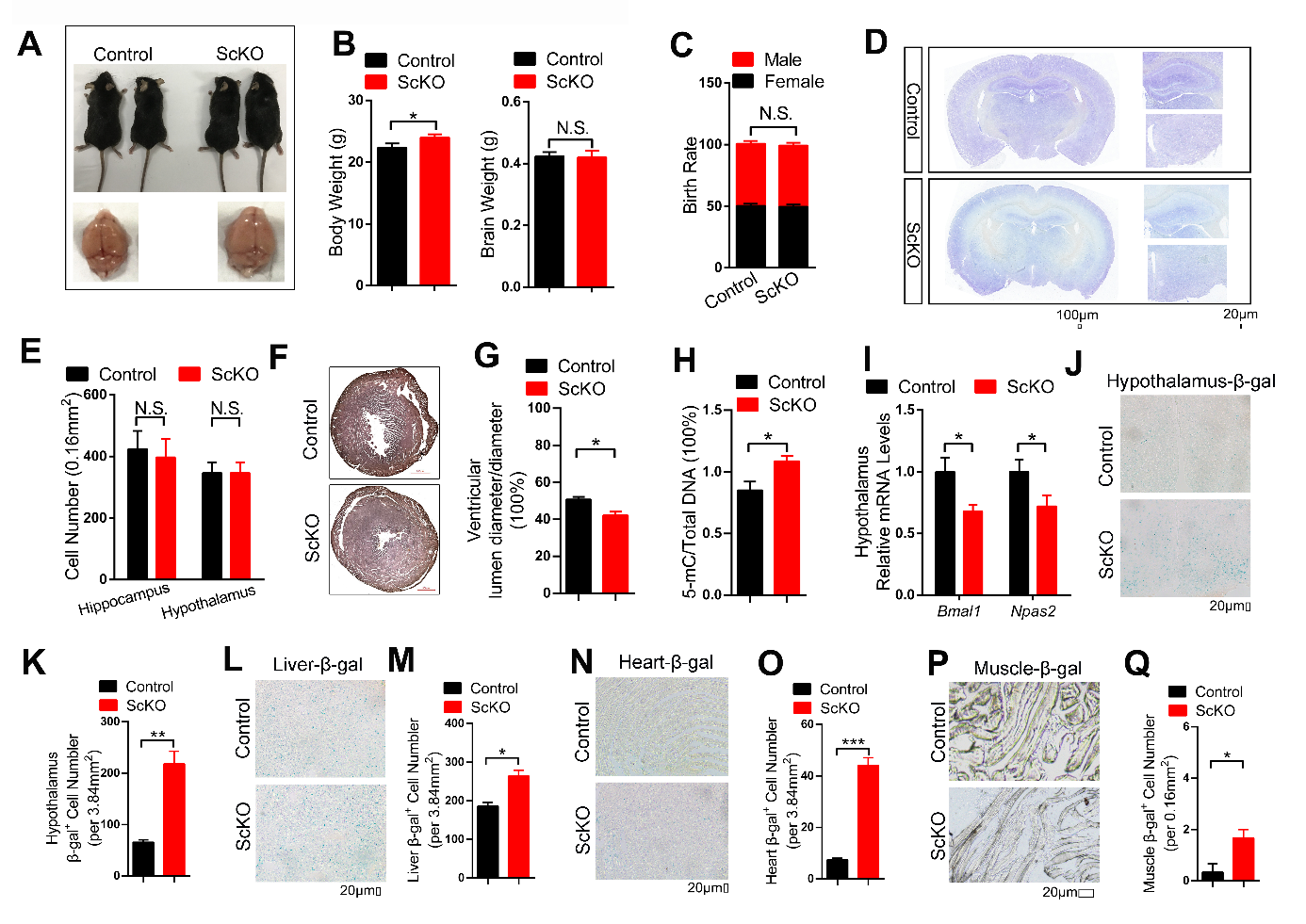

Supplement: S2 Fig — (TIF) [file pbio.3002033.s002.tif]

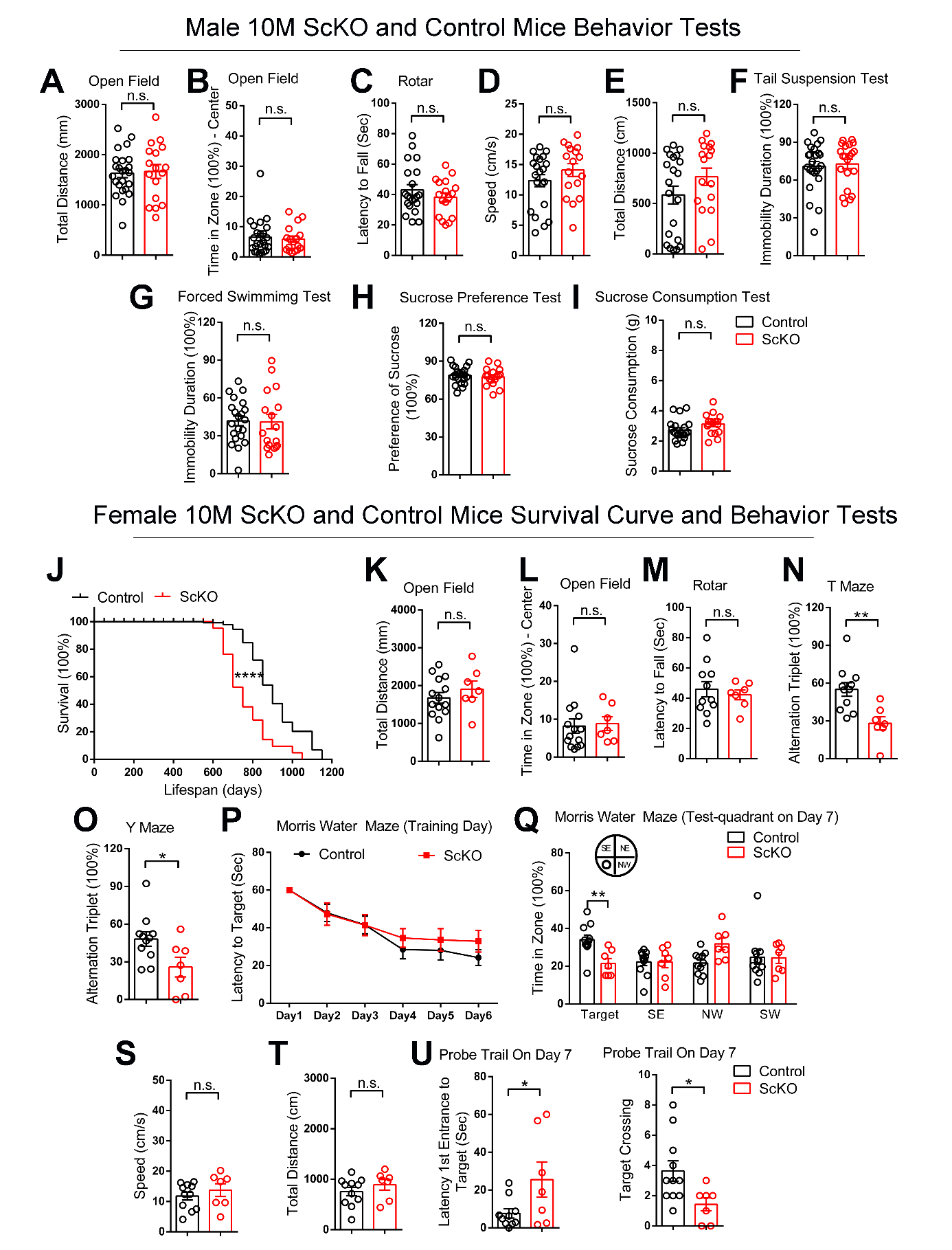

Supplement: S3 Fig — (TIF) [file pbio.3002033.s003.tif]

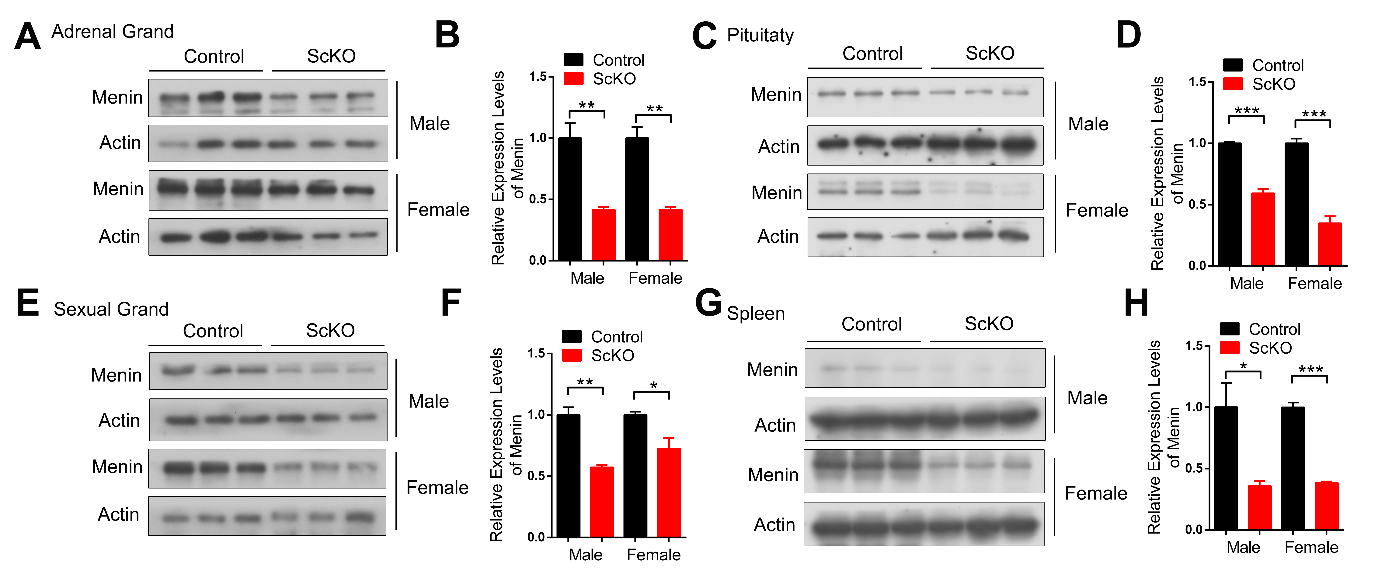

Supplement: S4 Fig — (TIF) [file pbio.3002033.s004.tif]

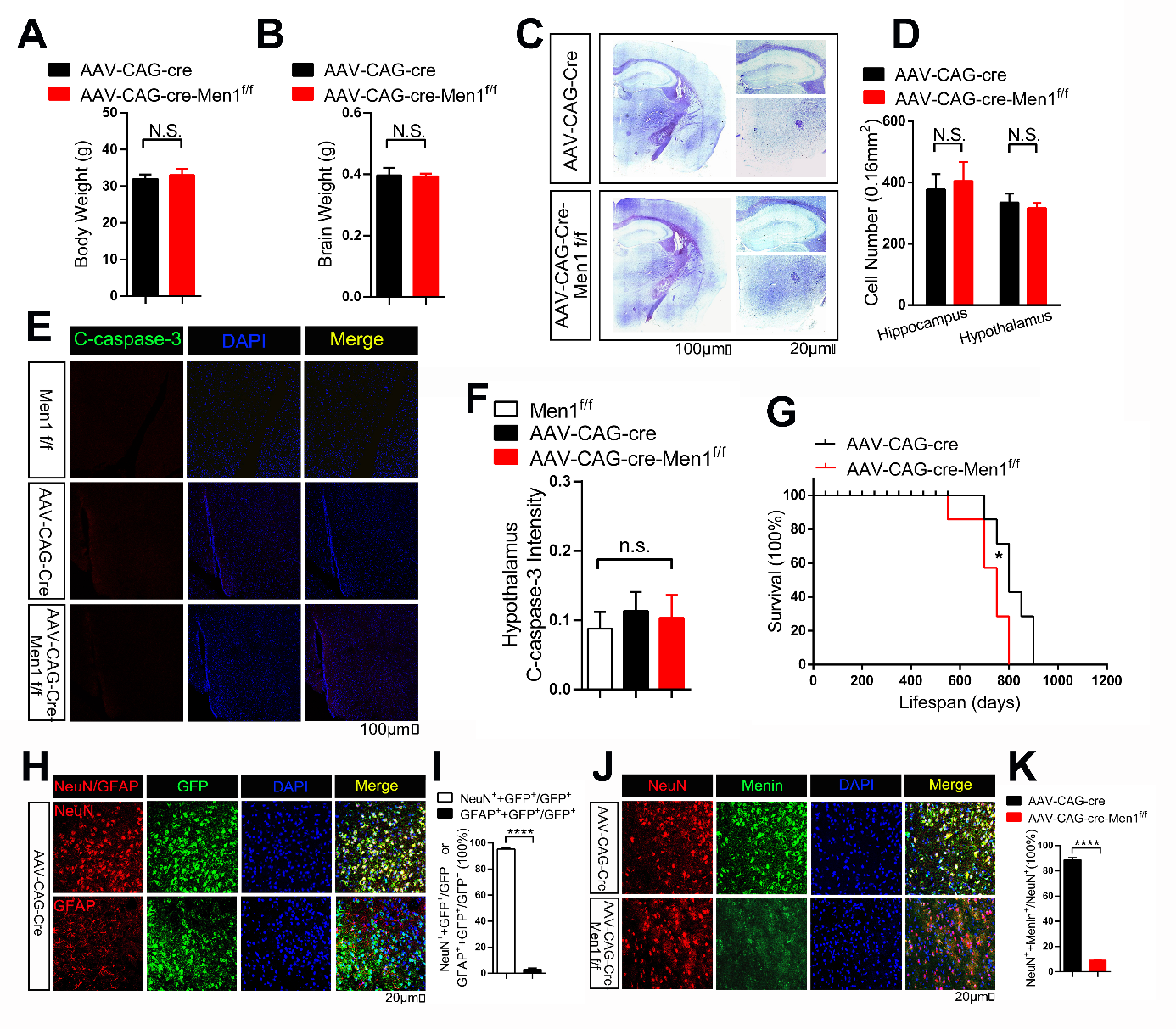

Supplement: S5 Fig — (TIF) [file pbio.3002033.s005.tif]

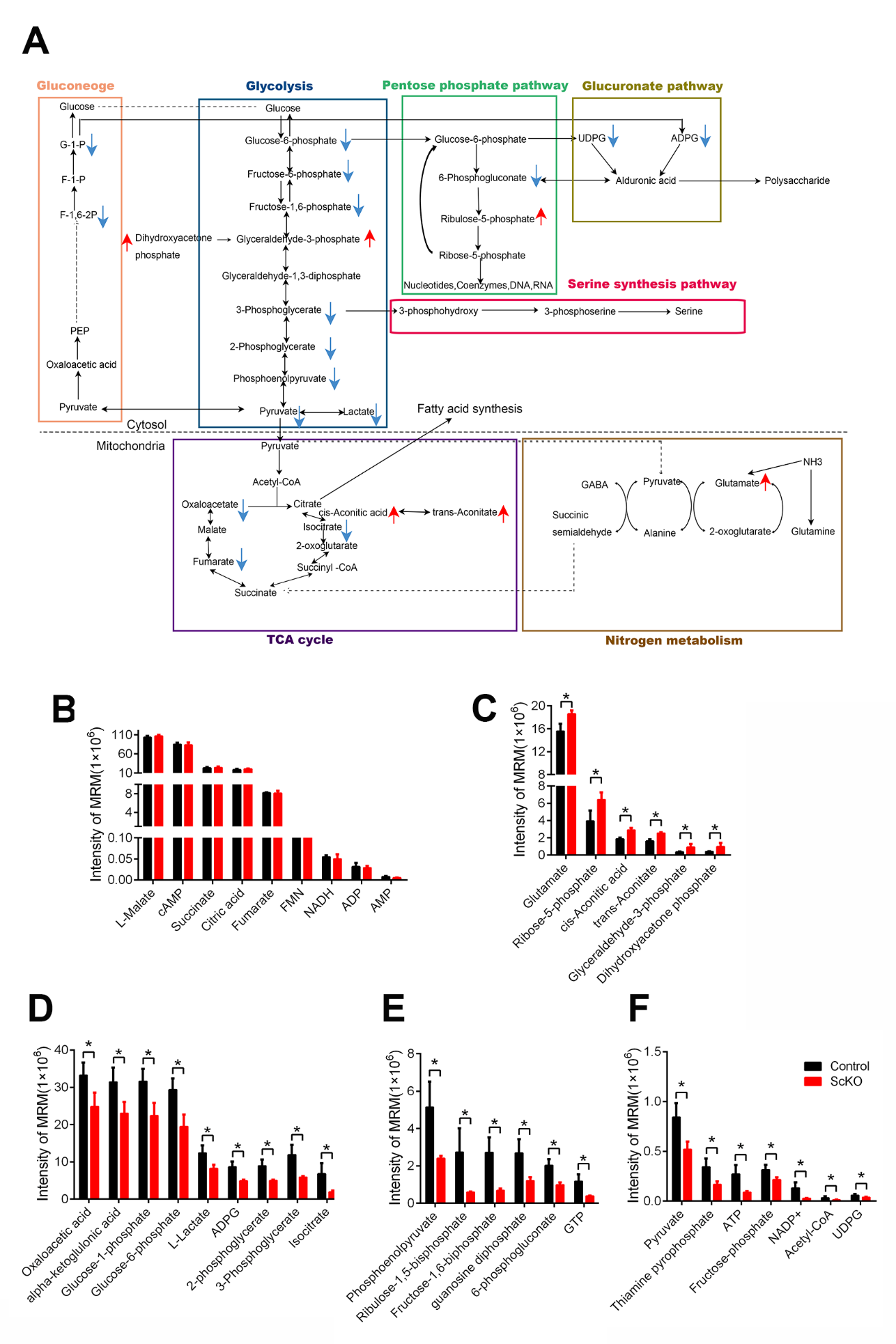

Supplement: S6 Fig — (TIF) [file pbio.3002033.s006.tif]

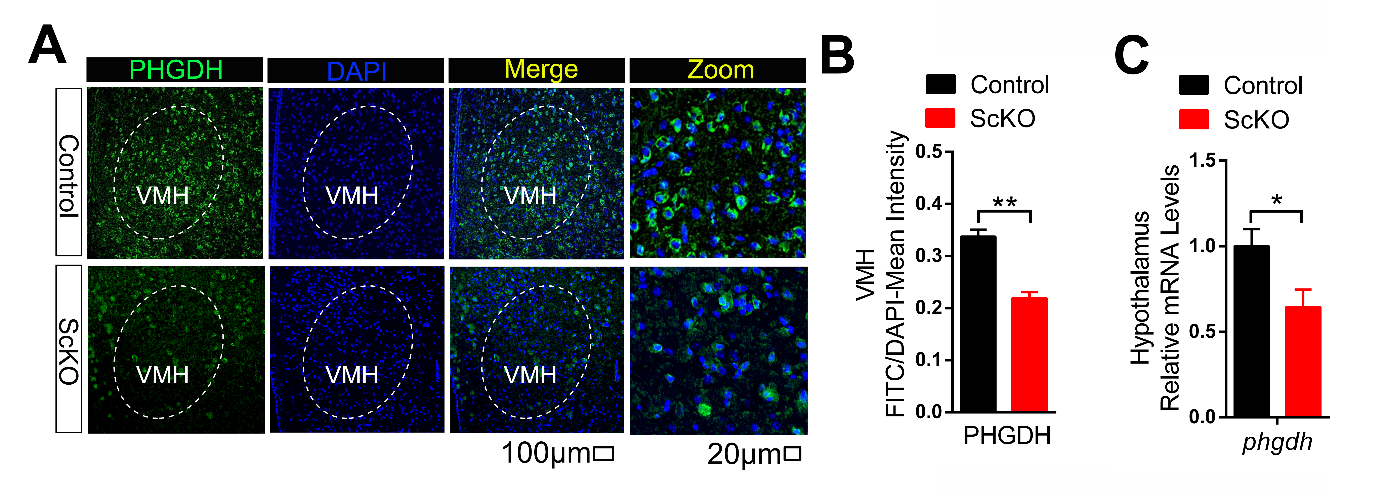

Supplement: S7 Fig — (TIF) [file pbio.3002033.s007.tif]

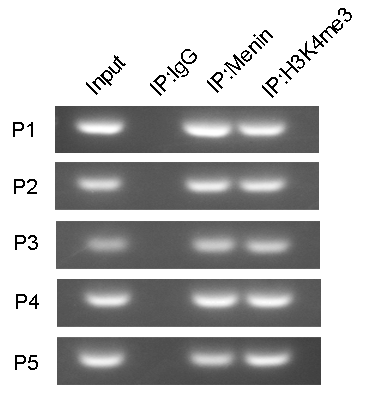

Supplement: S8 Fig — (TIF) [file pbio.3002033.s008.tif]

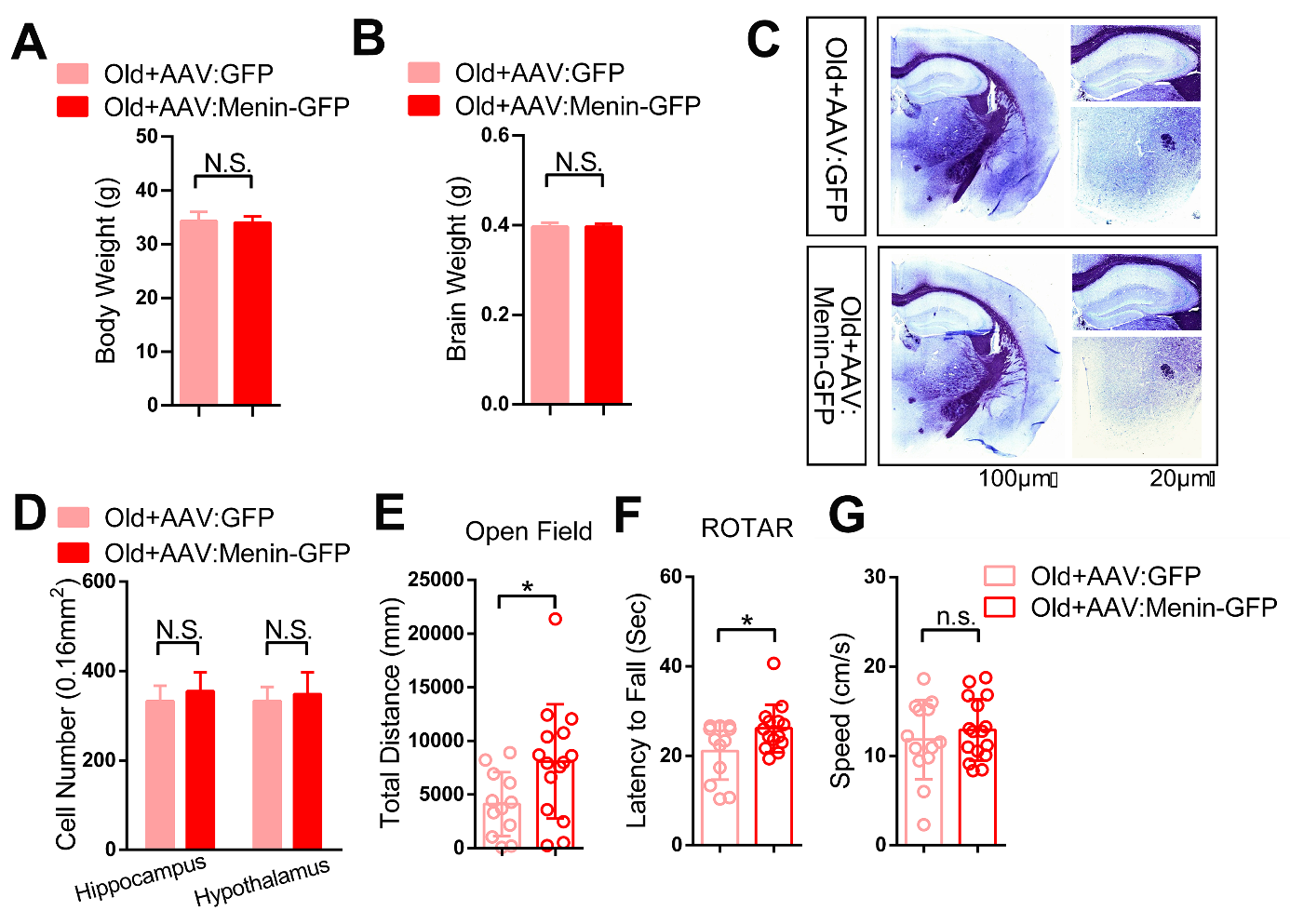

Supplement: S9 Fig — (TIF) [file pbio.3002033.s009.tif]

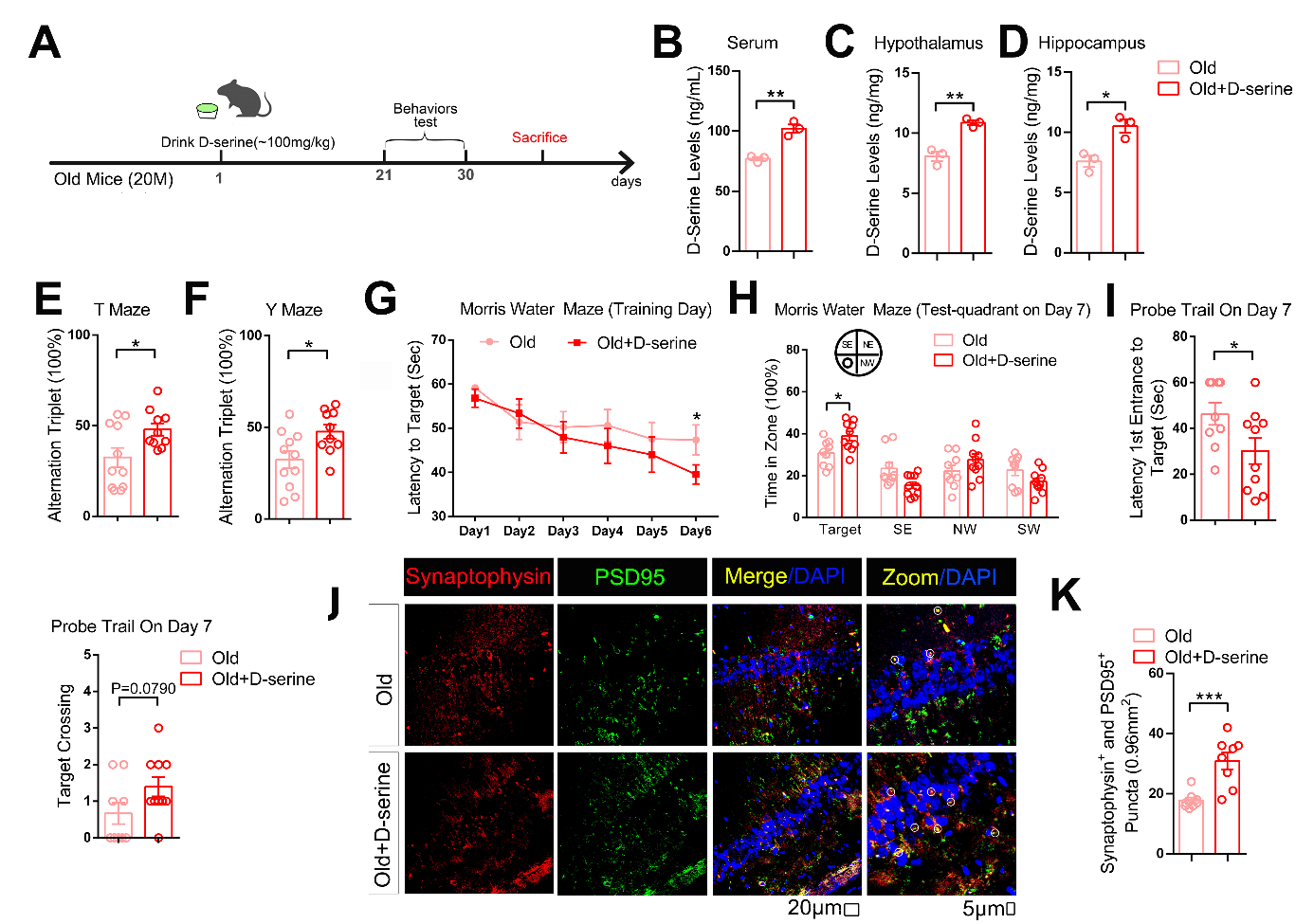

Supplement: S10 Fig — (TIF) [file pbio.3002033.s010.tif]
