# Supplementary material for: Hypothalamic Menin regulates systemic aging and cognitive decline
Source: PLoS Biol. 2023 Mar 16;21(3):e3002033. doi: 10.1371/journal.pbio.3002033 (PMC10019680; doi:10.1371/journal.pbio.3002033)
Supplement: S1 Raw images — (PDF) [file pbio.3002033.s012.pdf]

SourceData_Gel_Fig 1

Fig 1A

Menin

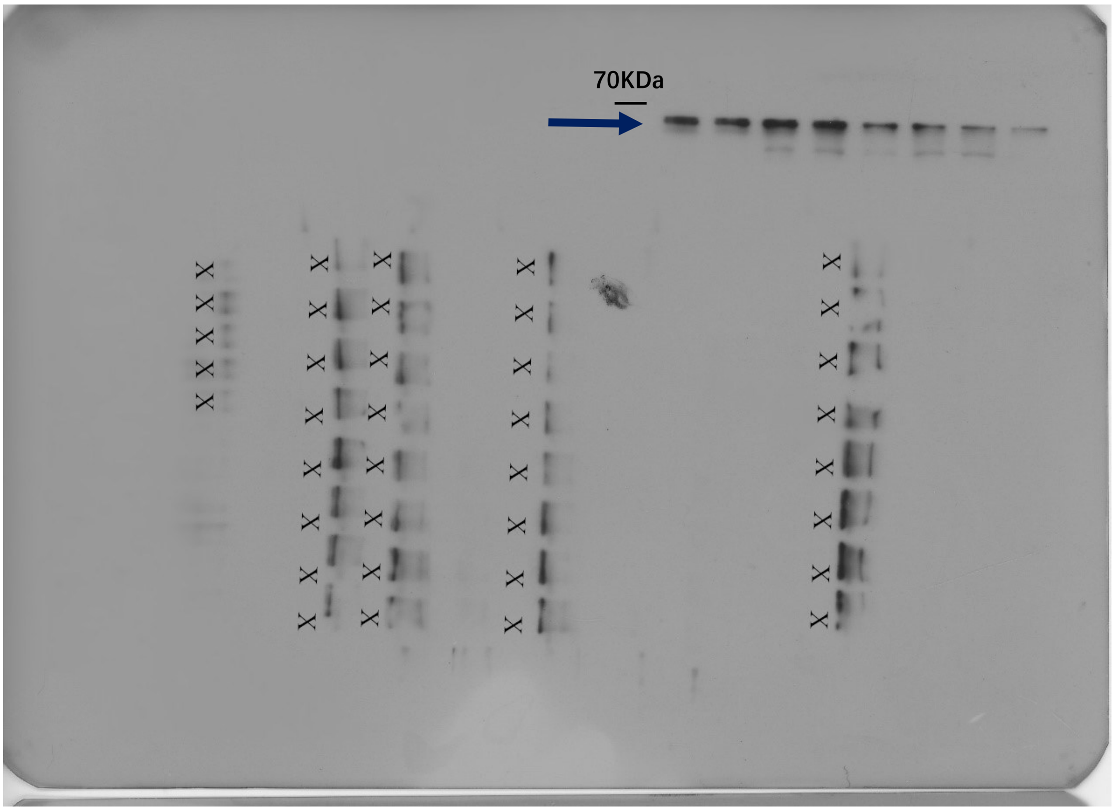

Loading order ⟶

Identity of experimental samples:

lane 1-4：Protein samples from the hypothalamus of young (3M) mice 1-4; lane 5-8：Protein samples from the hypothalamus of old (20M) mice 1-4.

Method used to capture the image:

Chemiluminescence. The bands were captured by developer and fixing solution on medical blue sensitive x-ray film (Baiyun, China), then the bands on the blots were scanned and analyzed with ImageJ (National Institutes of Health, Bethesda, MD, USA).

Actin

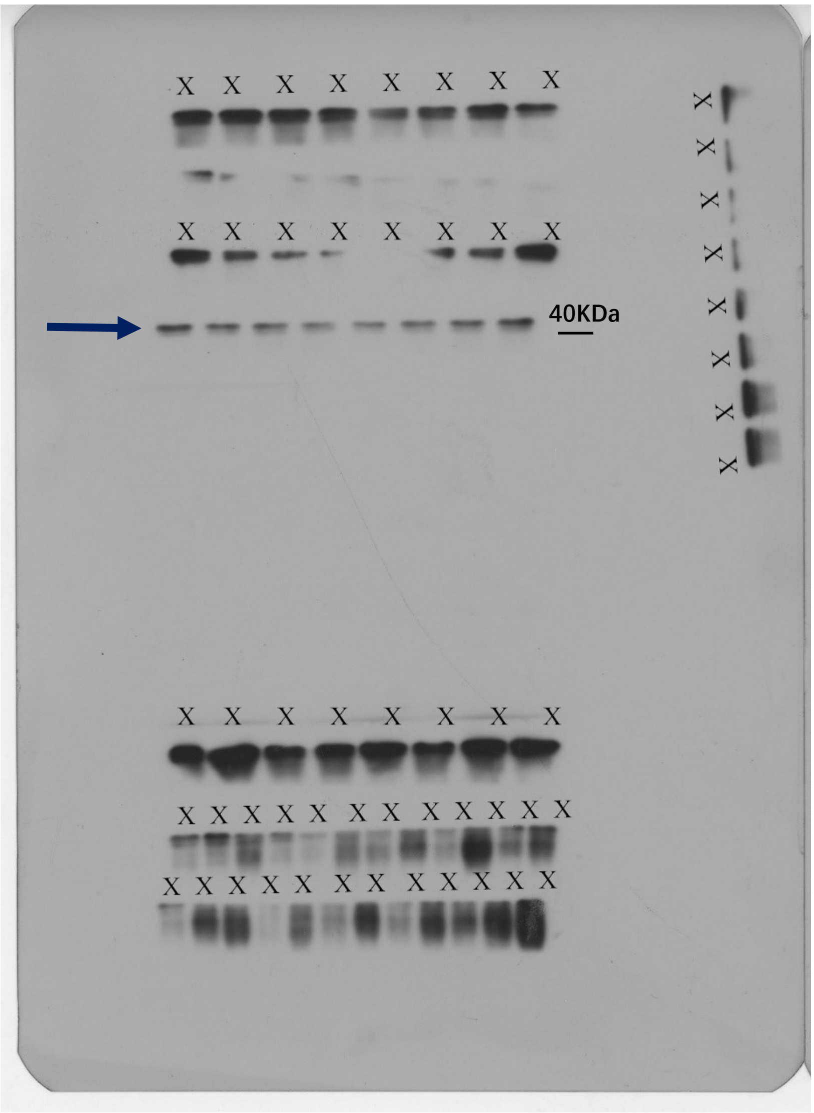

Loading order ——————————→

Identity of experimental samples:

lane 1-4: Protein samples from the hypothalamus of young (3M) mice 1-4; lane 5-8: Protein samples from the hypothalamus of old (20M) mice 1-4.

Method used to capture the image:

Chemiluminescence. The bands were captured by developer and fixing solution on medical blue sensitive x-ray film (Baiyun, China), then the bands on the blots were scanned and analyzed with ImageJ (National Institutes of Health, Bethesda, MD, USA).

Fig 1E
Menin

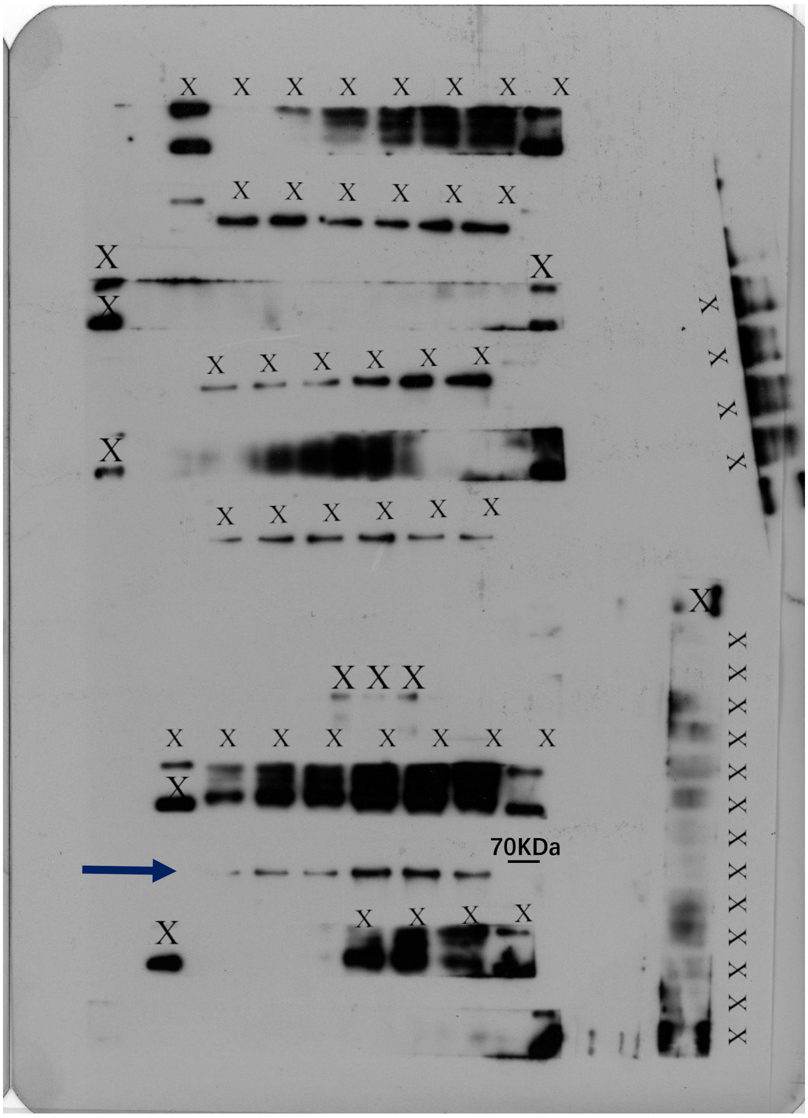

Loading order ——————————→

Identity of experimental samples:

lane 1-3：Protein samples from the hypothalamus of young (3M) mice 1-3; lane 4-6：Protein samples from the hypothalamus of old (20M) mice 1-3.

Method used to capture the image:

Chemiluminescence. The bands were captured by developer and fixing solution on medical blue sensitive x-ray film (Baiyun, China), then the bands on the blots were scanned and analyzed with ImageJ (National Institutes of Health, Bethesda, MD, USA).

Actin

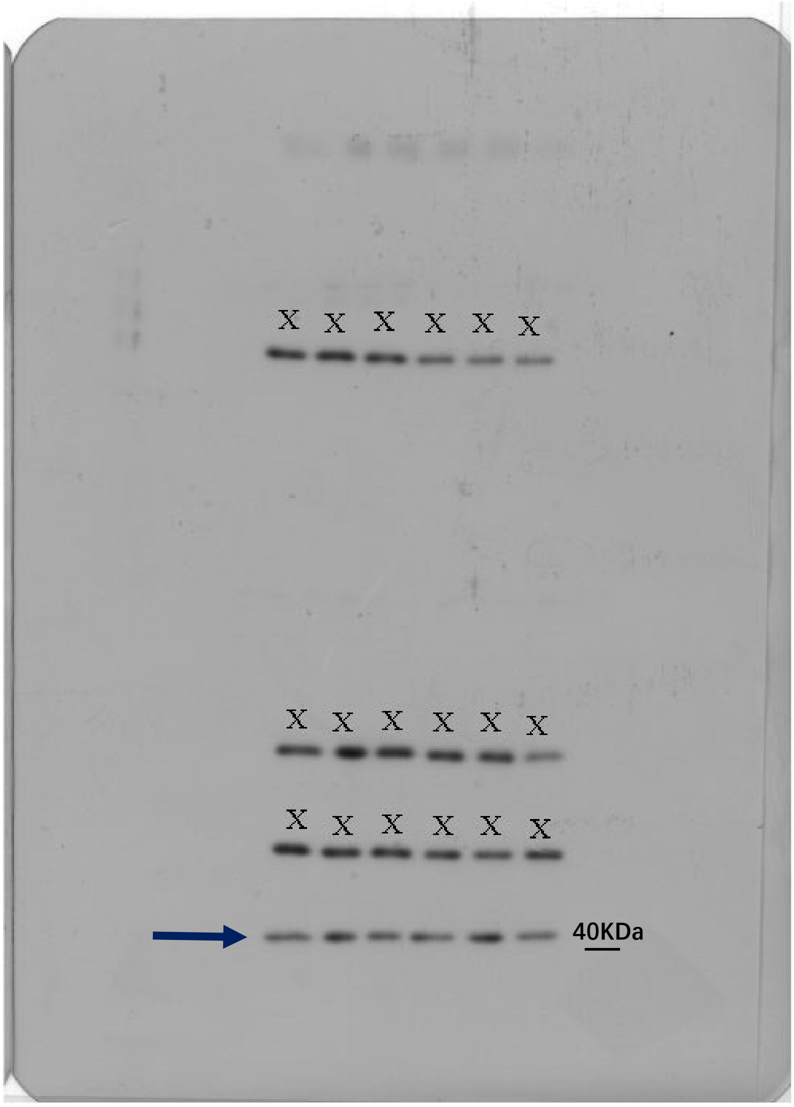

Loading order ⟶

Identity of experimental samples:

lane 1-3：Protein samples from the hypothalamus of young (3M) mice 1-3; lane 4-6：Protein samples from the hypothalamus of old (20M) mice 1-3.

Method used to capture the image:

Chemiluminescence. The bands were captured by developer and fixing solution on medical blue sensitive x-ray film (Baiyun, China), then the bands on the blots were scanned and analyzed with ImageJ (National Institutes of Health, Bethesda, MD, USA).

Fig 1I
Menin

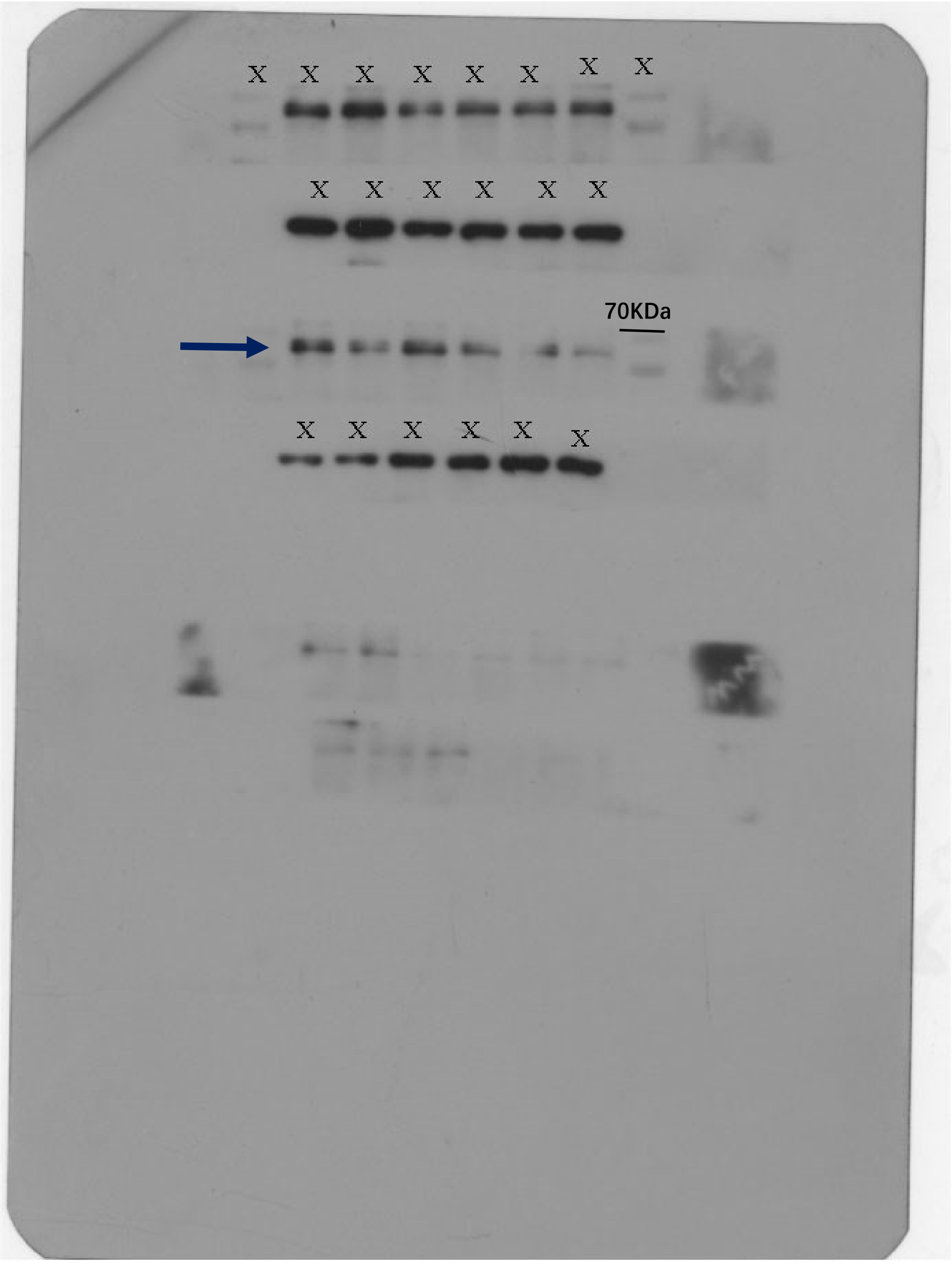

Loading order ⟶

Identity of experimental samples:

lane 1-3: Protein samples from the hypothalamus of 6M *Men1*$^{f/f}$ mice 1-3; lane 4-6: Protein samples from the hypothalamus of 6M ScKO mice 1-3.

Method used to capture the image:

Chemiluminescence. The bands were captured by developer and fixing solution on medical blue sensitive x-ray film (Baiyun, China), then the bands on the blots were scanned and analyzed with ImageJ (National Institutes of Health, Bethesda, MD, USA).

Actin

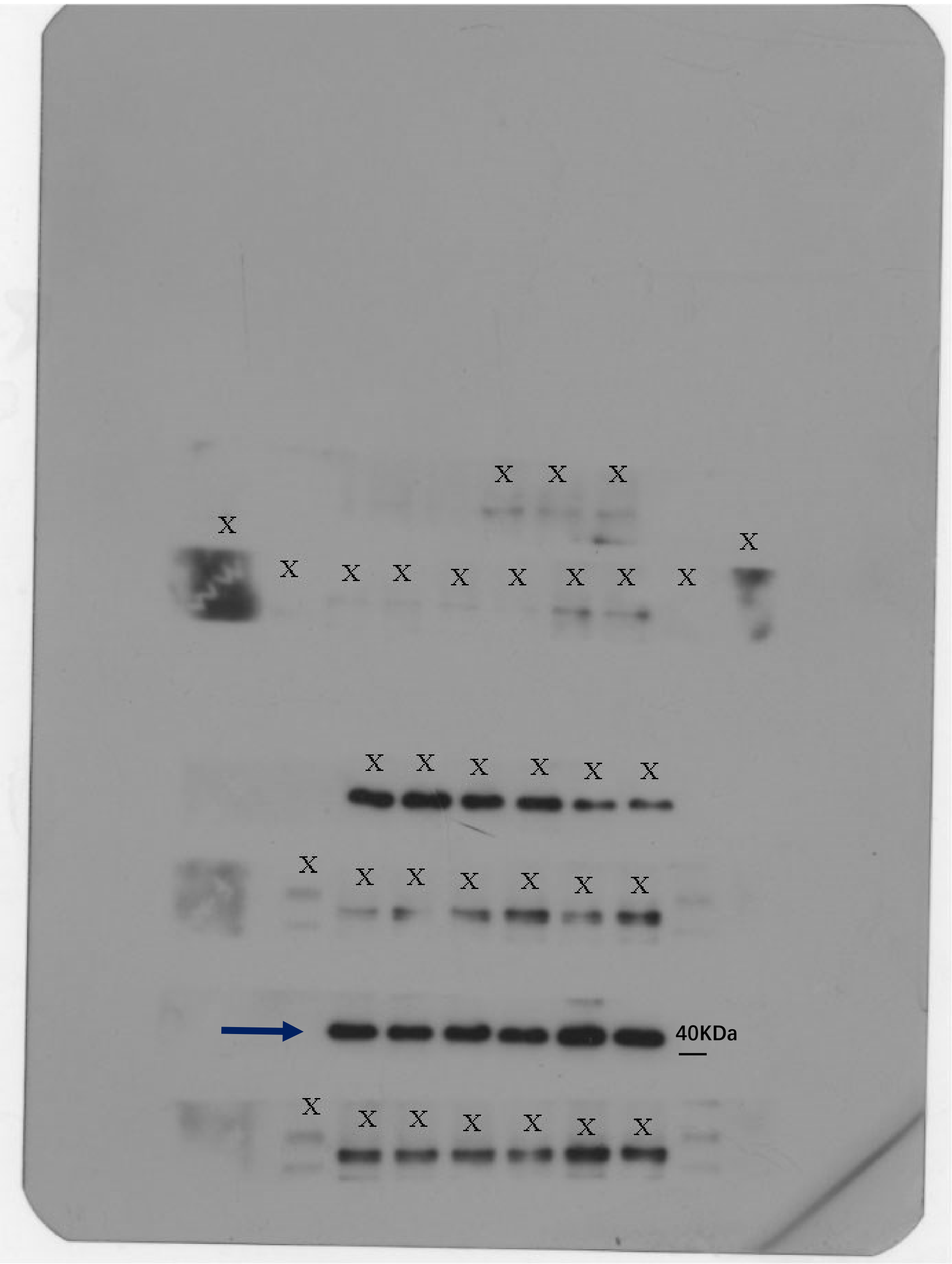

loading order ⟶

Identity of experimental samples:

lane 1-3: Protein samples from the hypothalamus of 6M *Men1*$^{f/f}$ mice 1-3; lane 4-6: Protein samples from the hypothalamus of 6M ScKO mice 1-3.

Method used to capture the image:

Chemiluminescence. The bands were captured by developer and fixing solution on medical blue sensitive x-ray film (Baiyun, China), then the bands on the blots were scanned and analyzed with ImageJ (National Institutes of Health, Bethesda, MD, USA).

Fig 1M
IL-1β

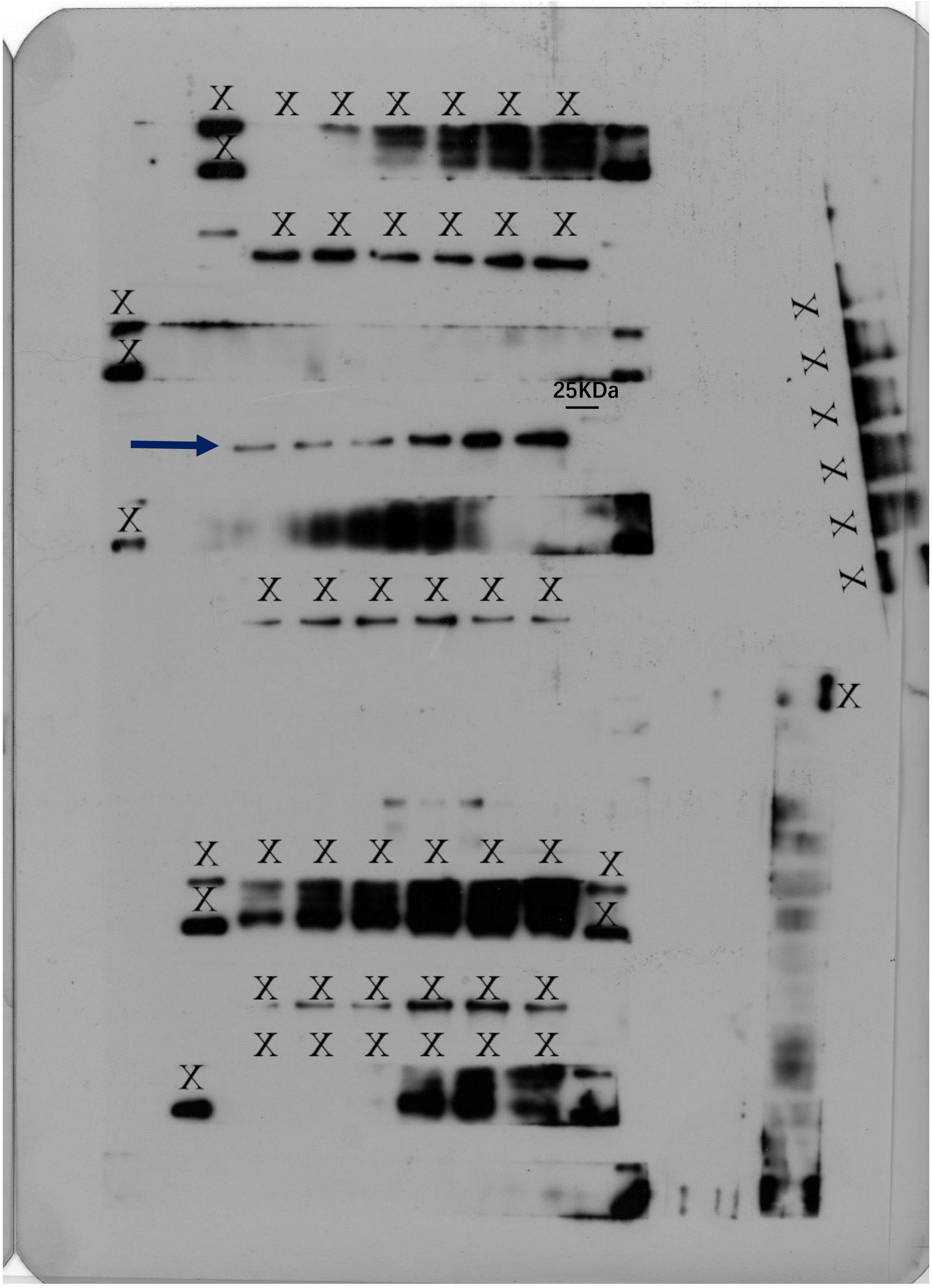

Loading order ⟶

Identity of experimental samples:

lane 1-3: Protein samples from the hypothalamus of 6M *Men1*$^{f/f}$ mice 1-3; lane 4-6: Protein samples from the hypothalamus of 6M ScKO mice 1-3.

Method used to capture the image:

Chemiluminescence. The bands were captured by developer and fixing solution on medical blue sensitive x-ray film (Baiyun, China), then the bands on the blots were scanned and analyzed with ImageJ (National Institutes of Health, Bethesda, MD, USA).

Actin

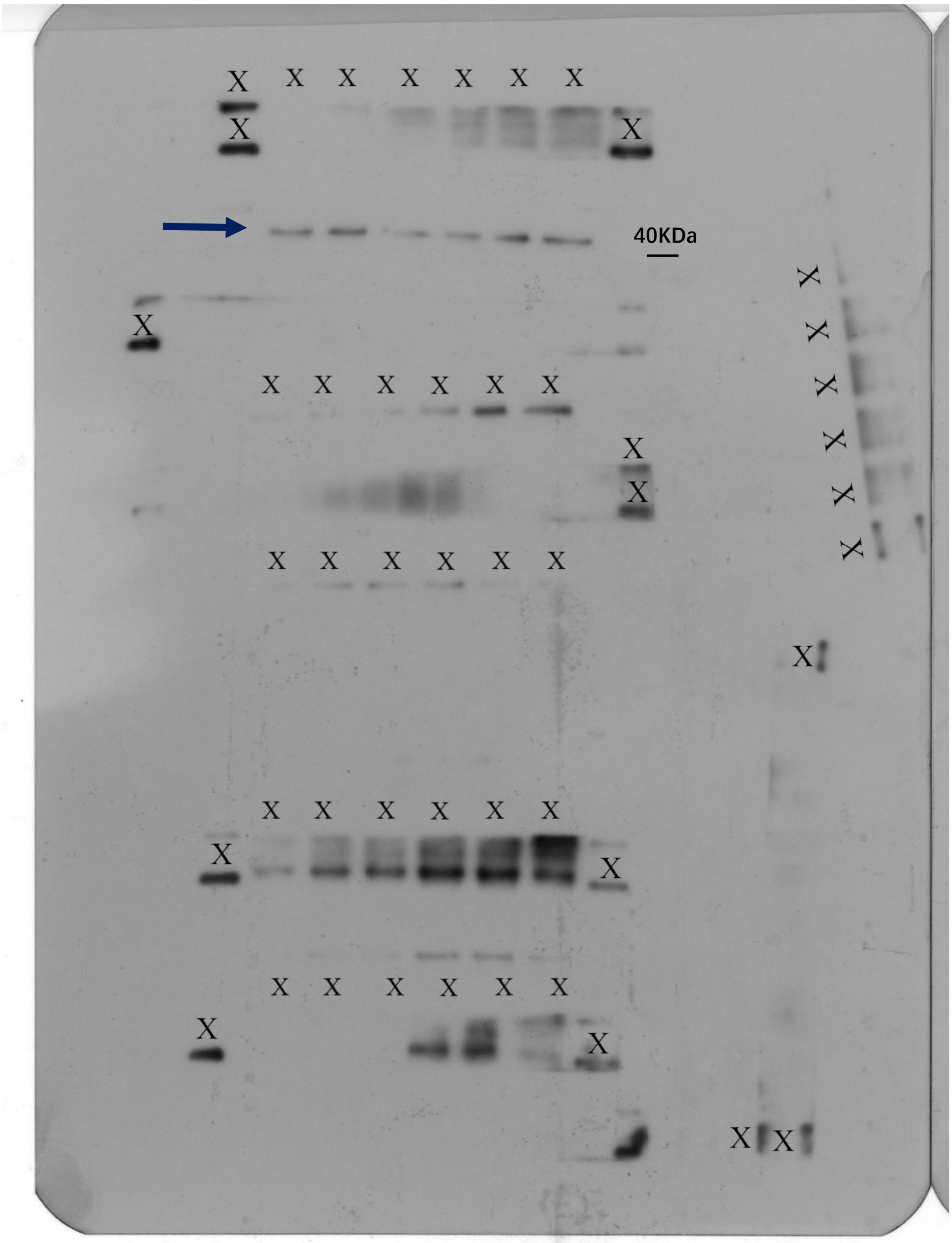

Loading order ⟶

Identity of experimental samples:

lane 1-3：Protein samples from the hypothalamus of 6M *Men1*$^{f/f}$ mice 1-3; lane 4-6：Protein samples from the hypothalamus of 6M ScKO mice 1-3.

Method used to capture the image:

Chemiluminescence. The bands were captured by developer and fixing solution on medical blue sensitive x-ray film (Baiyun, China), then the bands on the blots were scanned and analyzed with ImageJ (National Institutes of Health, Bethesda, MD, USA).

SourceData_Gel_Fig 2

Fig 2D

Menin

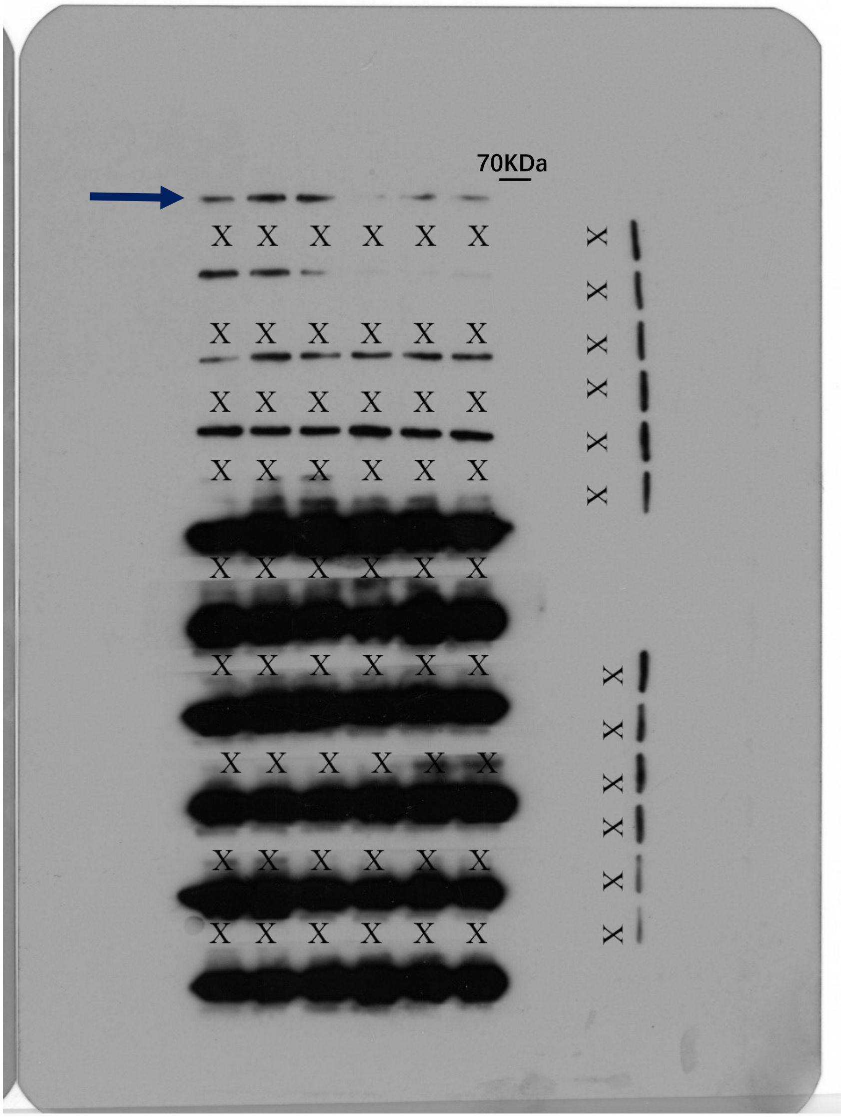

Loading order ⟶

Identity of experimental samples:

lane 1-3: Protein samples from the hypothalamus of 10M AAV-CAG-Cre mice 1-3; lane 4-6: Protein samples from the hypothalamus of 10M AAV-CAG-Cre-*Men1*$^{f/f}$ mice 1-3.

Method used to capture the image:

Chemiluminescence. The bands were captured by developer and fixing solution on medical blue sensitive x-ray film (Baiyun, China), then the bands on the blots were scanned and analyzed with ImageJ (National Institutes of Health, Bethesda, MD, USA).

Actin

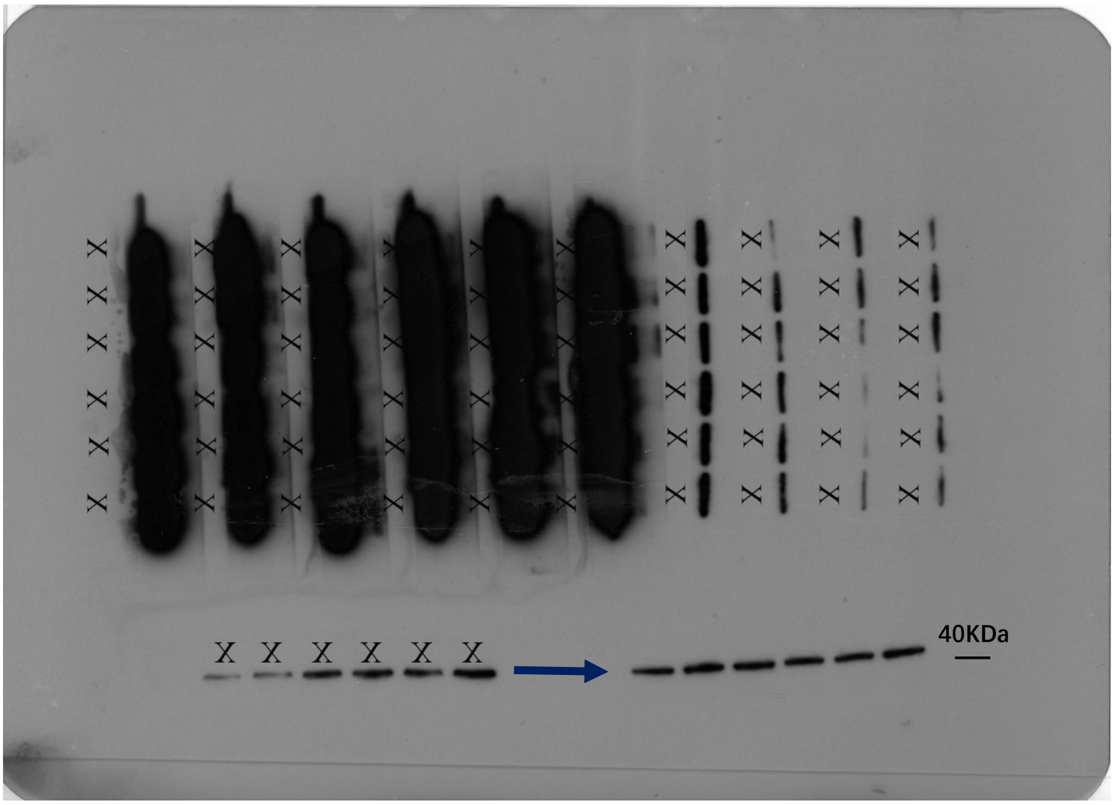

Loading order⟶

Identity of experimental samples:

lane 1-3: Protein samples from the hypothalamus of 10M AAV-CAG-Cre mice 1-3; lane 4-6: Protein samples from the hypothalamus of 10M AAV-CAG-Cre-*Men1*$^{f/f}$ mice 1-3.

Method used to capture the image:

Chemiluminescence. The bands were captured by developer and fixing solution on medical blue sensitive x-ray film (Baiyun, China), then the bands on the blots were scanned and analyzed with ImageJ (National Institutes of Health, Bethesda, MD, USA).

Fig 2P

IL-1β

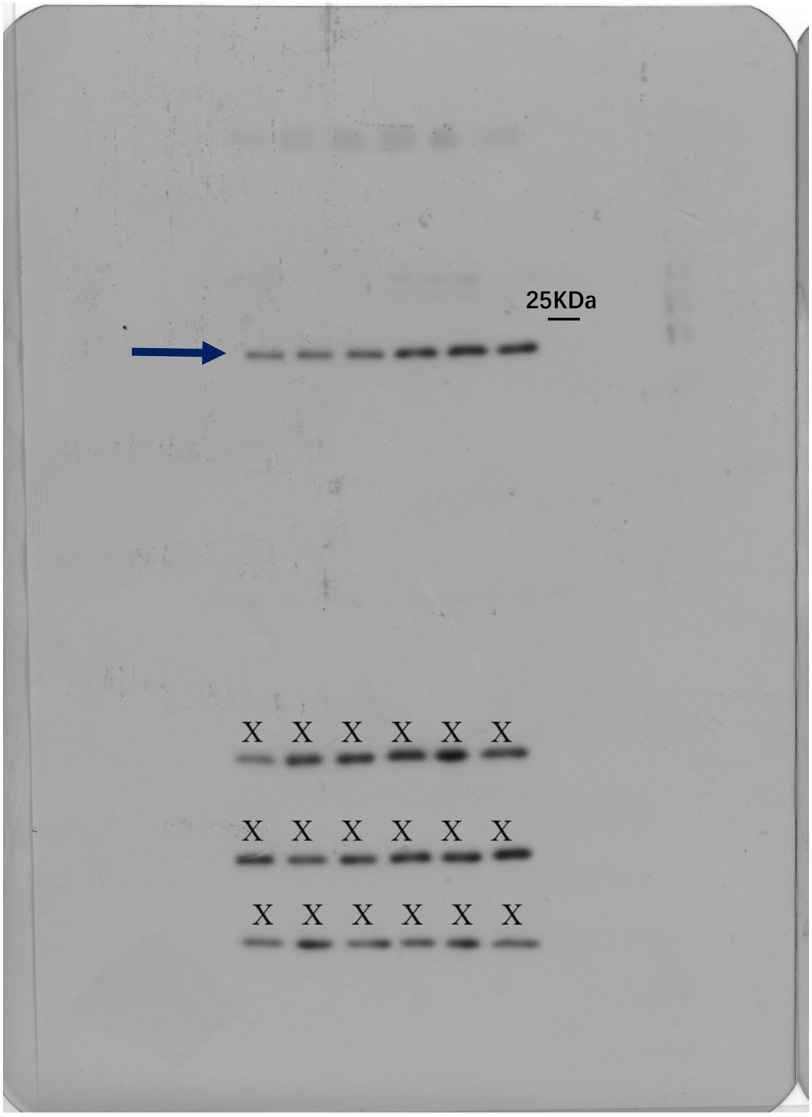

Loading order ⟶

Identity of experimental samples:

lane 1-3: Protein samples from the hypothalamus of 10M AAV-CAG-Cre mice 1-3; lane 4-6: Protein samples from the hypothalamus of 10M AAV-CAG-Cre-*Men1*$^{f/f}$ mice 1-3.

Method used to capture the image:

Chemiluminescence. The bands were captured by developer and fixing solution on medical blue sensitive x-ray film (Baiyun, China), then the bands on the blots were scanned and analyzed with ImageJ (National Institutes of Health, Bethesda, MD, USA).

Actin

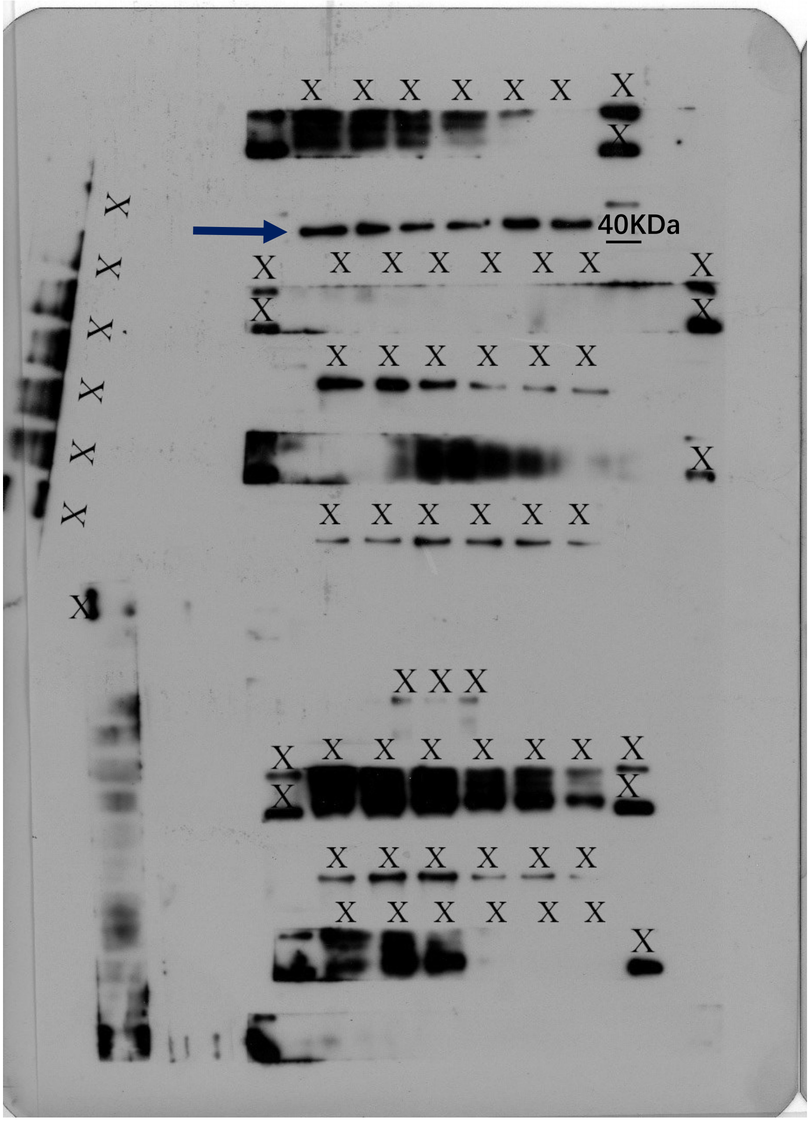

Loading order ⟶

Identity of experimental samples:

lane 1-3: Protein samples from the hypothalamus of 10M AAV-CAG-Cre mice 1-3; lane 4-6: Protein samples from the hypothalamus of 10M AAV-CAG-Cre-*Men1*$^{f/f}$ mice 1-3.

Method used to capture the image:

Chemiluminescence. The bands were captured by developer and fixing solution on medical blue sensitive x-ray film (Baiyun, China), then the bands on the blots were scanned and analyzed with ImageJ (National Institutes of Health, Bethesda, MD, USA).

SourceData_Gel_Fig 4

Fig 4D

PHGDH

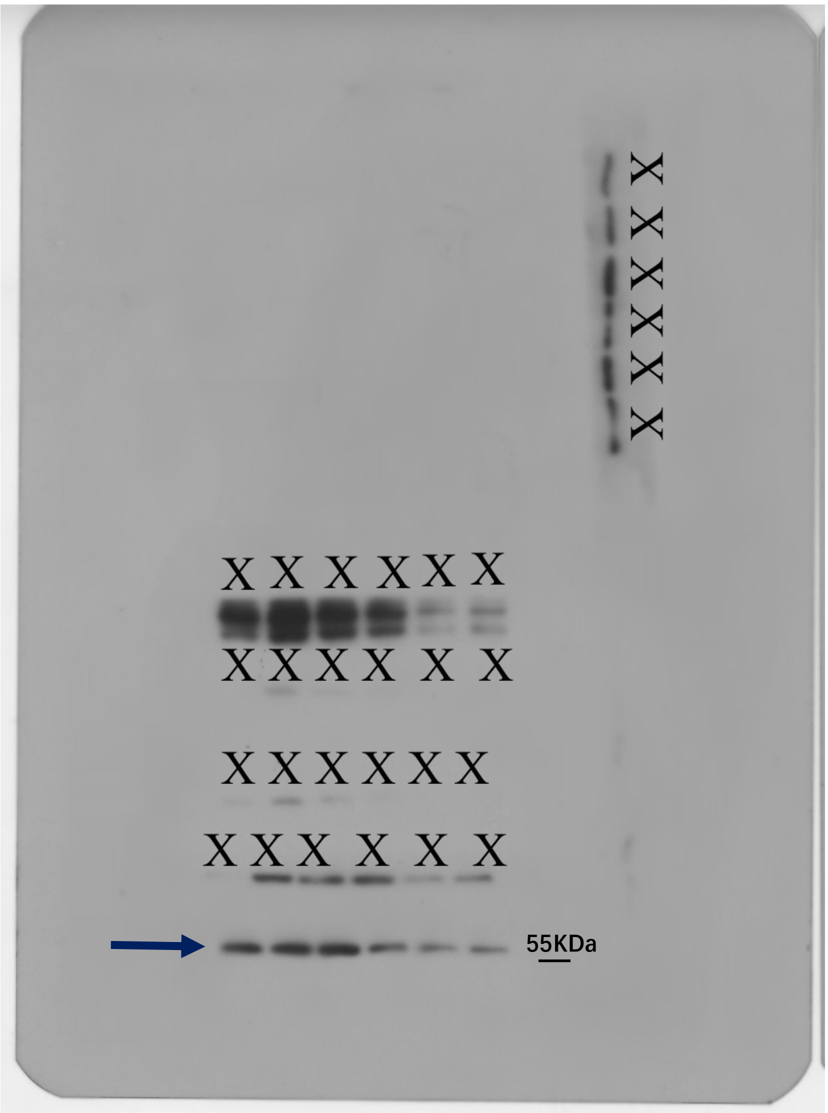

Loading order ————————————→

Identity of experimental samples:

lane 1-3: Protein samples from the hypothalamus of 10M AAV-CAG-Cre mice 1-3; lane 4-6: Protein samples from the hypothalamus of 10M AAV-CAG-Cre-*Men1*$^{f/f}$ mice 1-3.

Method used to capture the image:

Chemiluminescence. The bands were captured by developer and fixing solution on medical blue sensitive x-ray film (Baiyun, China), then the bands on the blots were scanned and analyzed with ImageJ (National Institutes of Health, Bethesda, MD, USA).

Actin

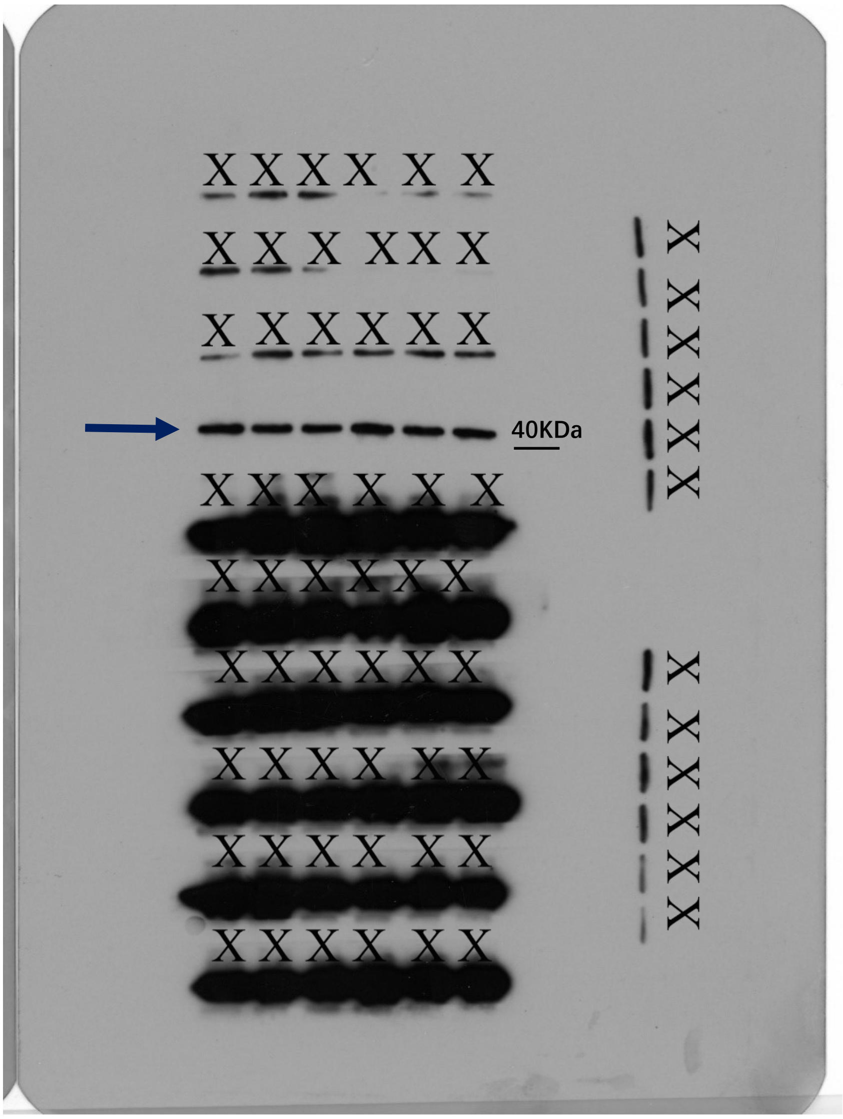

Loading order ⟶

Identity of experimental samples:

lane 1-3: Protein samples from the hypothalamus of 10M AAV-CAG-Cre mice 1-3; lane 4-6: Protein samples from the hypothalamus of 10M AAV-CAG-Cre-*Men1*$^{f/f}$ mice 1-3.

Method used to capture the image:

Chemiluminescence. The bands were captured by developer and fixing solution on medical blue sensitive x-ray film (Baiyun, China), then the bands on the blots were scanned and analyzed with ImageJ (National Institutes of Health, Bethesda, MD, USA).

Fig 4L
Menin

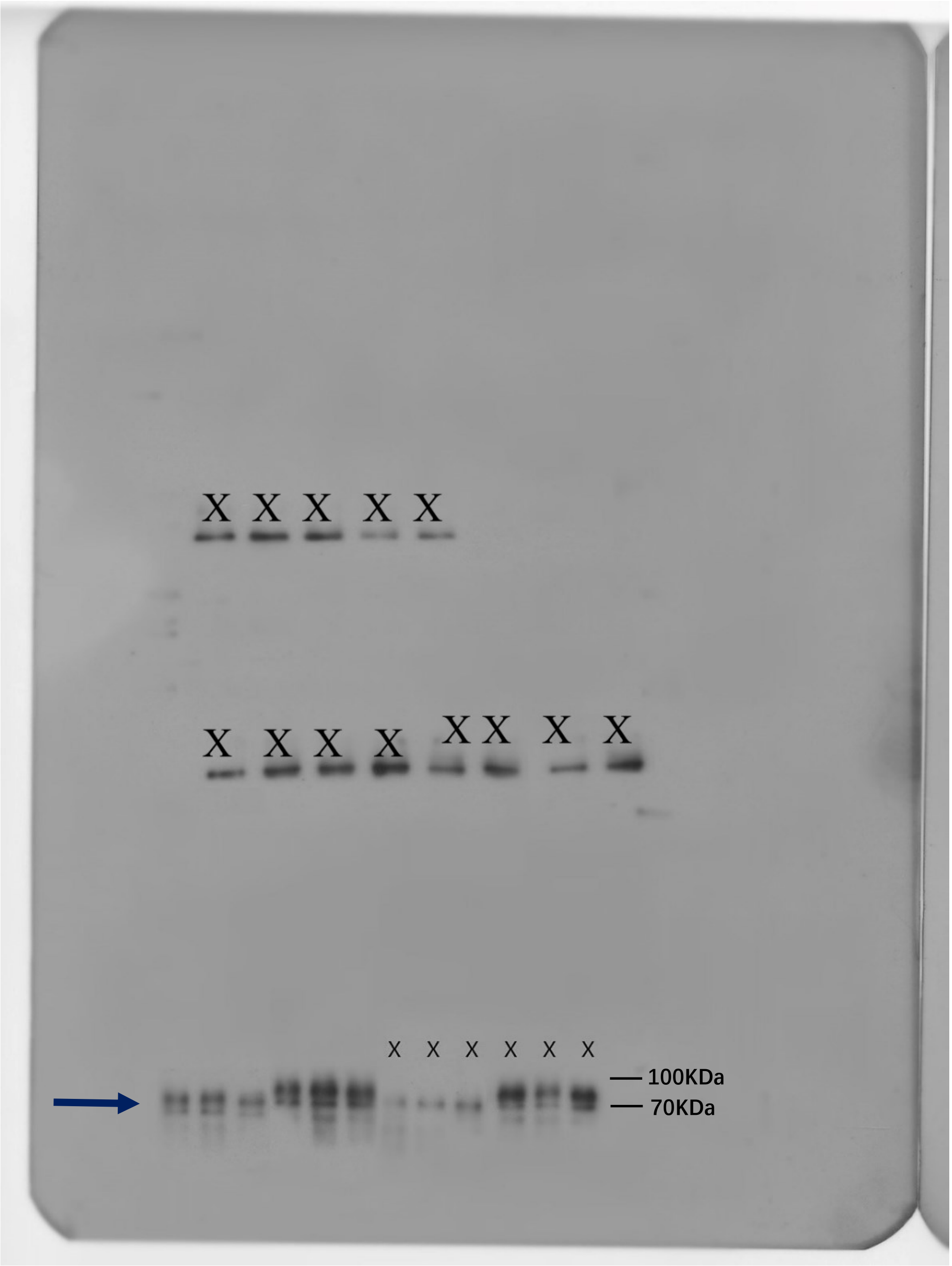

Loading order ⟶

Identity of experimental samples:

lane 1-3: Protein samples from the hypothalamus of 20M AAV-GFP mice 1-3; lane 4-6: Protein samples from the hypothalamus of 20M AAV-Menin-GFP mice 1-3.

Method used to capture the image:

Chemiluminescence. The bands were captured by developer and fixing solution on medical blue sensitive x-ray film (Baiyun, China), then the bands on the blots were scanned and analyzed with ImageJ (National Institutes of Health, Bethesda, MD, USA).

PHGDH

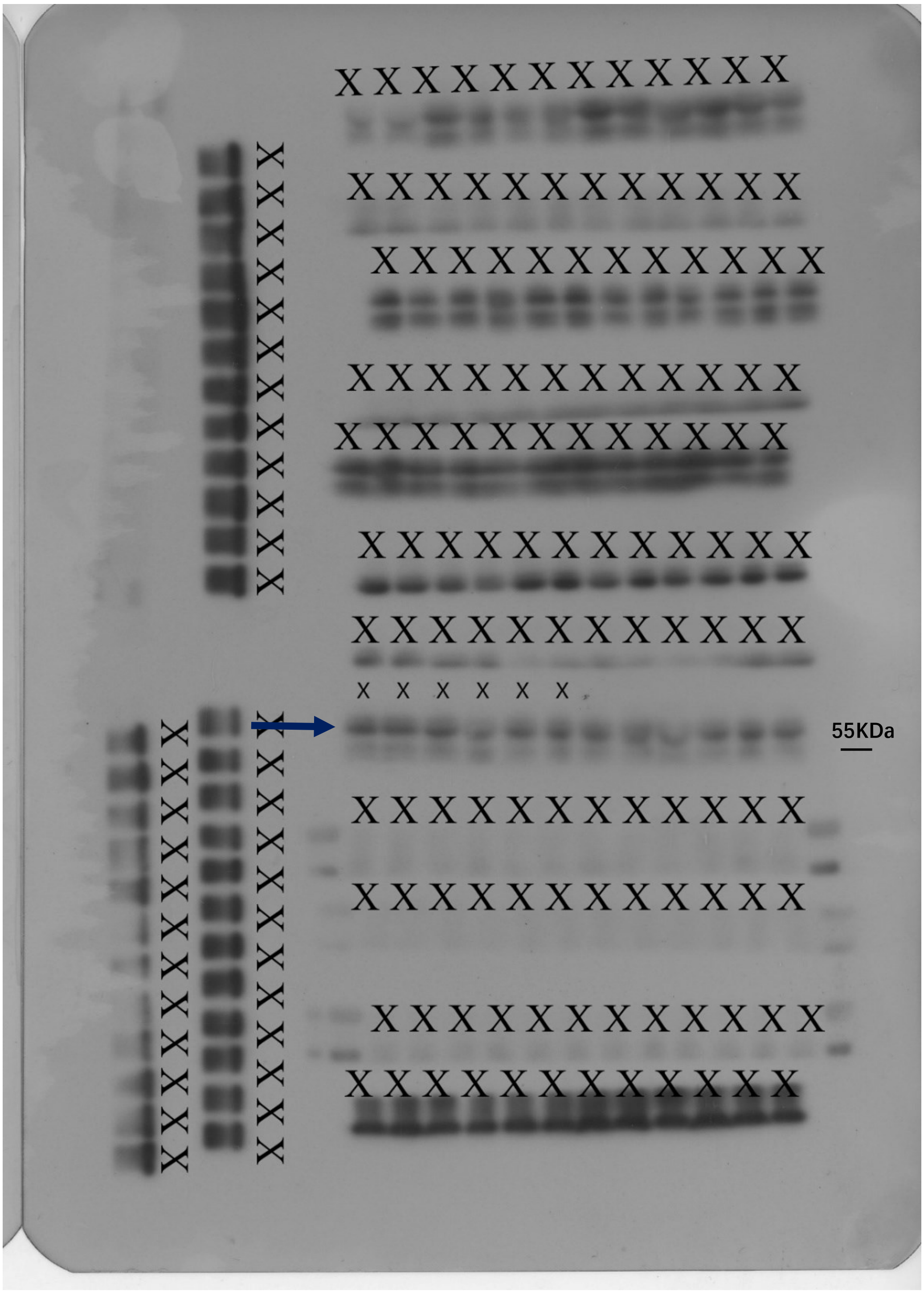

Loading order ⟶

Identity of experimental samples:

lane 7-9: Protein samples from the hypothalamus of 20M AAV-GFP mice 1-3; lane 10-12: Protein samples from the hypothalamus of 20M AAV-Menin-GFP mice 1-3.

Method used to capture the image:

Chemiluminescence. The bands were captured by developer and fixing solution on medical blue sensitive x-ray film (Baiyun, China), then the bands on the blots were scanned and analyzed with ImageJ (National Institutes of Health, Bethesda, MD, USA).

Actin

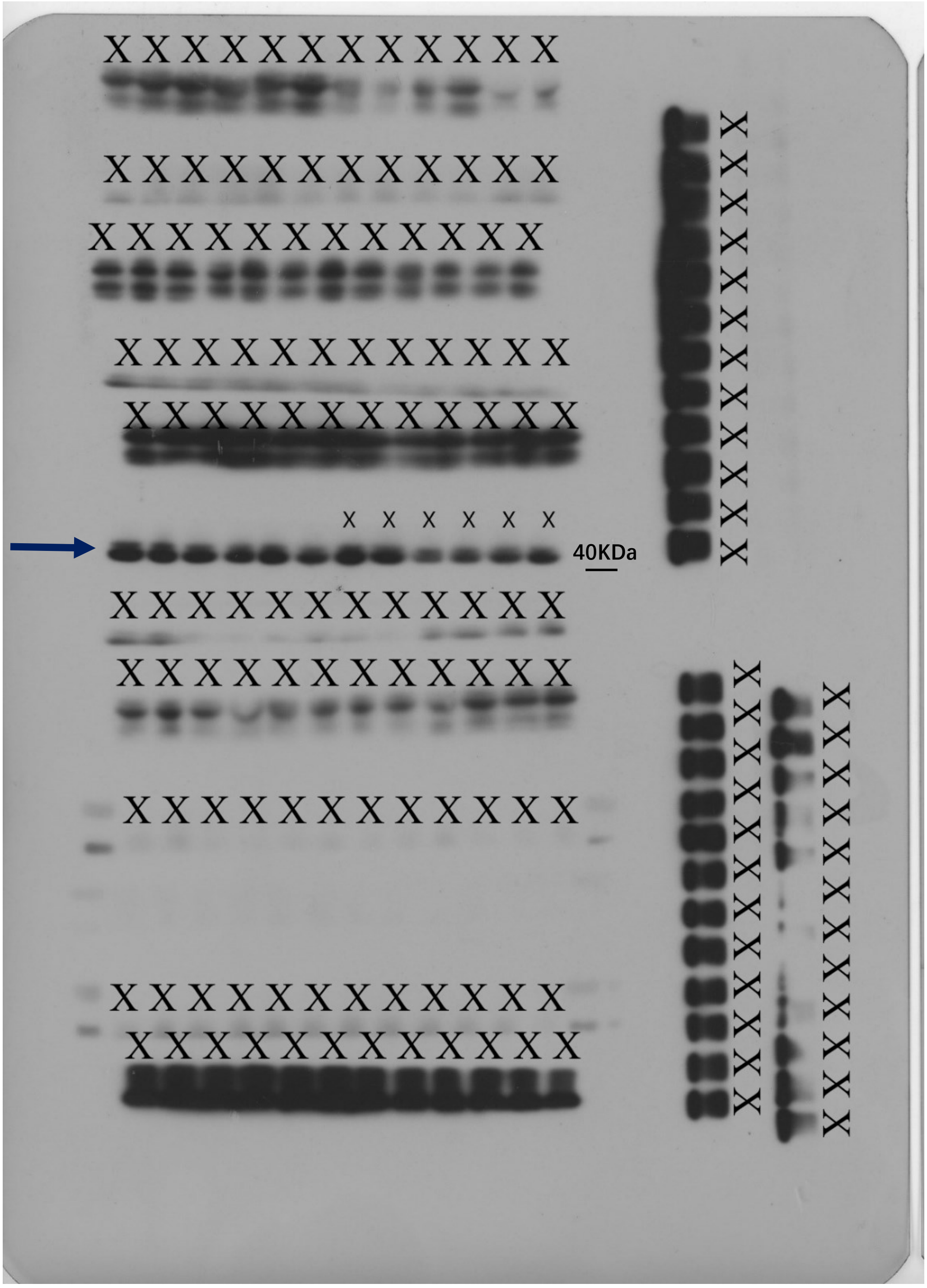

Loading order →

Identity of experimental samples:

lane 1-3: Protein samples from the hypothalamus of 20M AAV-GFP mice 1-3; lane 4-6: Protein samples from the hypothalamus of 20M AAV-Menin-GFP mice 1-3.

Method used to capture the image:

Chemiluminescence. The bands were captured by developer and fixing solution on medical blue sensitive x-ray film (Baiyun, China), then the bands on the blots were scanned and analyzed with ImageJ (National Institutes of Health, Bethesda, MD, USA).

SourceData_Gel_Fig 5

Fig 5J
IL-1β

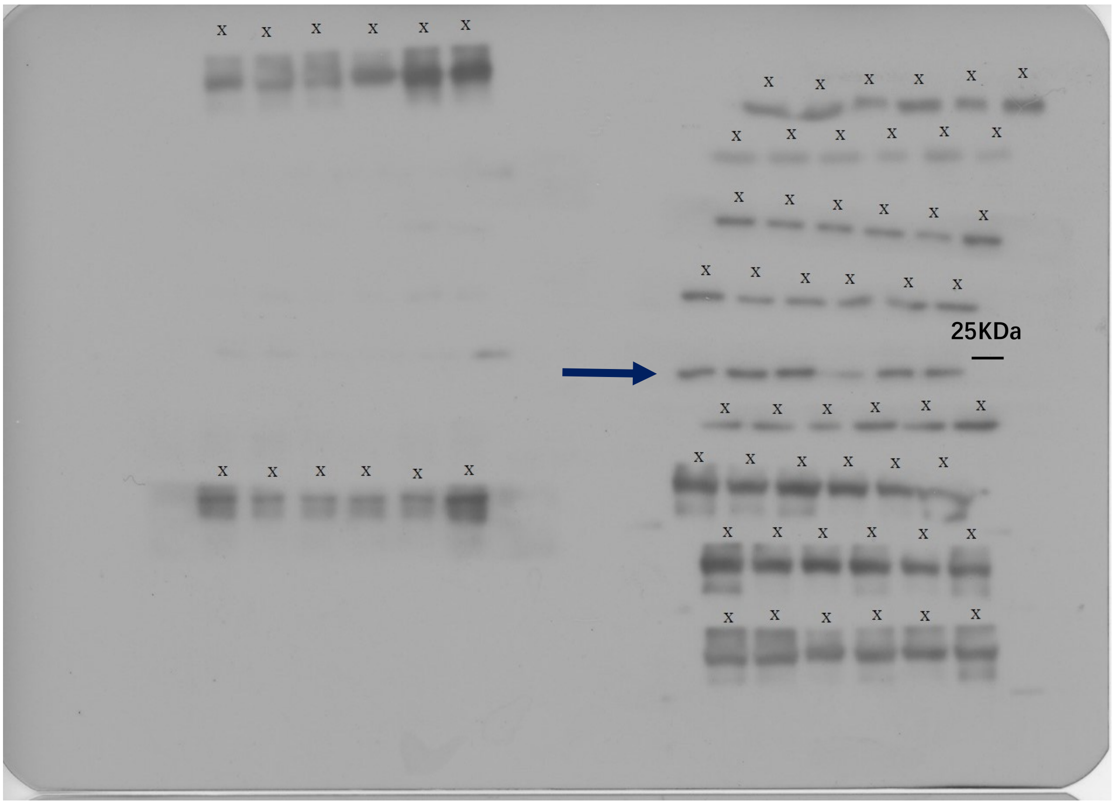

Loading order ⟶

Identity of experimental samples:

lane 1-3: Protein samples from the hypothalamus of 20M AAV-GFP mice 1-3; lane 4-6: Protein samples from the hypothalamus of 20M AAV-Menin-GFP mice 1-3.

Method used to capture the image:

Chemiluminescence. The bands were captured by developer and fixing solution on medical blue sensitive x-ray film (Baiyun, China), then the bands on the blots were scanned and analyzed with ImageJ (National Institutes of Health, Bethesda, MD, USA).

Actin

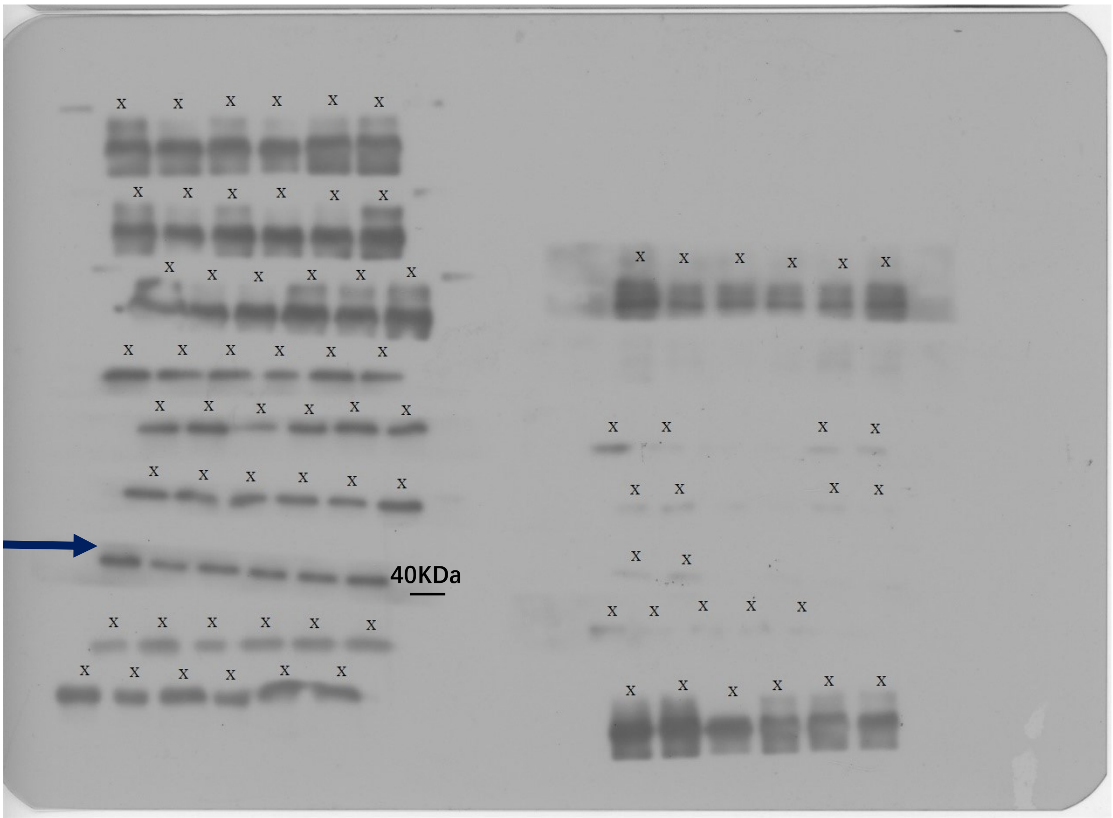

Loading order ⟶

Identity of experimental samples:

lane 1-3: Protein samples from the hypothalamus of 20M AAV-GFP mice 1-3; lane 4-6: Protein samples from the hypothalamus of 20M AAV-Menin-GFP mice 1-3.

Method used to capture the image:

Chemiluminescence. The bands were captured by developer and fixing solution on medical blue sensitive x-ray film (Baiyun, China), then the bands on the blots were scanned and analyzed with ImageJ (National Institutes of Health, Bethesda, MD, USA).

SourceData_Gel_S1_Fig

S1A _Fig

Menin

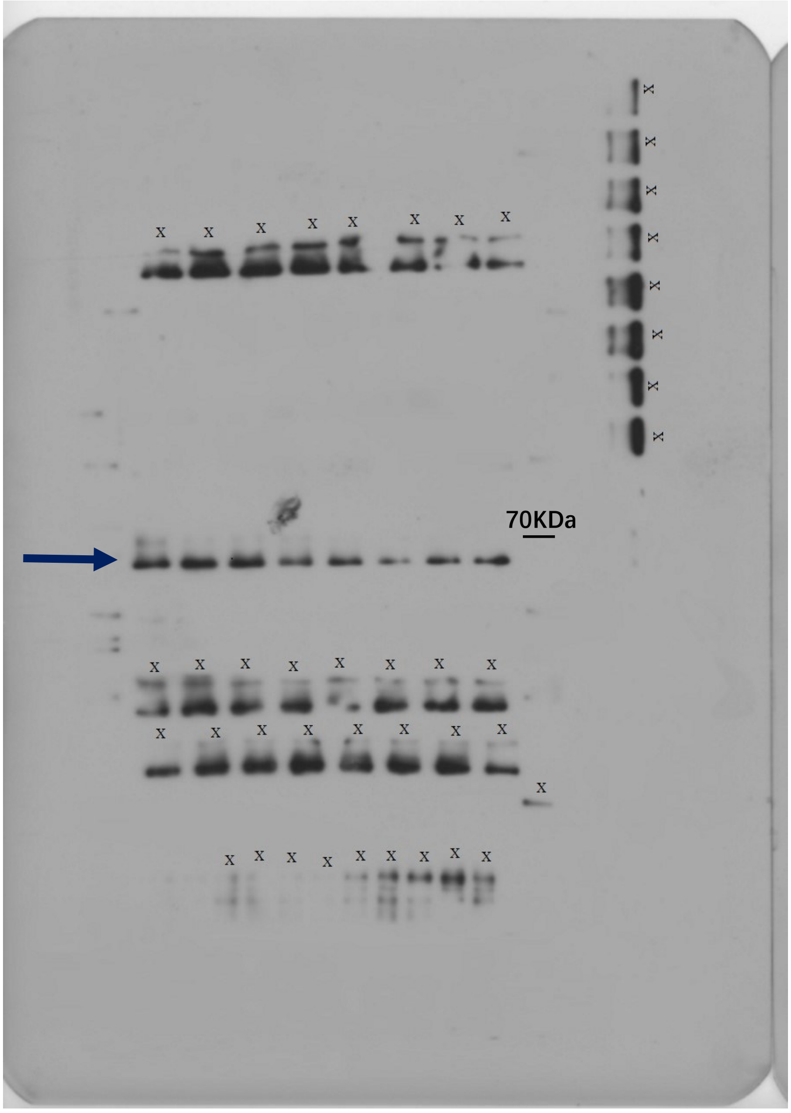

Loading order ⟶

Identity of experimental samples:

lane 1-4: Protein samples from the hippocampus of young (3M) mice 1-4; lane 5-8: Protein samples from the hippocampus of old (20M) mice 1-4.

Method used to capture the image:

Chemiluminescence. The bands were captured by developer and fixing solution on medical blue sensitive x-ray film (Baiyun, China), then the bands on the blots were scanned and analyzed with ImageJ (National Institutes of Health, Bethesda, MD, USA).

Actin

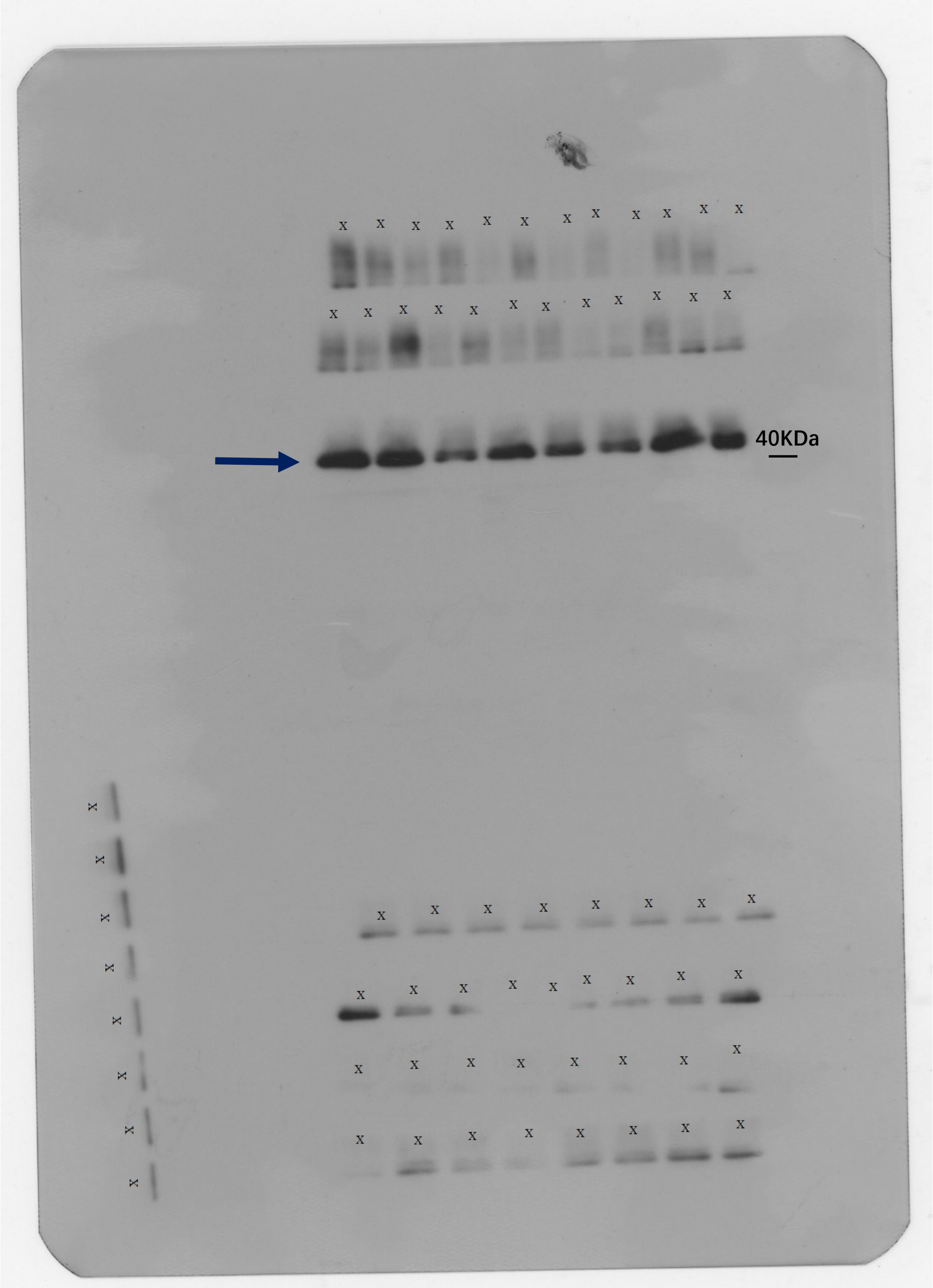

Loading order ⟶

Identity of experimental samples:

lane 1-4: Protein samples from the hippocampus of young (3M) mice 1-4; lane 5-8: Protein samples from the hippocampus of old (20M) mice 1-4.

Method used to capture the image:

Chemiluminescence. The bands were captured by developer and fixing solution on medical blue sensitive x-ray film (Baiyun, China), then the bands on the blots were scanned and analyzed with ImageJ (National Institutes of Health, Bethesda, MD, USA).

S1C _Fig

Menin

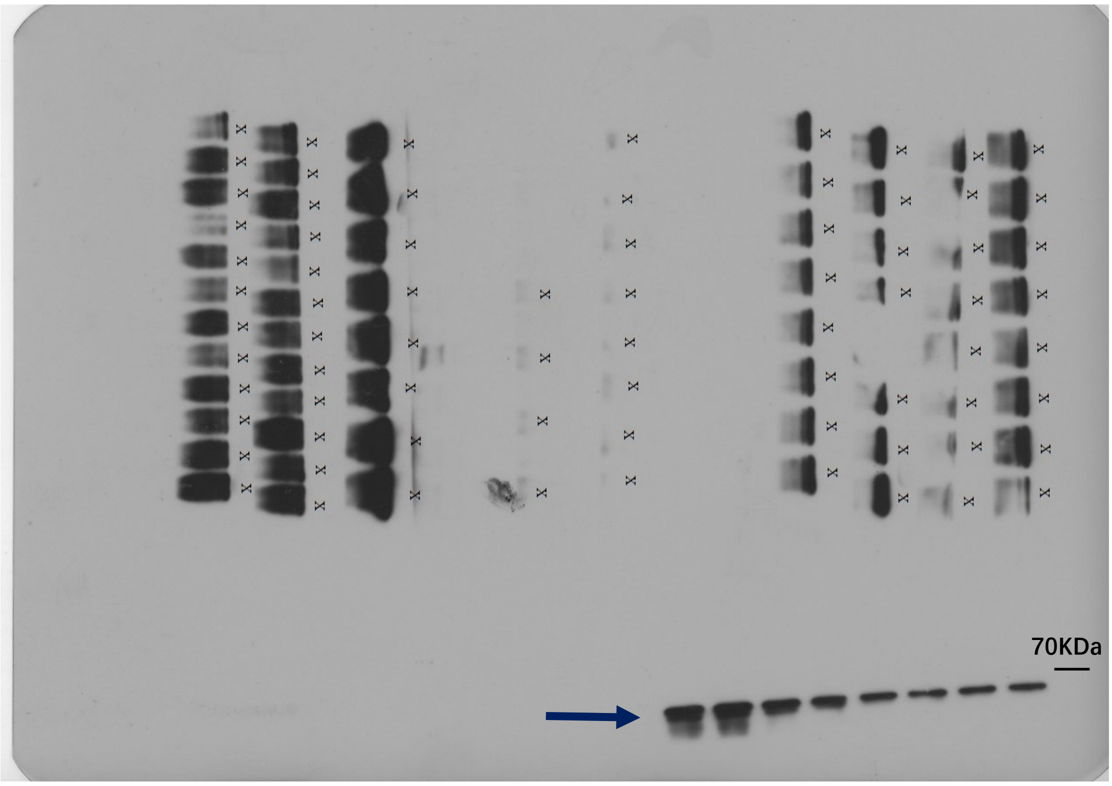

Loading order ⟶

Identity of experimental samples:

lane 1-4: Protein samples from the striatum of young (3M) mice 1-4; lane 5-8: Protein samples from the striatum of old (20M) mice 1-4.

Method used to capture the image:

Chemiluminescence. The bands were captured by developer and fixing solution on medical blue sensitive x-ray film (Baiyun, China), then the bands on the blots were scanned and analyzed with ImageJ (National Institutes of Health, Bethesda, MD, USA).

Actin

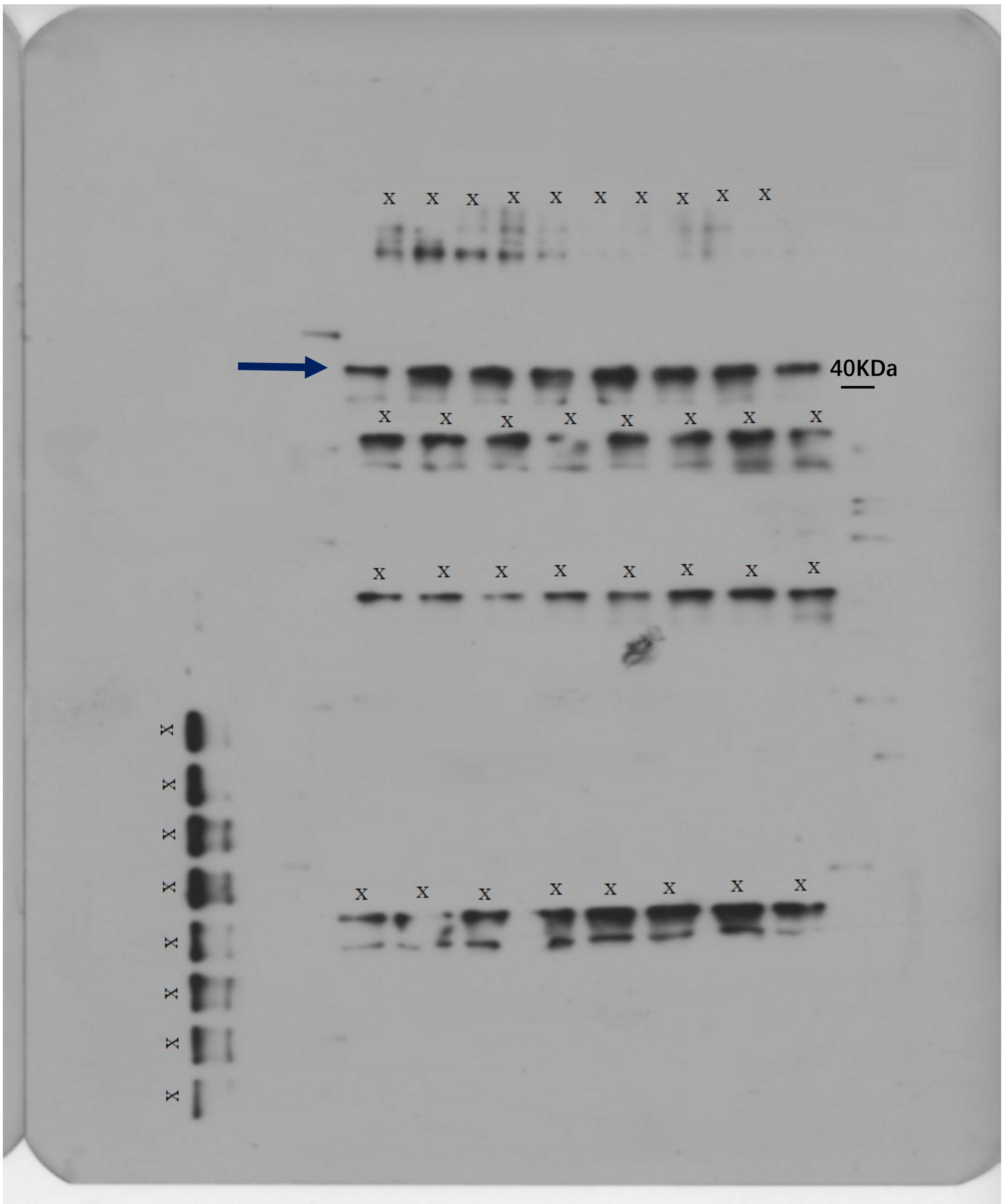

Loading order ⟶

Identity of experimental samples:

lane 1-4: Protein samples from the striatum of young (3M) mice 1-4; lane 5-8: Protein samples from the striatum of old (20M) mice 1-4.

Method used to capture the image:

Chemiluminescence. The bands were captured by developer and fixing solution on medical blue sensitive x-ray film (Baiyun, China), then the bands on the blots were scanned and analyzed with ImageJ (National Institutes of Health, Bethesda, MD, USA).

S1E _Fig

Menin

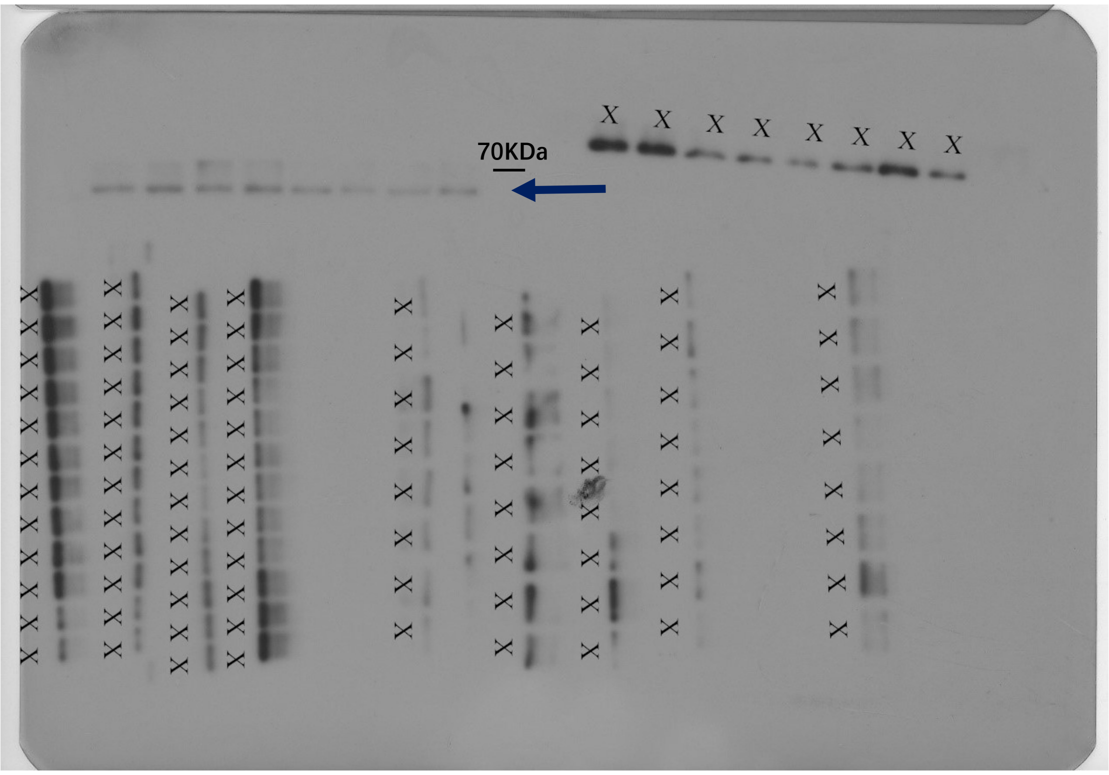

Loading order⟶

Identity of experimental samples:

lane 1-4: Protein samples from the prefrontal cortex of young (3M) mice 1-4; lane 5-8: Protein samples from the prefrontal cortex of old (20M) mice 1-4.

Method used to capture the image:

Chemiluminescence. The bands were captured by developer and fixing solution on medical blue sensitive x-ray film (Baiyun, China), then the bands on the blots were scanned and analyzed with ImageJ (National Institutes of Health, Bethesda, MD, USA).

Actin

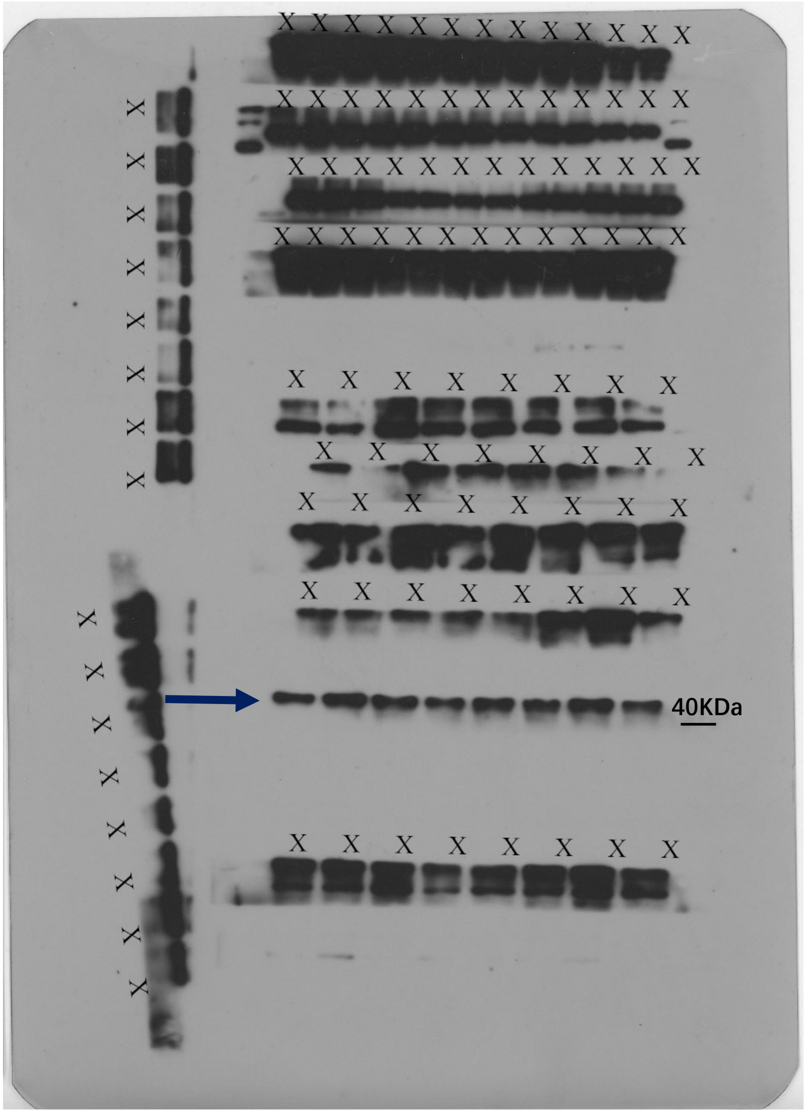

Loading order ⟶

Identity of experimental samples:

lane 1-4: Protein samples from the prefrontal cortex of young (3M) mice 1-4; lane 5-8: Protein samples from the prefrontal cortex of old (20M) mice 1-4.

Method used to capture the image:

Chemiluminescence. The bands were captured by developer and fixing solution on medical blue sensitive x-ray film (Baiyun, China), then the bands on the blots were scanned and analyzed with ImageJ (National Institutes of Health, Bethesda, MD, USA).

S1G _Fig

Menin

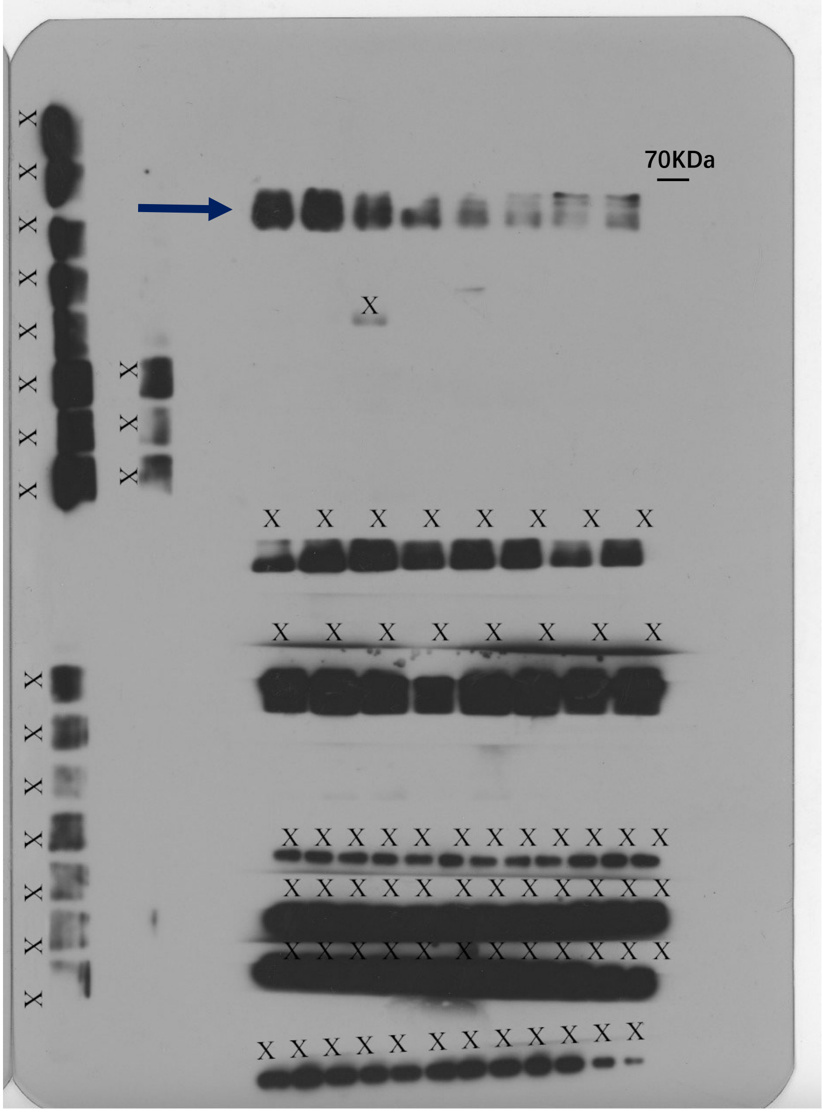

Loading order

Identity of experimental samples:

lane 1-4: Protein samples from the motor cortex of young (3M) mice 1-4; lane 5-8: Protein samples from the motor cortex of old (20M) mice 1-4.

Method used to capture the image:

Chemiluminescence. The bands were captured by developer and fixing solution on medical blue sensitive x-ray film (Baiyun, China), then the bands on the blots were scanned and analyzed with ImageJ (National Institutes of Health, Bethesda, MD, USA).

Actin

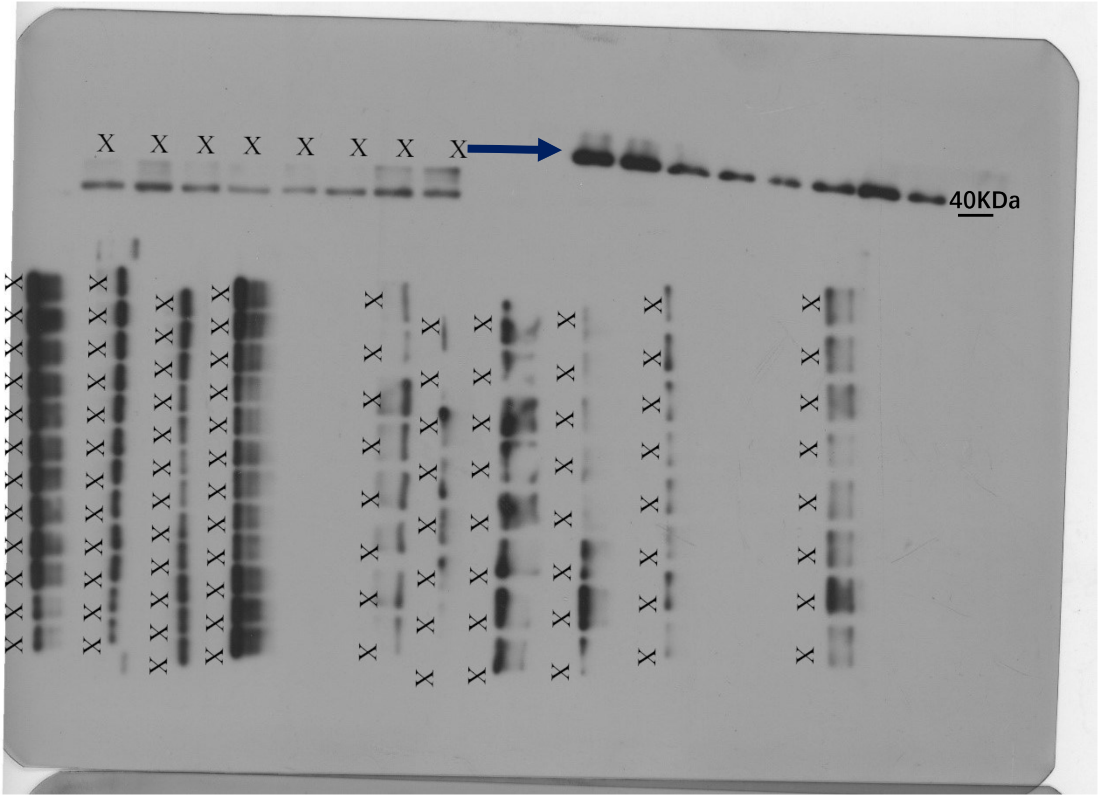

Loading order ⟶

Identity of experimental samples:

lane 1-4: Protein samples from the motor cortex of young (3M) mice 1-4; lane 5-8: Protein samples from the motor cortex of old (20M) mice 1-4.

Method used to capture the image:

Chemiluminescence. The bands were captured by developer and fixing solution on medical blue sensitive x-ray film (Baiyun, China), then the bands on the blots were scanned and analyzed with ImageJ (National Institutes of Health, Bethesda, MD, USA).

S1I _Fig

Menin

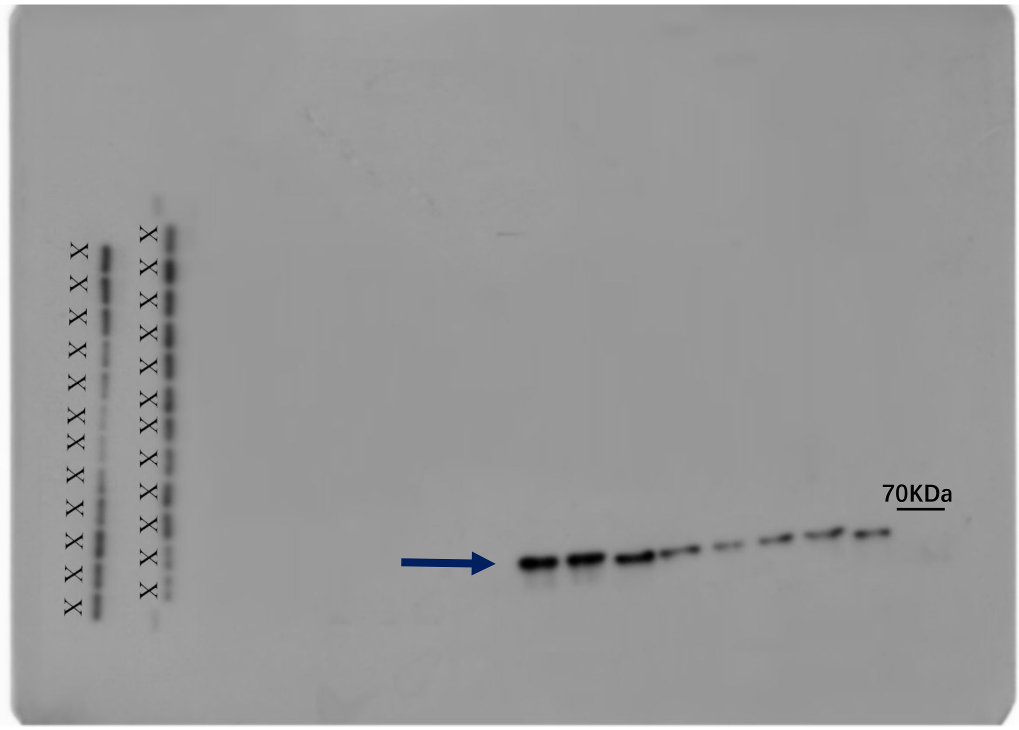

Loading order ⟶

Identity of experimental samples:

lane 1-4: Protein samples from the visual cortex of young (3M) mice 1-4; lane5-8: Protein samples from the visual cortex of old (20M) mice 1-4.

Method used to capture the image:

Chemiluminescence. The bands were captured by developer and fixing solution on medical blue sensitive x-ray film (Baiyun, China), then the bands on the blots were scanned and analyzed with ImageJ (National Institutes of Health, Bethesda, MD, USA).

Actin

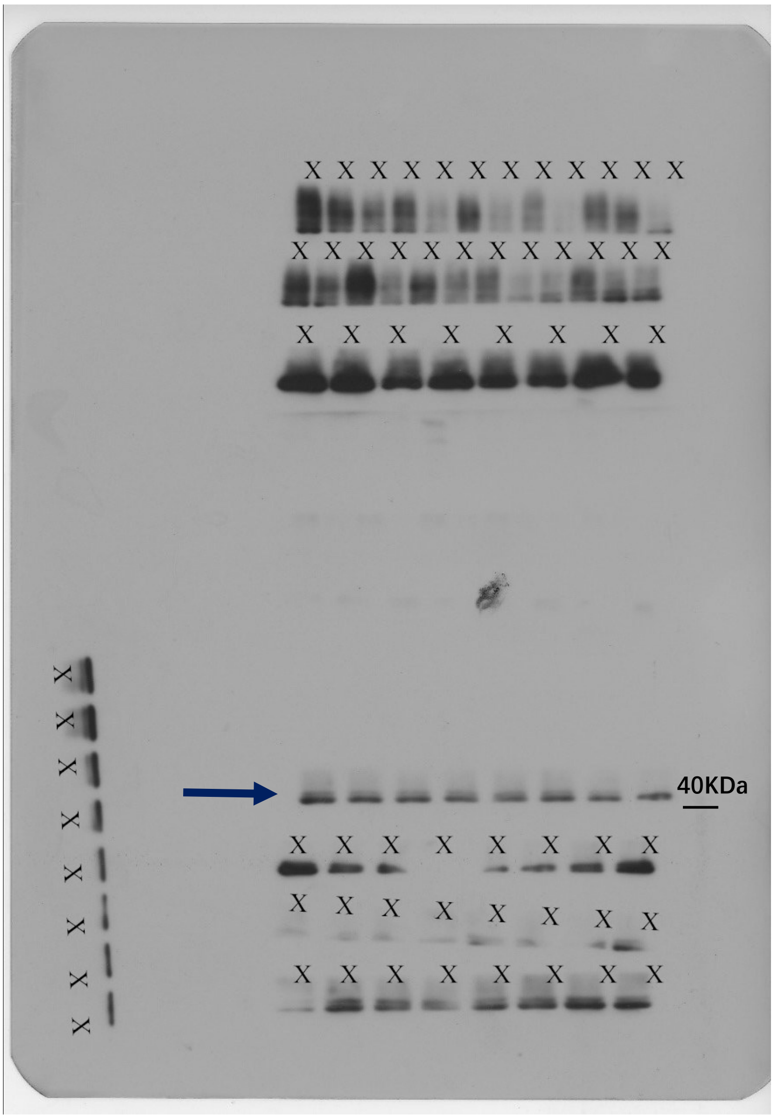

Loading order ⟶

Identity of experimental samples:

lane 1-4: Protein samples from the visual cortex of young (3M) mice 1-4; lane 5-8: Protein samples from the visual cortex of old (20M) mice 1-4.

Method used to capture the image:

Chemiluminescence. The bands were captured by developer and fixing solution on medical blue sensitive x-ray film (Baiyun, China), then the bands on the blots were scanned and analyzed with ImageJ (National Institutes of Health, Bethesda, MD, USA).

S1K _Fig

Menin

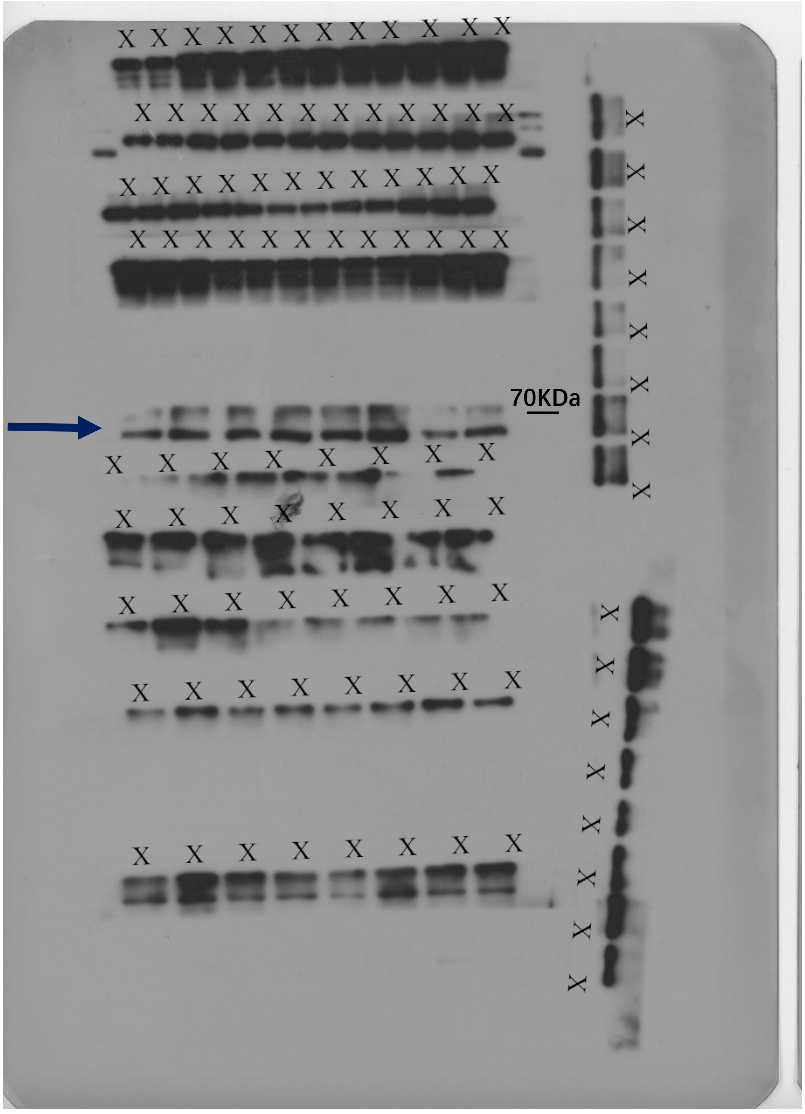

Loading order ⟶

Identity of experimental samples:

lane 1-4: Protein samples from the cerebellum of young (3M) mice 1-4; lane 5-8: Protein samples from the cerebellum of old (20M) mice 1-4.

Method used to capture the image:

Chemiluminescence. The bands were captured by developer and fixing solution on medical blue sensitive x-ray film (Baiyun, China), then the bands on the blots were scanned and analyzed with ImageJ (National Institutes of Health, Bethesda, MD, USA).

Actin

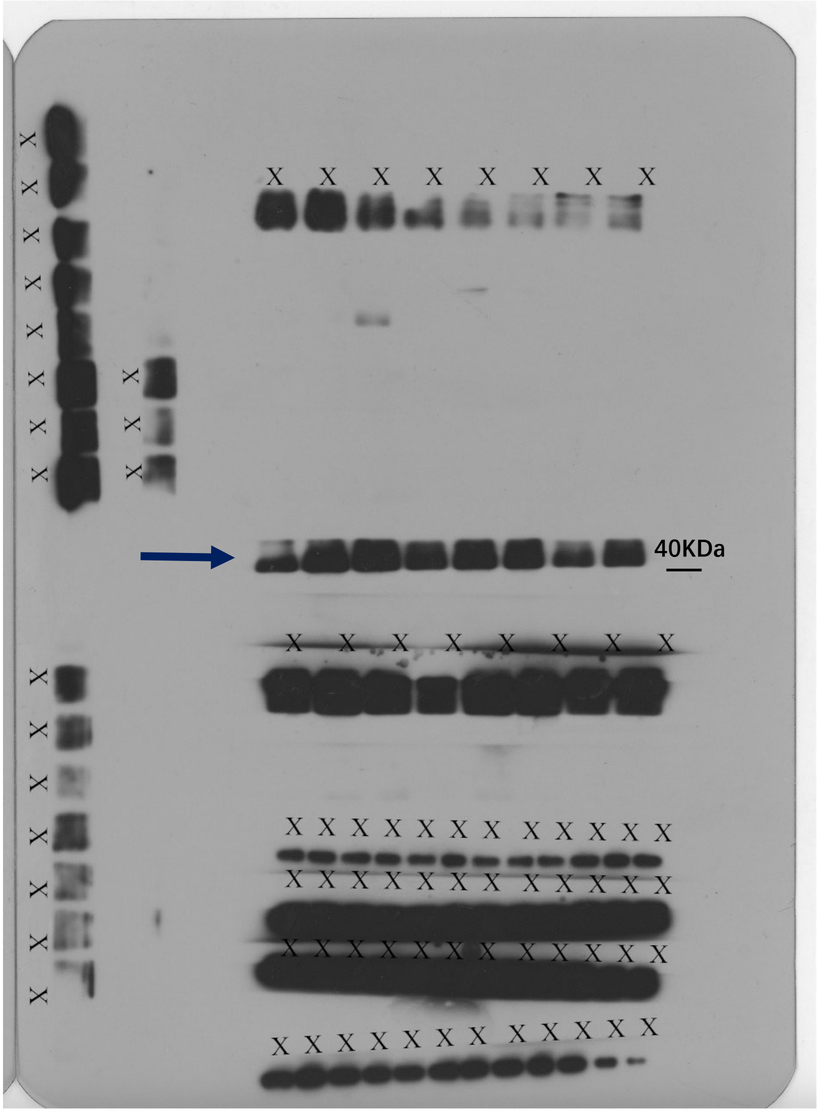

Loading order ⟶

Identity of experimental samples:

lane 1-4: Protein samples from the cerebellum of young (3M) mice 1-4; lane 5-8: Protein samples from the cerebellum of old (20M) mice 1-4.

Method used to capture the image:

Chemiluminescence. The bands were captured by developer and fixing solution on medical blue sensitive x-ray film (Baiyun, China), then the bands on the blots were scanned and analyzed with ImageJ (National Institutes of Health, Bethesda, MD, USA).

S1S _Fig

Menin

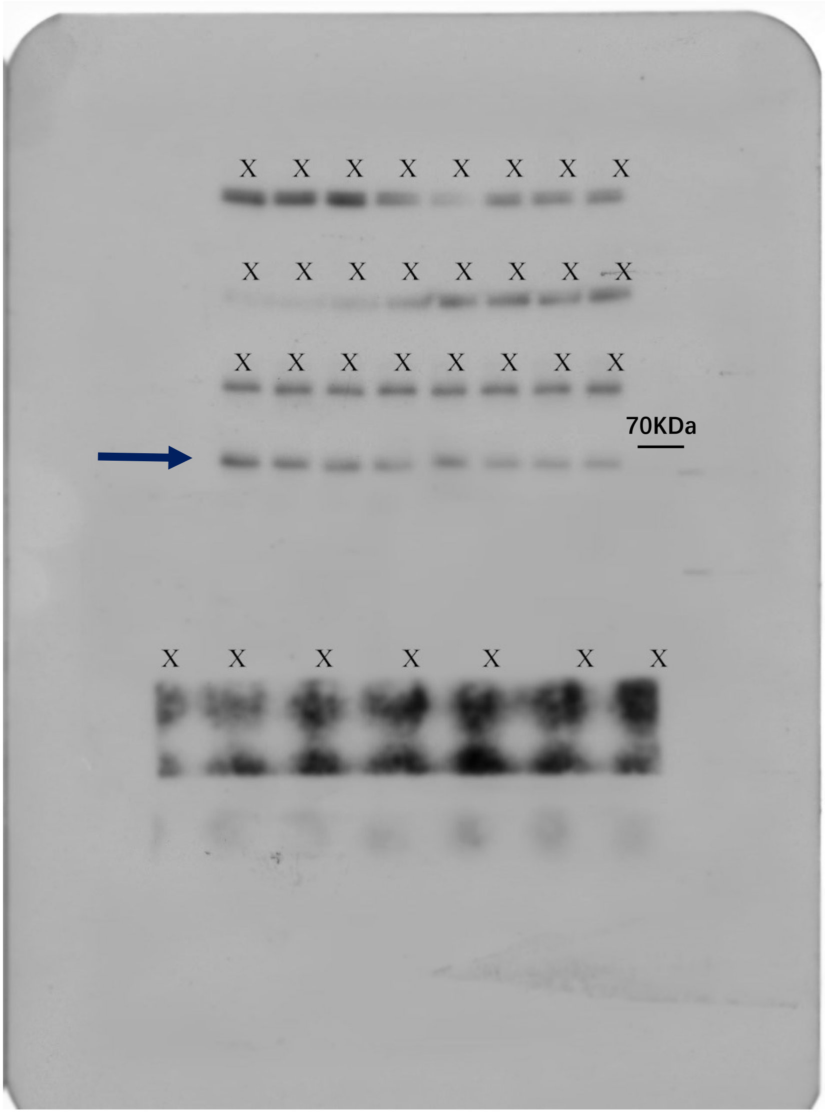

Loading order⟶

Identity of experimental samples:

lane 1-2: Protein samples from the hypothalamus of 3M wildtype mice 1-2; lane 3-4: Protein samples from the hypothalamus of 10M wildtype mice 1-2; lane 5-6: Protein samples from the hypothalamus of 16M wildtype mice 1-2; lane 7-8: Protein samples from the hypothalamus of 20M wildtype mice 1-2.

Method used to capture the image:

Chemiluminescence. The bands were captured by developer and fixing solution on medical blue sensitive x-ray film (Baiyun, China), then the bands on the blots were scanned and analyzed with ImageJ (National Institutes of Health, Bethesda, MD, USA).

Actin

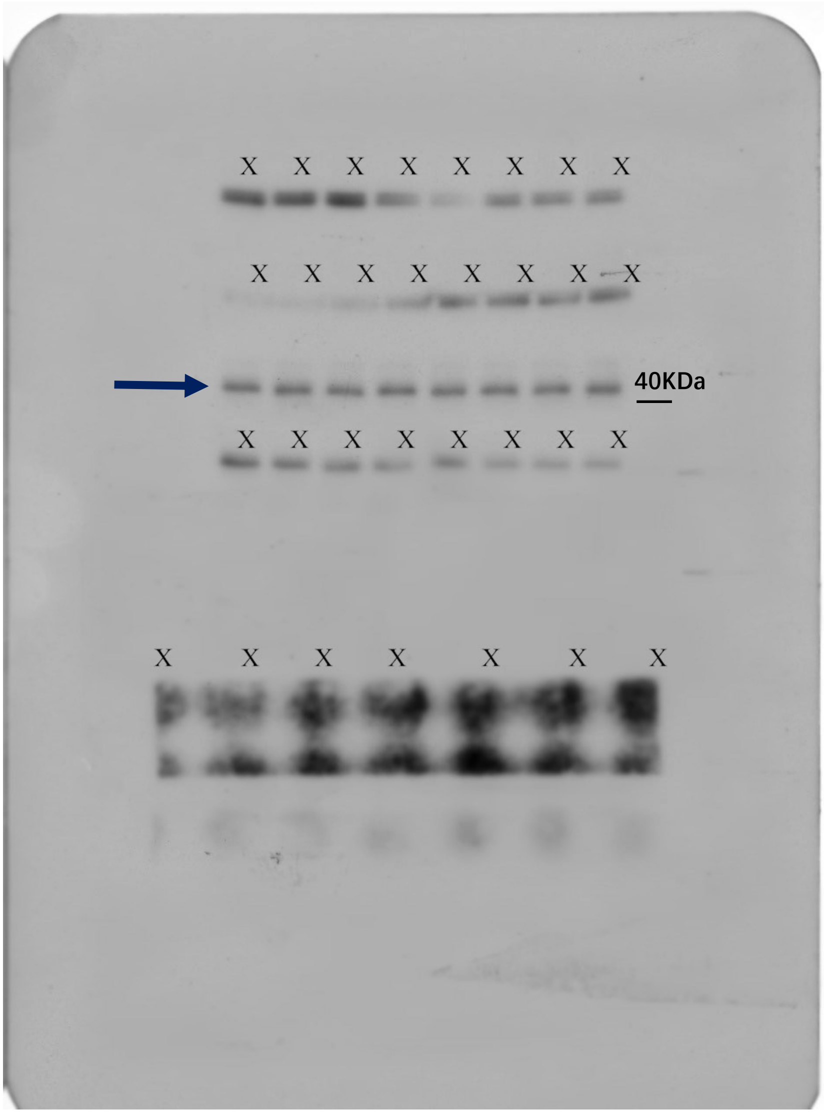

Loading order ⟶

Identity of experimental samples:

lane 1-2: Protein samples from the hypothalamus of 3M wildtype mice 1-2; lane 3-4: Protein samples from the hypothalamus of 10M wildtype mice 1-2; lane 5-6: Protein samples from the hypothalamus of 16M wildtype mice 1-2; lane 7-8: Protein samples from the hypothalamus of 20M wildtype mice 1-2.

Method used to capture the image:

Chemiluminescence. The bands were captured by developer and fixing solution on medical blue sensitive x-ray film (Baiyun, China), then the bands on the blots were scanned and analyzed with ImageJ (National Institutes of Health, Bethesda, MD, USA).

SourceData_Gel_ S4 _Fig

S4A _Fig

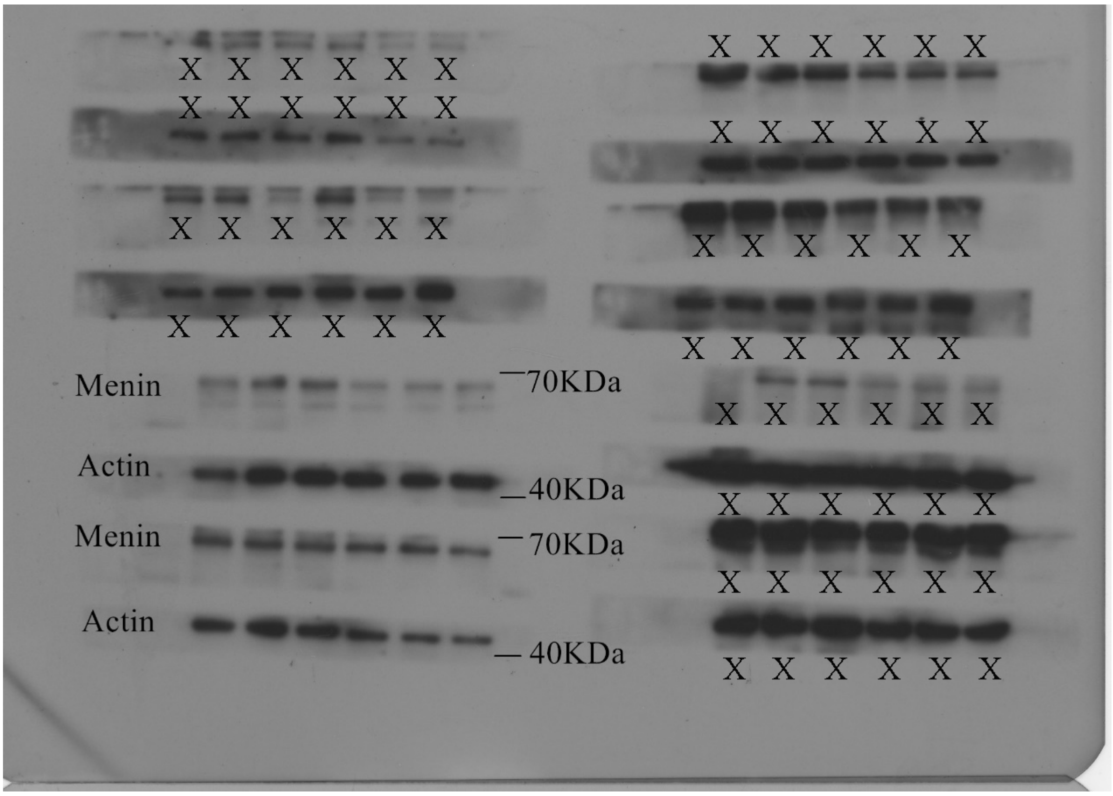

Loading order ⟶

Identity of experimental samples:

lane 1-3: Protein samples from the adrenal grand of 6M male wildtype mice 1-3; lane4-6: Protein samples from the adrenal grand of 6M male ScKO mice 1-3; lane 1-3: Protein samples from the adrenal grand of 6M male wildtype mice 1-3; lane 4-6: Protein samples from the adrenal grand of 6M male ScKO mice 1-3;

lane 1-3: Protein samples from the adrenal grand of 6M female wildtype mice 1-3; lane 4-6: Protein samples from the adrenal grand of 6M female ScKO mice 1-3;

lane 1-3: Protein samples from the adrenal grand of 6M female wildtype mice 1-3; lane 4-6: Protein samples from the adrenal grand of 6M female ScKO mice 1-3;

Method used to capture the image:

Chemiluminescence. The bands were captured by developer and fixing solution on medical blue sensitive x-ray film (Baiyun, China), then the bands on the blots were scanned and analyzed with ImageJ (National Institutes of Health, Bethesda, MD, USA).

S4C _Fig

Menin

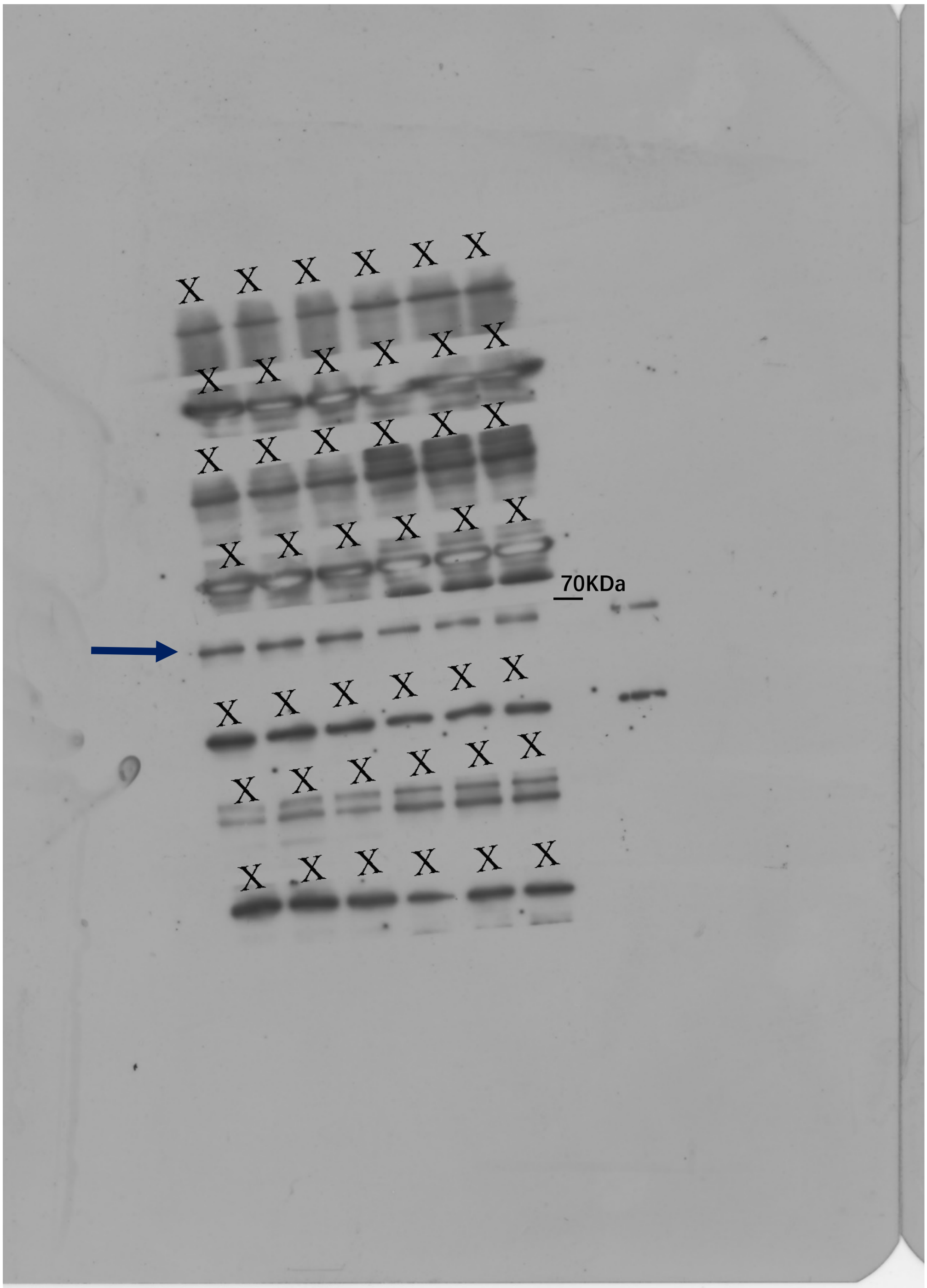

Loading order →

Identity of experimental samples:

lane 1-3: Protein samples from the pituitary of 6M male wildtype mice 1-3; lane 4-6: Protein samples from the pituitary of 6M male ScKO mice 1-3;

Method used to capture the image:

Chemiluminescence. The bands were captured by developer and fixing solution on medical blue sensitive x-ray film (Baiyun, China), then the bands on the blots were scanned and analyzed with ImageJ (National Institutes of Health, Bethesda, MD, USA).

Actin

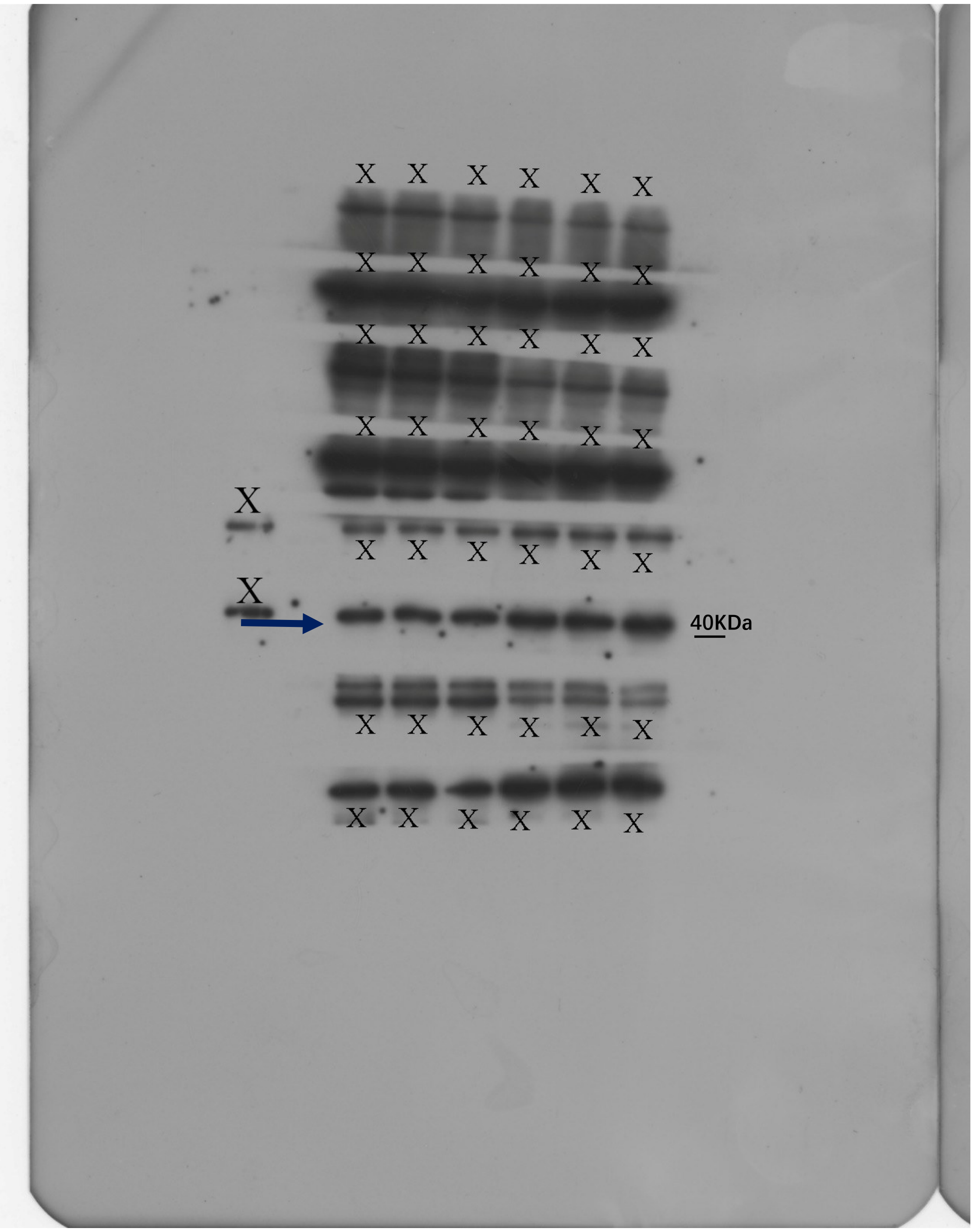

Loading order ⟶

Identity of experimental samples:

lane 1-3: Protein samples from the pituitary of 6M male wildtype mice 1-3; lane 4-6: Protein samples from the pituitary of 6M male ScKO mice 1-3.

Method used to capture the image:

Chemiluminescence. The bands were captured by developer and fixing solution on medical blue sensitive x-ray film (Baiyun, China), then the bands on the blots were scanned and analyzed with ImageJ (National Institutes of Health, Bethesda, MD, USA).

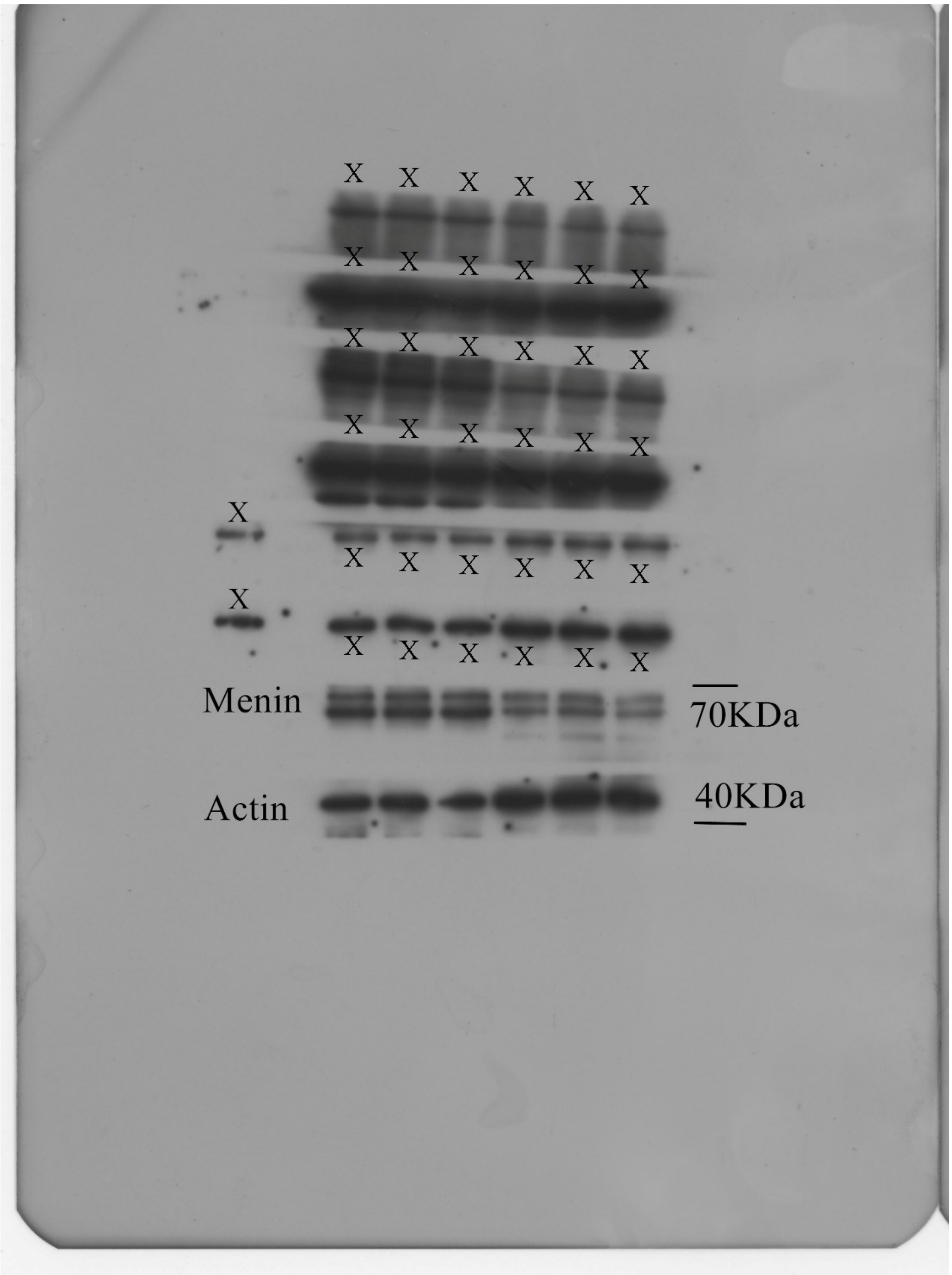

Loading order ⟶

Identity of experimental samples:

lane 1-3: Protein samples from the pituitary of 6M female wildtype mice 1-3; lane 4-6: Protein samples from the pituitary of 6M female ScKO mice 1-3;

lane 1-3: Protein samples from the pituitary of 6M female wildtype mice 1-3; lane 4-6: Protein samples from the pituitary of 6M female ScKO mice 1-3.

Method used to capture the image:

Chemiluminescence. The bands were captured by developer and fixing solution on medical blue sensitive x-ray film (Baiyun, China), then the bands on the blots were scanned and analyzed with ImageJ (National Institutes of Health, Bethesda, MD, USA).

S4E _Fig

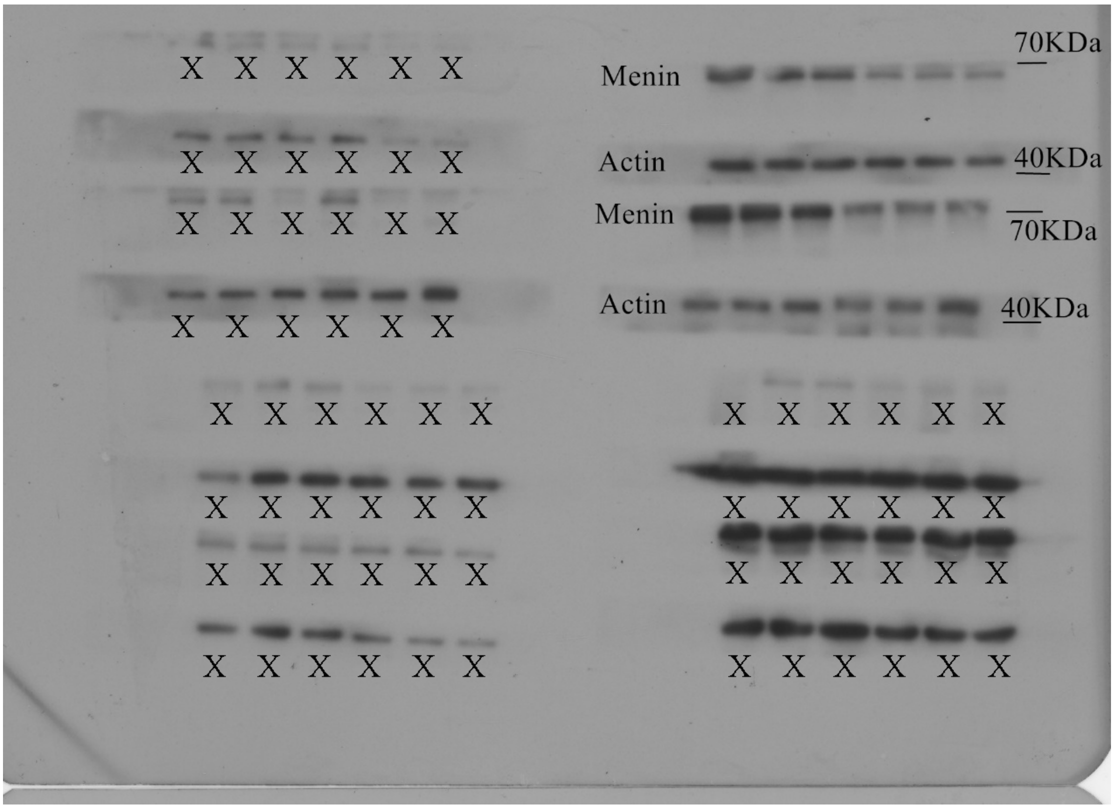

Loading order ⟶

Identity of experimental samples:

lane 1-3: Protein samples from the sexual grand of 6M male wildtype mice 1-3; lane 4-6: Protein samples from the sexual grand of 6M male ScKO mice 1-3;

lane 1-3: Protein samples from the sexual grand of 6M male wildtype mice 1-3; lane 4-6: Protein samples from the sexual grand of 6M male ScKO mice 1-3;

lane 1-3: Protein samples from the sexual grand of 6M female wildtype mice 1-3; lane 4-6: Protein samples from the sexual grand of 6M female ScKO mice 1-3;

lane 1-3: Protein samples from the sexual grand of 6M female wildtype mice 1-3; lane 4-6: Protein samples from the sexual grand of 6M female ScKO mice 1-3.

Method used to capture the image:

Chemiluminescence. The bands were captured by developer and fixing solution on medical blue sensitive x-ray film (Baiyun, China), then the bands on the blots were scanned and analyzed with ImageJ (National Institutes of Health, Bethesda, MD, USA).

S4G _Fig

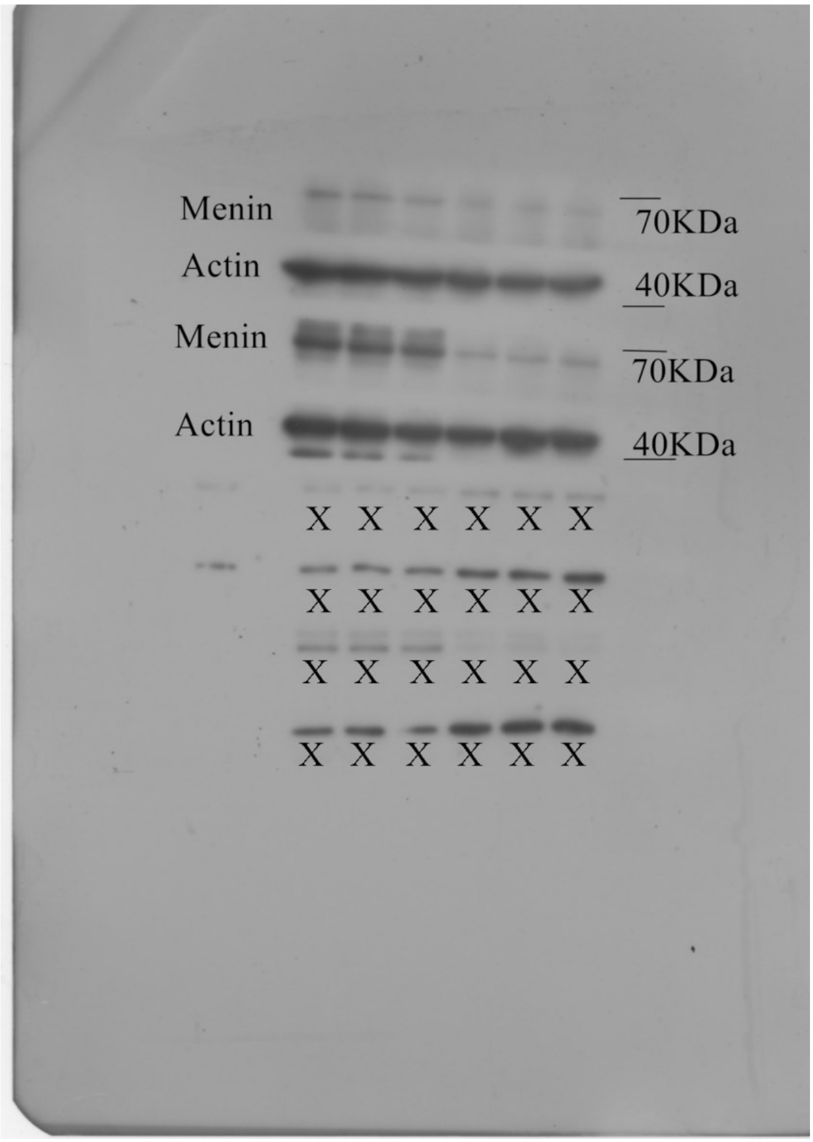

Loading order ⟶

Identity of experimental samples:

lane 1-3: Protein samples from the spleen of 6M male wildtype mice 1-3; lane 4-6: Protein samples from the spleen of 6M male ScKO mice 1-3;

lane 1-3: Protein samples from the spleen of 6M male wildtype mice 1-3; lane 4-6: Protein samples from the spleen of 6M male ScKO mice 1-3;

lane 1-3: Protein samples from the spleen of 6M female wildtype mice 1-3; lane 4-6: Protein samples from the spleen of 6M female ScKO mice 1-3;

lane 1-3: Protein samples from the spleen of 6M female wildtype mice 1-3; lane 4-6: Protein samples from the spleen of 6M female ScKO mice 1-3.

Method used to capture the image:

Chemiluminescence. The bands were captured by developer and fixing solution on medical blue sensitive x-ray film (Baiyun, China), then the bands on the blots were scanned and analyzed with ImageJ (National Institutes of Health, Bethesda, MD, USA)
